# Identification of effective alleles and haplotypes conferring pre-harvest sprouting resistance in winter wheat cultivars

**DOI:** 10.1186/s12870-022-03710-w

**Published:** 2022-07-06

**Authors:** Huang Yiwen, Dai Xuran, Liu Hongwei, Yu Shuo, Mai Chunyan, Yu Liqiang, Yu Guangjun, Yang Li, Zhou Yang, Li Hongjie, Zhang Hongjun

**Affiliations:** 1 National Engineering Research Center of Crop Molecular Breeding, Institute of Crop Sciences, Chinese Academy of Agricultural Sciences, Beijing, 100081 China; 2 College of Agronomy and Biotechnology, Hebei Normal University of Science & Technology, Qinhuangdao, 066004 China; 3 Xinxiang Innovation Center for Breeding Technology of Dwarf-Male-Sterile Wheat, Xinxiang, 453731 China; 4 Zhaoxian Experiment Station, Shijiazhuang Academy of Agricultural and Forestry Sciences, Zhaoxian, 051530 China

**Keywords:** *Triticum aestivum*, Pre-harvest sprouting resistance, Molecular marker-assisted selection, Functional marker, Germination index

## Abstract

**Background:**

Pre-harvest sprouting (PHS) is a serious limiting factor for wheat (*Triticum aestivum* L.) grain yield and end-use quality. Identification of reliable molecular markers and PHS-resistant germplasms is vital to improve PHS resistance by molecular marker-assisted selection (MAS), but the effects of allelic variation and haplotypes in genes conferring PHS resistance in winter wheat cultivars are less understood.

**Results:**

Resistance to PHS was tested in 326 commercial winter wheat cultivars for three consecutive growing seasons from 2018–2020. The effects of alleles and haplotypes of 10 genes associated with PHS resistance were determined for all cultivars and were validated by introgressing the PHS-resistance allele and haplotype into a susceptible wheat cultivar. High level of phenotypic variation in PHS resistance was observed in this set of cultivars and 8 of them were highly resistant to PHS with stable germination index (GI) of less than 25% in each individual year. Allelic effects of nine genes and *TaMFT* haplotype analysis demonstrated that the haplotype Hap1 with low-GI alleles at five positions had the best PHS resistance. This haplotype has the priority to use in improving PHS resistance because of its high effectiveness and rare present in the current commercial cultivars. Among 14 main allelic combinations (ACs) identified, the AC1 carrying the haplotype Hap1 and the *TaSdr-B1a* allele had better PHS resistance than the other classes. The introgression of Hap1 and *TaSdr-B1a* is able to significantly improve the PHS resistance in the susceptible cultivar Lunxuan 13.

**Conclusions:**

The effectiveness of alleles conferring PHS resistance in winter wheat cultivars was determined and the useful alleles and haplotypes were identified, providing valuable information for parental selection and MAS aiming at improving PHS-resistance in winter wheat. The identification of the PHS-resistant cultivars without known resistance alleles offers an opportunity to explore new PHS-resistant genes.

**Supplementary Information:**

The online version contains supplementary material available at 10.1186/s12870-022-03710-w.

## Background

Pre-harvest sprouting (PHS), a phenomenon that physiologically matured kernels germinate on their mother plants before harvesting due to high humidity [1], is a major constraint for grain yield and end-use quality of wheat (*Triticum aestivum* L.) [2]. Global estimate of the annual losses directly caused by PHS was about $1 billion [3]. In China, 83% of wheat planting areas suffer from PHS, mainly in the Middle and Lower Yangtze River Valleys Winter Wheat Zone (MLWZ), Southwestern Winter Wheat Zone (SWWZ) and Northeastern Spring Wheat Zone (NSWZ) [4]. In recent years, PHS frequently occurred in the Yellow and Huai River Valleys Winter Wheat Zone (YHWZ), resulting in serious reductions in grain yields in 2013, 2015, and 2016 [5]. Thus, resistance to PHS has become an important target trait in many wheat breeding programs.

Wheat grain color is associated with PHS, with better tolerance to PHS for the red-grained wheat cultivars than for the white-grained ones [6]. However, white-grained cultivars had higher flour yield than red-grained wheat cultivars at the same grade of whiteness of flour, which makes them more popular for millers [7, 8]. Most Chinese prefer the shine and white color of products, mainly including steamed bread, boiled dumpling and noodle. The production acreages of white-grained wheats increase due to the strong market demand. Among the 75 wheat cultivars with the annual planting area > 667,000 hectares during the last several decades in China, 61 are white grains [9]. Hence, improvement of white-grained wheat with strong resistance to PHS is important for expanding its production in order to meet the market demands.

Resistance to PHS is a complex quantitative trait that is controlled by both genetic factors and external environmental factors [10]. Marker-assisted selection (MAS) on PHS resistance can not only shorten breeding cycles but also enhance selection efficiency. To date, more than 42 quantitative trait loci (QTL) governing PHS resistance are cataloged in wheat, most of which are associated with grain color and seed dormancy [2, 11, 12]. The transcription factor *Tamyb10* is a candidate gene of grain color-related gene *R*, and three homoeologous genes *Tamyb10-A1*, *Tamyb10-B1*, and *Tamyb10-D1* were identified [9, 13–15]. Gene *TaDFR-B* affects grain color and PHS resistance by controlling anthocyanin synthesis, and the *TaDFR-Bb* allele was tightly associated with PHS resistance in *TaDFR-B* [16].

Seed dormancy is another genetic factor affecting PHS resistance [17, 18]. Gene *TaMFT*, a wheat homolog of *TaPHS1* related to seed dormancy, was cloned from red-grained cultivar Zen [19], and white cultivar Rio Blanco [20]. Five SNP mutations or InDels associated with low germination index (GI) were characterized in the promoter and coding regions of *TaMFT* [19, 21–23]. *TaMKK3-A*, previously known as *Phs1*, on chromosome 4A is another major gene governing seed dormancy [18]. The PHS-resistant and -susceptible cultivars carry the *TaMKK3-Aa* and *TaMKK3-Ab* alleles in *TaMKK3-A*, respectively [24]. The seed dormancy-related genes *TaSdr*, *TaVp-1*, and *TaGASR34* were cloned by means of the homology-based cloning approach [25–29], and the corresponding functional markers have been developed.

Resistance to PHS can be assessed under field and controlled environmental conditions [23]. Field evaluation of PHS resistance needs suitable weather conditions including humidity and temperature, hence, it is difficult to obtain consistent phenotypic data in different years or environments. By contrast, controlled environmental evaluation of PHS resistance is relatively easy, and phenotypic data of PHS can be repeated in other environments. Sprouting rate of whole spikes and seed germination test are two of the main methods under controlled environment [30, 31]. Calculation of the GI by testing seed germination is the most direct approach to detect seed dormancy, and was widely used to evaluate PHS resistance in previous studies [27–29]. However, improvement of PHS resistance based on phenotypic selection is time-consuming and labor-intensive.

The reliable molecular markers are a prerequisite for MAS, but PHS resistance and effects of allelic variation and haplotypes in known genes affecting PHS resistance in winter wheat cultivars are less studied. The aims of this study were to 1) evaluate PHS resistance in a set of winter wheat cultivars in China; 2) identify the allelic variation and haplotypes of 10 PHS resistance genes and compare the effects of contrasting alleles at each gene, haplotypes of *TaMFT*, and allelic combinations on PHS resistance; and 3) validate the effects of PHS resistance allele and haplotype under the genetic background of a PHS-susceptible wheat cultivar.

## Materials and methods

### Plant materials

An array of 326 Chinese winter wheat cultivars released from 1964 to 2020, including 19 cultivars from the Northern Winter Wheat Zone (NWWZ), 270 from the YHWZ, 21 from the MLWZ, and 16 from the SWWZ, were used in this study. These cultivars originated from 13 provinces, i.e., Beijing (14), Ningxia (1), Shanxi (5), Tianjin (1), Xinjiang (2), Hebei (60), Shandong (50), Henan (128), Shaanxi (10), Anhui (5), Hubei (4), Jiangsu (30), and Sichuan (16). All cultivars were approved by the National Genebank (Beijing, China) and wheat breeders. Seeds of these cultivars were originally acquired from the National Genebank, Beijing, or breeders. The detailed information of each cultivar is listed in Table S1.

The F_2_ (206 plants) and derived F_3_ (206 lines) populations from the cross Lunxuan 13 (*TaMFT*-Hap4*/TaSdr-B1b*, susceptible to PHS) × Bainong 3217 (*TaMFT*-Hap1*/TaSdr-B1a*, resistant to PHS) were used to validate the PHS resistance of alleles in *TaSdr-B1*, haplotypes of *TaMFT*, and their combinations. Both Bainong 3217 [Funo/Neixiang 5//Xiannong 39/3/Xinong 64(4)3/Yanda 24] and Lunxuan 13 (Shimai 12/Zhoumai 16//Zhoumai 16) are white-grained cultivars but differ in the resistance to PHS.

### Field trials

The whole set of wheat cultivars were planted at the experimental station of the Chinese Academy of Agricultural Sciences (CAAS) in Xinxiang (35°31′N, 113°85′E), Henan province, during the 2017–2018, 2018–2019, and 2019–2020 wheat cropping seasons. All cultivars were arranged in one-row plots of 2-m length and 0.25-m width with 40 seeds. The soil of field is a typical clay loam. Fertilization, irrigation, and other field managements were carried out as described previously [32]. The meteorological data of daily average temperature (°C), relative humidity (%) and precipitation (mm) of the three cropping seasons is showed in Fig. S1.

### Germination index assay

Resistance of the wheat entries to PHS was assessed using the seed GI method for three years from 2018–2020. The GIs of 206 F_2_ plants and derived F_3_ lines from the Lunxuan 13 × Bainong 3217 population were assessed in 2019 (F_2_) and 2020 (F_3_), respectively. The F_2_ plants were individually harvested and separately evaluated of GI. About 30 spikes were harvested from each cultivar and F_3_ line at the physiological maturity stage (about 35 d after flowering), air dried at ambient temperature (~ 25 °C) for 3 d, hand-threshed, and stored in a refrigerator at -20 °C. Fifty seeds were sterilized with 5% NaClO, evenly embedded on two layers of filter paper in Petri dishes (15 cm in diameter), and incubated in a growth cabinet at 25 °C for 7 d with 50 mL of sterile water. Germinated seeds were counted daily and removed. Germination index was calculated according to the method described by Zhang et al. [27]. This experiment was carried out thrice.

### Genotyping

The alleles of 10 genes associated with PHS resistance were determined using the functional markers as described in Table S2. For the sequence tagged site (STS) and cleaved amplified polymorphic sequence (CAPS) markers, a 20 μL reaction mixture was prepared by mixing 1 μL of 50–100 ng μL^−1^ template DNA, 1 μL each of the forward and reverse primers (10 μM), 10 μL 2 × *Taq* PCR Master Mix (P111-03, Nanjing Vazyme Biotech Co. Ltd., Nanjing, China), and 7 μL of sterilize ddH_2_O. Amplification of DNA was performed in a SimpliAmp thermal cycler (Thermo Fisher Scientific (China) Co., Ltd., Shanghai, China). The Kompetitive Allele-Specific PCR (KASP) assays were carried out in a 5 μL reaction mixture including 2.5 μL PARMS SNP master mix (GTE001-2, Wuhan Genetides Biotech Co., Ltd., Wuhan, China), 0.056 μL primer mix, 0.04 μL Mg^2+^, 2.2 μL template DNA (20–50 ng μL^−1^) and 0.204 μL ddH_2_O using a BIO-RAD S1000 Thermal Cycler PCR System (Bio-Rad Laboratory Inc., Hercules, CA, USA). Genotyping of KASP markers was performed following the protocol as described by Rasheed et al. [33].

### Data analysis

Analysis of variance (ANOVA) for the GI values of 326 winter wheat cultivars over three years was performed using the PROC GLM program in the Statistical Product and Service Solutions (SPSS) software 22.0 (International Business Machines Corporation, Armonk, New York, USA) [34]. Phenotypic comparison between white-grained and red-grained wheats, and differences of PHS resistance between contrasting alleles of each gene were determined by the *t*-test in SPSS software 22.0. Multiple comparisons (PROC GLM) for the GI values of wheat cultivars from different wheat zones or provinces and phenotypic differences among haplotypes or allelic combinations (ACs) were performed using Tukey–Kramer at *P* < 0.05 in SPSS software 22. The broad-sense heritability (*h*^*2*^) and correlation coefficients between years were estimated according to the method described by Li et al. [35].

## Results

### Phenotypic evaluation on PHS resistance

The mean squares of genotypes, years and genotype × year interaction were significant as shown by ANOVA (*P* < 0.01) (Table S3). The broad-sense heritability (*h*^*2*^) of GI was 0.96. There were significant differences of GI in different years, and the mean GI values of the 326 cultivars were 48.9%, 59.2%, and 34.5% in 2018, 2019 and 2020, respectively. The wide range of phenotypic variation in GI was observed in each of the three years (Fig. S2a). A total of 43, 10 and 86 cultivars showed the GI values lower than 25.0% in 2018, 2019 and 2020, respectively (Fig. S2a, Table S4), and 8 cultivars had stable PHS resistance across the three years.

The difference in GI was significant among the four wheat zones, in which MLWZ had the lowest GI value in each year (Fig. S2b). The cultivars from Jiangsu and Shaanxi provinces showed better PHS resistance than those from the other provinces (Fig. S2c). Grain color was associated with PHS resistance (Fig. S2d), and the red-grained cultivars had lower mean GI than the white-grained cultivars (*P* < 0.05). The GI values for the 326 cultivars measured in different years were significantly correlated with a range of correlation coefficients from 0.69 to 0.79 (Fig. S3).

### Allelic and haplotypic frequencies

**Fig. 1:**
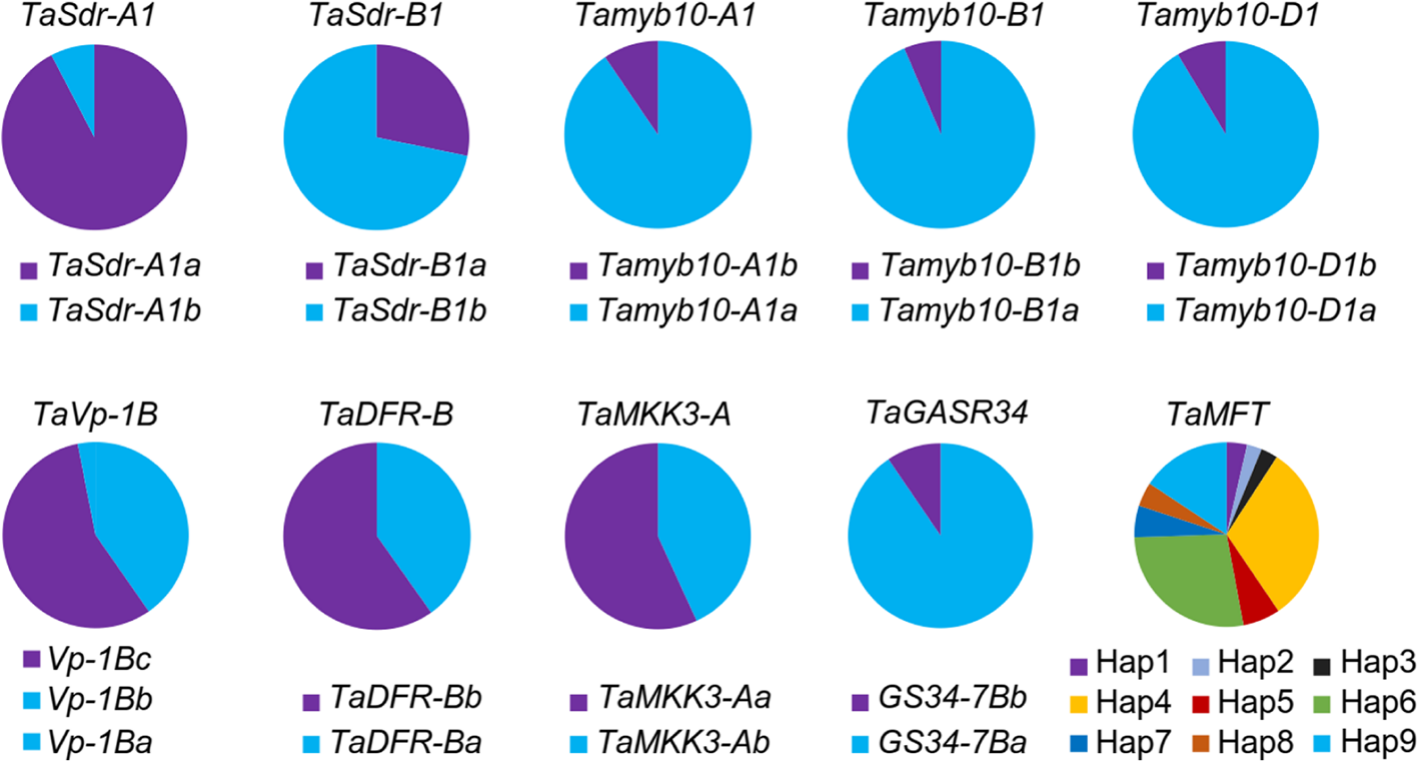
Allelic and haplotypic frequencies of 10 genes associated with pre-harvest sprouting resistance in the 326 winter wheat cultivars. Purple and blue boxes indicate pre-harvest sprouting (PHS) resistance and susceptibility alleles/haplotypes, respectively. The allelic composition of *TaMFT* haplotypes refers to Table 1

Allelic variation of 9 genes and haplotypes of *TaMFT* in the 326 cultivars were determined (Table S4). The mean frequencies of alleles conferring low GI and high GI were 36.4% and 63.6%, respectively (Fig. 1). The low-GI alleles had higher frequencies than the corresponding high-GI alleles in *TaSdr-A1*, *TaVp-1B*, *TaDFR-B* and *TaMKK3-A*. Among them, the frequency of *TaSdr-A1a* allele only was over 60.0%. Nine main *TaMFT* haplotypes (Hap1-Hap9) were identified based on the genotypes at the positions- 194,- 222, + 219, + 646, + 666 in the 326 winter wheat cultivars (Table 1). Haplotypes Hap4 (29.4%) and Hap6 (25.8%) had the higher frequencies than the other haplotypes, whereas the frequency of Hap1 with five PHS resistance allele was only 3.4% (Fig. 1).

**Table 1:** Main *TaMFT* haplotypes identified in the 326 wheat cultivars

Position	Allele	Hap1	Hap2	Hap3	Hap4	Hap5	Hap6	Hap7	Hap8	Hap9
- 194	*** TaMFT-3Aa****/b*	+	*–*	+	+	*–*	+	+	*–*	*–*
- 222	*** TaPHS1-222C****/T*	+	*–*	*–*	*–*	*–*	*–*	*–*	*–*	*–*
+ 219	*** TaMFT-A1b****/a*	+	+	+	+	*–*	*–*	*–*	*–*	*–*
+ 646	*** TaPHS1-646G****/A*	+	+	*–*	+	+	+	*–*	+	*–*
+ 666	*** TaPHS1-666A****/T*	+	+	*–*	+	+	+	*–*	*–*	*–*

+ : Pre-harvest sprouting (PHS) resistance alleles (bold alleles); –: *PHS* susceptibility allele

### Allelic effects and haplotype analysis

Cultivars carrying the low-GI allele showed better PHS resistance than those carrying the high-GI allele in each of the 9 genes identified (Table 2). Among them, cultivars with the low-GI alleles *TaSdr-B1a* and *Tamyb10-D1b* had significantly lower GI values than those with the contrasting high-GI alleles in *TaSdr-B1* and *Tamyb10-D1* in the three years, respectively (*P* < 0.05). The allele *Tamyb10-D1b* had the largest phenotypic effect on PHS resistance at the single gene level. Compared to *Tamyb10-D1a*, the allele *Tamyb10-D1b* decreased GIs by 10.4%, 14.9% and 10.6 in 2018, 2019 and 2020, respectively.

**Table 2:** Comparison of germination index (GI) between contrasting alleles at each gene in the 326 winter wheat cultivars for the three years

Gene	Allele	Mean ± SD (%)			
		2018	2019	2020	Mean
* TaSdr-A1*	*** TaSdr-A1a***	48.7 ± 19.9a	59.1 ± 17.6a	34.3 ± 18.6a	47.4 ± 17.1a
	* TaSdr-A1b*	50.3 ± 19.9a	59.8 ± 18.6a	35.1 ± 20.1a	48.4 ± 17.5a
* TaSdr-B1*	*** TaSdr-B1a***	42.6 ± 20.4a	52.8 ± 18.4a	29.5 ± 18.0a	41.6 ± 17.5a
	* TaSdr-B1b*	51.3 ± 19.1b	61.7 ± 16.8b	36.5 ± 18.6b	49.8 ± 16.4b
* Tamyb10-A1*	* Tamyb10-A1a*	49.4 ± 19.2a	59.9 ± 16.9a	34.9 ± 18.4a	48.1 ± 16.5a
	*** Tamyb10-A1b***	43.8 ± 24.8a	52.2 ± 22.9a	30.6 ± 20.7a	42.2 ± 21.7a
* Tamyb10-B1*	* Tamyb10-B1a*	48.9 ± 19.5a	59.5 ± 17.5a	35.1 ± 18.7a	47.8 ± 16.9a
	*** Tamyb10-B1b***	47.8 ± 24.1a	54.5 ± 19.7a	26.1 ± 16.1a	42.8 ± 18.8a
* Tamyb10-D1*	* Tamyb10-D1a*	49.7 ± 19.1b	60.5 ± 16.7b	35.4 ± 18.3b	48.5 ± 16.3b
	*** Tamyb10-D1b***	39.3 ± 24.6a	45.6 ± 21.9a	24.8 ± 19.5a	36.6 ± 21.3a
* TaVp-1B*	* Vp-1Ba*	50.0 ± 20.4a	60.2 ± 18.2a	36.8 ± 19.4a	49.0 ± 17.8a
	* Vp-1Bb*	50.5 ± 19.1a	66.0 ± 18.1a	41.8 ± 24.7a	52.8 ± 19.6a
	*** Vp-1Bc***	48.0 ± 19.5a	58.3 ± 17.2a	32.4 ± 17.6a	46.2 ± 16.3a
* TaDER-B*	*** TaDER-Bb***	47.2 ± 19.1a	58.7 ± 16.8a	30.0 ± 17.3a	46.6 ± 15.9a
	* TaDER-Ba/c*	51.3 ± 20.8a	60.0 ± 18.9a	35.2 ± 20.6a	48.8 ± 18.7a
* TaMKK3-A*	*** TaMKK3-Aa***	47.9 ± 19.8a	58.5 ± 17.6a	33.7 ± 17.2a	46.7 ± 16.5a
	* TaMKK3-Ab*	50.2 ± 19.9a	60.3 ± 17.7a	35.4 ± 20.3a	48.6 ± 17.7a
* TaGASR34*	* GS34-7Ba*	49.6 ± 19.1b	59.7 ± 16.7b	34.6 ± 17.9a	48.0 ± 16.2a
	*** GS34-7Bb***	42.2 ± 24.7a	53.6 ± 24.8a	33.2 ± 25.3a	43.0 ± .23.9a

Bold alleles represent PHS resistance ones

*SD* standard deviation. Different letters within column indicate significant difference between contrasting alleles at each gene at *P* < 0.05

**Fig. 2:**
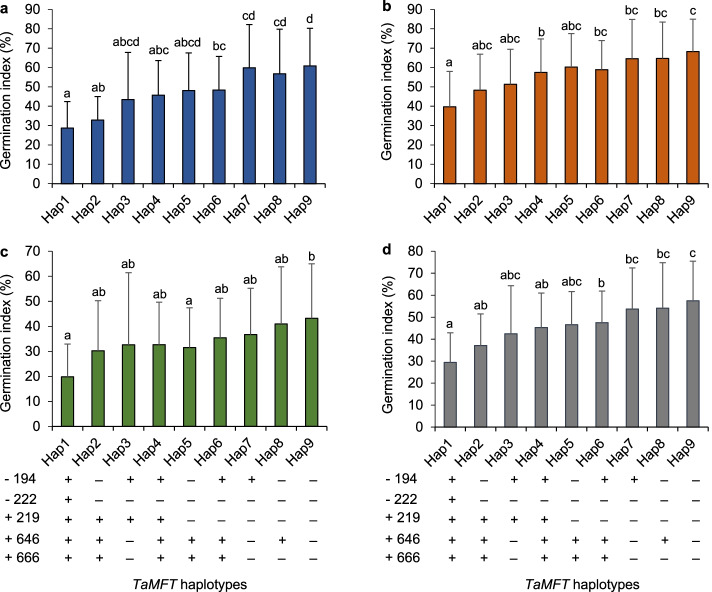
Effects of *TaMFT* haplotypes on germination index (GI) in 2018 (**a**), 2019 (**b**), 2020 (**c**), and mean value (**d**). “ + ” and “–”, the PHS resistance allele and susceptibility allele, respectively. Different letters indicate significance of GI among haplotypes at *P* < 0.05

Haplotypes Hap1-Hap8 with at least one PHS resistance allele at five positions exhibited lower GIs than the Hap9 with five PHS susceptibility alleles (Fig. 2). Among them, Hap1 showed stable and better PHS resistance than the other haplotypes in each individual year. Compared to haplotype Hap9, Hap1 had significantly lower GIs, and averagely decreased by 32.1%, 28.5%, and 23.3% in 2018, 2019, and 2020, respectively (*P* < 0.05).

### Effects of allelic combinations

**Fig. 3:**
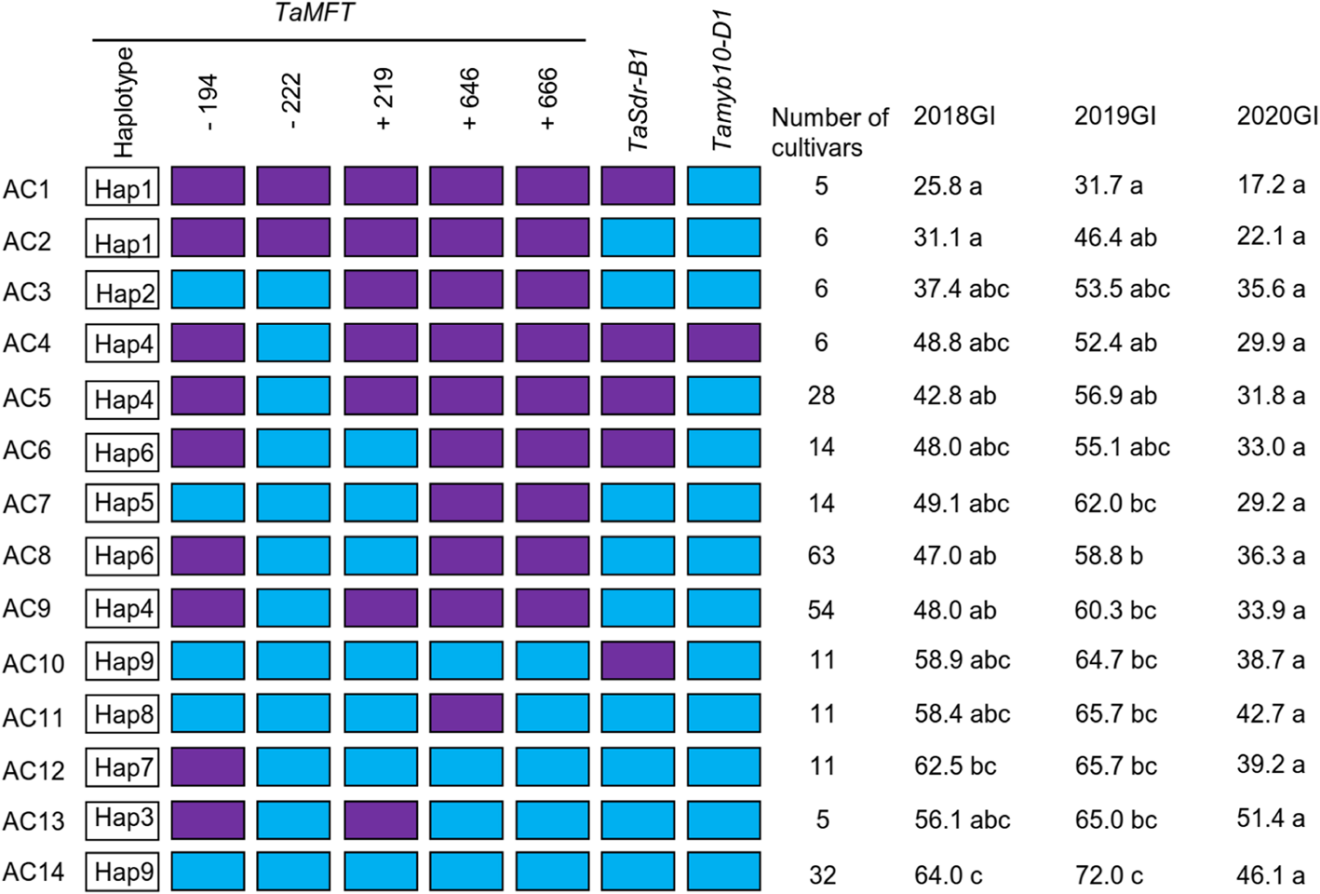
Comparison of germination index (GI) among 14 main allelic combinations (ACs) in the three pre-harvest sprouting (PHS) genes in the three years. Purple and blue boxes indicate pre-harvest sprouting (PHS) resistance and susceptibility alleles, respectively. Different letters after GIs in each year indicate significant differences at *P* < 0.05

Among the 10 genes associated with PHS resistance analyzed, 3 genes had significant difference in GIs between the contrasting alleles or among haplotypes. Hence, these genes were used to analyze effects of the ACs. A total of 14 major ACs were detected in the 326 cultivars (Fig. 3). There was a significant difference in the GI values among the AC classes. Compared to the AC14 with the high-GI alleles at the three genes, four ACs (AC1, AC2, AC5, and AC8) had significantly lower GIs in at least two environments and the values decreased by the ranges of 17.0% (AC8)-38.2% (AC1) in 2018, 13.2% (AC8)-40.3% (AC1) in 2019, and 9.9% (AC8)-28.9% (AC1) in 2020.

### Validation of effects of Hap1 and *TaSdr-B1a*

Based on the haplotypic analysis of *TaMFT*, 206 homozygous plants comprised of 113 Hap1 and 96 Hap4 were detected from the 495 plants in the Lunxuan 13 × Bainong 3217 F_2_ population. Fifty-six plants carried the homozygous *TaSdr-B1a* genotype and 53 plants carried the homozygous *TaSdr-B1b* genotype in *TaSdr-B1*. These F_2_ plants and derived F_3_ lines were evaluated for their GIs. To avoid confusion, the heterozygous plants or lines were excluded.

The GI values varied in 206 F_2_ plants (Fig. S4a) and F_3_ (Fig. S4b) lines. More than 90% F_2_ plants and F_3_ lines showed significantly lower GIs than the PHS-susceptible parent Lunxuan 13. Thirteen F_2_ plants and five F_3_ lines were not significantly different in GI values from the PHS-resistant parent Bainong 3217.

**Fig.4:**
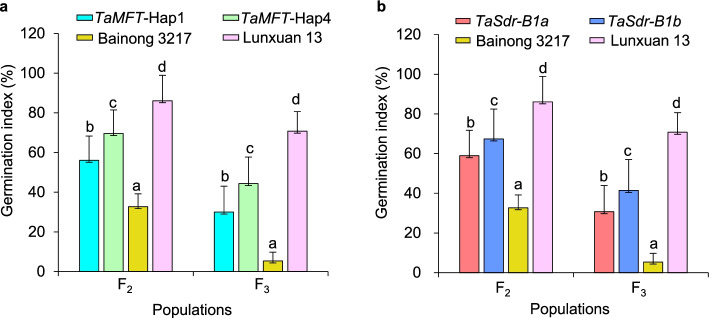
Comparison of germination index (GI) between two haplotypes of *TaMFT* (**a**) and contrasting alleles of *TaSdr-B1* (**b**) in the Lunxuan 13 × Bainong 3217 F_2_ and F_3_ populations. Different letters in F_2_ and F_3_ indicate significance of GI at *P* < 0.05, respectively

**Fig.5:**
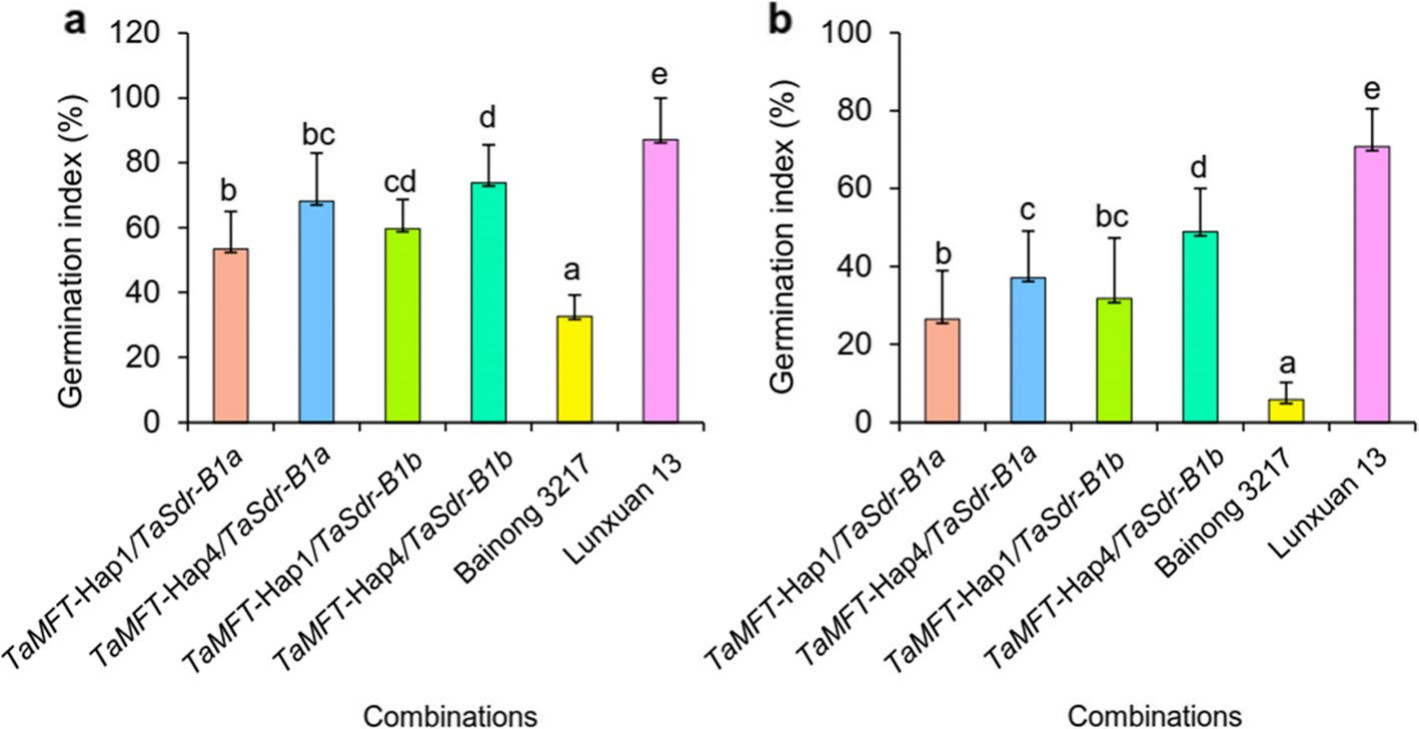
Comparison of germination index (GI) among allelic combinations of *TaMFT* and *TaSdr-B1* in the Lunxuan 13 × Bainong 3217 F_2_ (**a**) and F_3_ (**b**) populations. Different letters indicate significance of GI at *P* < 0.05

Significant phenotypic difference in GIs was found between the progenies with different *TaMFT* haplotypes and *TaSdr-B1* alleles in the Lunxuan 13 × Bainong 3217 population (Fig. 4). The Hap1 progenies reduced the GI values by 13.5% and 14.4% compared to those with the haplotype Hap4 in the F_2_ and F_3_ populations (Fig. 4a), respectively (*P* < 0.05). Consistently, the progenies with the low-GI allele *TaSdr-B1a* showed significantly lower GI values than those with the high-GI allele *TaSdr-B1b* (*P* < 0.05) (Fig. 4b). Compared to the PHS-susceptible parent Lunxuan 13, the progenies with the Hap1 and *TaSdr-B1a* reduced the GI values by 29.9% and 27.1% in the F_2_ population, respectively, and 40.8% and 40.0% in the F_3_ population, respectively (*P* < 0.05). The AC *TaMFT*-Hap1*/TaSdr-B1a* had the smallest GIs among the four ACs in the F_2_ (Fig. 5a) and F_3_ (Fig. 5b) populations. In comparison with Lunxuan 13, the progenies with the *TaMFT*-Hap1/*TaSdr-B1a* genotype reduced the GI values by 32.7% and 44.3% in the F_2_ and F_3_ populations, respectively (*P* < 0.05).

## Discussion

### Performance of PHS resistance in winter wheat cultivars

ANOVA and broad-sense heritability analysis suggested that the genetic variation of PHS resistance was mainly controlled by genotype, but year and genotype × year interaction also affected PHS resistance. The consistent finding was reported in the previous study [23]. Temperature is a major external determinant on seed dormancy during seed development in wheat [36]. High temperatures during seed development reduces the level of seed dormancy [19]. Late flowering delayed sampling and continuous high temperature (Figure S1) occurred at late maturity stage in 2019, which might result in reducing the depth of seed dormancy and making all wheat cultivars with higher GIs than other years. In all 10 genes identified, the Low-GI alleles/haplotype had better PHS resistance than the corresponding high-GI alleles/haplotype in each individual year (Table 2), suggesting that these Low-GI alleles/haplotype had stable effects on PHS resistance in different environment, and they can be used to improve wheat PHS in different breeding programs.

A wide variation in the GI values was observed in the winter wheat cultivars examined, but most of them were susceptible to PHS (Fig. S2a, Table S4). This is attributed to the fact that modern wheat cultivars have been domesticated by human in order to improve their adaptation and productivity, so they have relative uniform and rapid germination ability [37]. The PHS-resistant cultivars identified will be useful as donors for PHS improvement.

The red-grained cultivars showed usually more resistant to PHS than the white-grained ones [6, 15]. All the red-grained cultivars examined proved to carry at least one resistance allele from the *Tamyb10*-*A1*, *Tamyb10-B1*, and *Tamyb-D1* genes. Furthermore, these cultivars mainly adapted to the MLWZ and SWWZ where PHS occurs frequently during harvesting seasons due to wet weather conditions [4]. This might make wheat cultivars more tolerant to PHS in those wheat areas by natural and artificial selections.

### Comparison of PHS resistance of resistance allele, haplotype and allelic combination

Cultivars with the low-GI allele showed higher PHS resistance than those with the contrasting high-GI allele for each gene examined, which is in agreement with the previous studies [16, 18–22, 26–29]. However, the level of PHS resistance between the contrasting alleles at certain genes varied in different genetic backgrounds [38]. For example, a main-effect locus *TaMKK3-A* associated with PHS resistance was identified in different populations and explained 30–38% phenotypic variations [10]. The difference of GI between alleles *TaMKK3-Aa* and *TaMKK3-Ab* was 15.5% in the Tutoumai A/NW97S186//NW97S186 BC_2_ population [10], but the corresponding value was only 2.3%, 1.8% and 1.7% in 2018, 2019, and 2020, respectively (Table 2) in this study. This might be attributed to the impacts of genetic backgrounds.

It is noteworthy that the resistance allele *TaPHS1-222C* always present in the *TaMFT* haplotype Hap1, but absent in the other haplotypes. Wang et al. [39] also found that the *TaPHS1-222C* allele was consistently present with *TaPHS1-646G* and *TaPHS1-666A* alleles in haplotype GCA. Furthermore, there was no phenotypic difference in GI between the allele *TaPHS1-222C* and haplotype Hap1 in this study (data not shown). This suggests that the *TaPHS1-222C* marker is effective to select Hap1 genotypes in the process of MAS.

Among the 14 ACs, AC1 carrying Hap1 and *TaSdr-B1a* showed smaller GI than the other ACs (Fig. 3), and the pyramiding effect was further verified in the Lunxuan 13 × Bainong 3217 population (Fig. 5). Even though the Hap1 and *Tamyb10-D1b* alleles had the largest phenotypic effects on GIs at a single locus level, the effects of pyramiding two alleles are not clear because no cultivar carries both alleles in this study.

The known PHS resistance genes were not detected in the low-GI cultivars such as Yangmai 20 (11.6%), Lunan 11 (13.8%), and Luomai 4 (15.5%), demonstrating that these cultivars may carry new genes associated with the low GI. It warrants genetic analysis to dissect the QTL for their low GI performance.

### Distribution of the low-GI alleles and allelic combinations

The haplotype Hap1 with the largest effect on PHS resistance was detected only in 11 cultivars (Table S4), suggesting that it is a rare haplotype in modern wheat cultivars. The low frequencies of the Hap 1 were also reported in Chinese accessions (2.79%) [39] and landraces (2.0%) from the Fertile Crescent and surrounding areas [40], and wheat accessions from the USA (24.4%) [40]. Hence, it is necessary to introgress the Hap1 into PHS-susceptible cultivars by molecular marker of *TaPHS1-222C* allele due to tightly association between the *TaPHS1-222C* allele with the other low-GI alleles [39]. Another allele *Tamyb10-D1b* also showed low frequency (8.6%), and mainly present in the red-grained cultivars (Fig. 2). In China, white-grained cultivars have been preferentially selected than red-grained cultivars by wheat breeders [6], which might result in low frequency of this allele.

### Validation of effects of low-GI allele and haplotype on resistance to PHS

Lunxuan 13 is an elite high-yielding wheat cultivar, but susceptible to PHS [41]. We tried to introgress the low-GI Hap1 and allele *TaSdr-B1a* from the PHS-resistant parent Bainong 3217 to Lunxuan 13. The PHS resistance of the progenies were significantly enhanced. This indicates that they can efficiently improve PHS resistance of a susceptible cultivar. Pyramiding of Hap1 and *TaSdr-B1a* showed lower GIs than those carrying single haplotype or allele, suggesting that they have additive effects. Similar result was reported by analyzing the combining effects of *TaPHS1* and *TaMKK3-A* [42]. Even if Hap1 and *TaSdr-B1a* showed additive effects, there was significant difference in mean GI between the pyramiding progenies and PHS resistance parent Bainong 3217, suggesting that Bainong 3217 might carry other unknown PHS-resistant loci.

## Conclusions

The comparison of effects between contrasting alleles on GI in single gene combining haplotype analysis showed that the haplotype Hap1 of *TaMFT* gene had the best PHS resistance. This haplotype can be preferentially used to enhance PHS resistance due to its high effectiveness and low distribution frequency. Combining haplotype Hap1 and the *TaSdr-B1a* allele in AC1 exhibited additive effects on GIs in winter wheat cultivars and validated in genetic population. This study will facilitate the parental selection and MAS for wheat PHS resistance, and provide important materials for identifying new PHS-resistant genes.

## Supplementary Information

**Additional file1: Table S1.** Detailed information on the code number, name, grain color, pedigree, year of release, origin and wheat zone for the 326 winter wheat cultivars. **Table S2** Functional markers associated with pre-harvest sprouting resistance in wheat. **Table S3** Analysis of variance for germination index (GI) in the 326 winter wheat cultivars across three years. **Table S4** Allelic variations of 10 genes associated with pre-harvest sprouting resistance and germination index (GI) in the 326 winter wheat cultivars. **Fig. S1.** The daily average temperature (**a**), relative humidity (**b**) and rainfall (**c**) of the three cropping seasons during 2017–2018, 2018–2019, and 2019–2020. **Fig S2.** Distribution of germination index (GI) in the 326 wheat cultivars (**a**) and comparison of GI in different wheat zones (**b**), provinces (**c**) and grain colors (**d**). NWWZ, Northern Winter Wheat Zone; YHWZ, Yellow and Huai River Valleys Winter Wheat Zone; SWWZ, Southwestern Winter Wheat Zone; MLWZ, Middle and Lower Yangtze River Valleys Winter Wheat Zone. Different letters in individual year and mean value indicate significant differences of GI at *P* < 0.05. **Fig. S3.** Correlation analysis of germination index between years. * and **, significant at *P* < 0.05 and *P* < 0.01, respectively. **a** Correlation between 2018 and 2019; **b** Correlation between 2018 and 2020; **c** Correlation between 2019 and 2020. **Fig. S4.** Distribution of germination index in the Lunxuan 13 × Bainong 3217 F_2_ (**a**) and F_3_ (**b**) populations.

## Data Availability

All data generated or analyzed during this study are included in this published article.

## Abbreviations

AC: Allelic combination
CAPS: Cleaved amplified polymorphic sequence
GI: Germination index
KASP: Kompetitive allele-specific PCR
MAS: Marker-assisted selection
MLWZ: The middle and lower yangtze river valleys winter wheat zone
NWWZ: The northern winter wheat zone
PHS: Pre-harvest sprouting
QTL: Quantitative trait loci
STS: Sequence-tagged site
SWWZ: The southwestern winter wheat zone
YHWZ: The yellow and huai river valleys winter wheat zone
