# Supplementary material for: Identification of effective alleles and haplotypes conferring pre-harvest sprouting resistance in winter wheat cultivars
Source: BMC Plant Biol. 2022 Jul 6;22:326. doi: 10.1186/s12870-022-03710-w (PMC9258197; doi:10.1186/s12870-022-03710-w)
Supplement: Supplementary file 1 — Additional file1: Table S1. Detailed information on the code number, name, grain color, pedigree, year of release, origin and wheat zone for the 326 winter wheat cultivars. Table S2 Functional markers associated with pre-harvest sprouting resistance in wheat. Table S3 Analysis of variance for germination index (GI) in the 326 winter wheat cultivars across three years. Table S4 Allelic variations of 10 genes associated with pre-harvest sprouting resistance and germination index (GI) in the 326 winter wheat cultivars. Fig. S1. The daily average temperature (a), relative humidity (b) and rainfall (c) of the three cropping seasons during 2017–2018, 2018–2019, and 2019–2020. Fig S2. Distribution of germination index (GI) in the 326 wheat cultivars (a) and comparison of GI in different wheat zones (b), provinces (c) and grain colors (d). NWWZ, Northern Winter Wheat Zone; YHWZ, Yellow and Huai River Valleys Winter Wheat Zone; SWWZ, Southwestern Winter Wheat Zone; MLWZ, Middle and Lower Yangtze River Valleys Winter Wheat Zone. Different letters in individual year and mean value indicate significant differences of GI at P < 0.05. Fig. S3. Correlation analysis of germination index between years. * and **, significant at P < 0.05 and P < 0.01, respectively. a Correlation between 2018 and 2019; b Correlation between 2018 and 2020; c Correlation between 2019 and 2020. Fig. S4. Distribution of germination index in the Lunxuan 13 × Bainong 3217 F2 (a) and F3 (b) populations. [file 12870_2022_3710_MOESM1_ESM.doc]

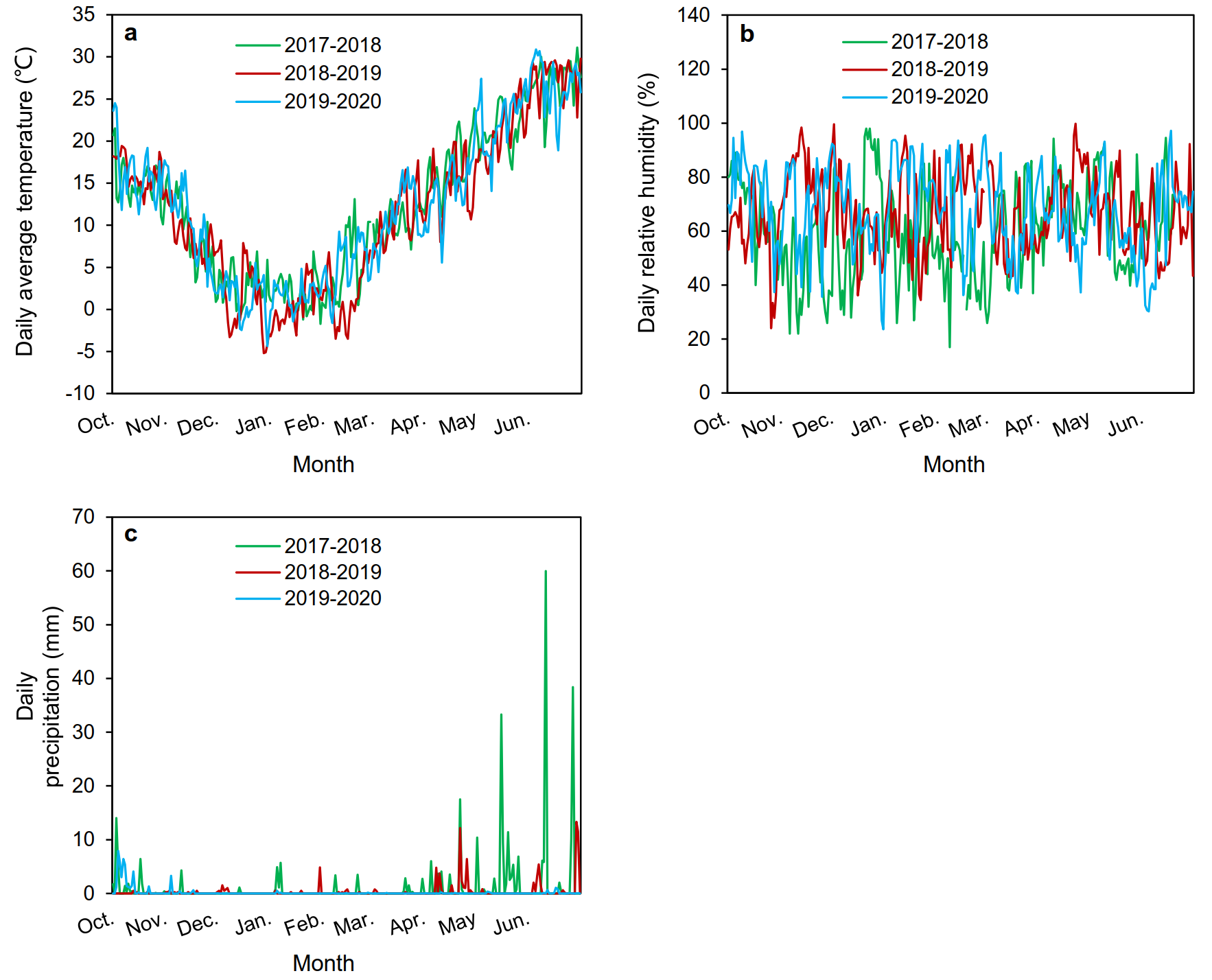


**Figure S1.** The daily average temperature (**a**), relative humidity (**b**) and precipitation (**c**) of the three cropping seasons during 2017-2018, 2018-2019, and 2019-2020.


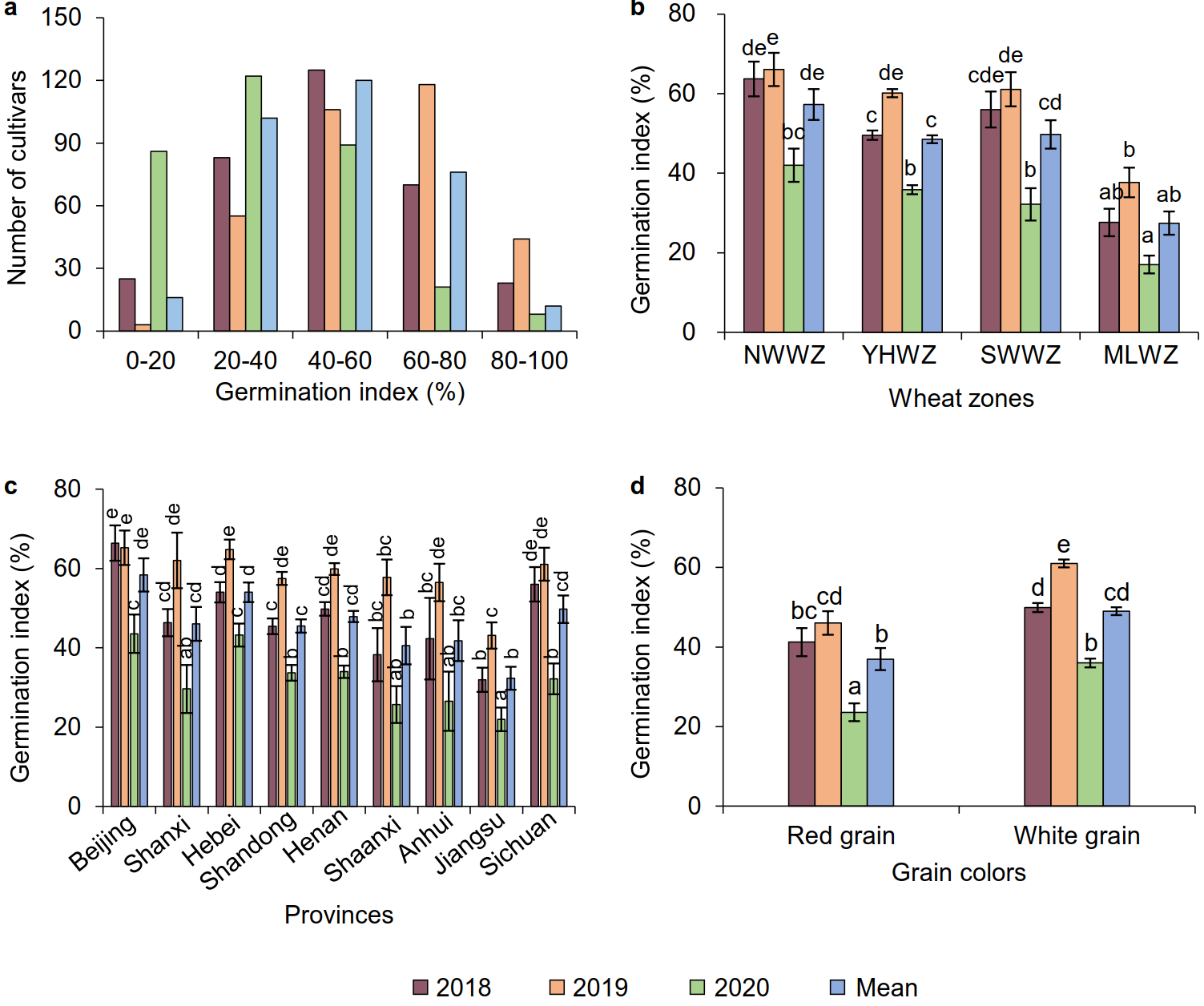


**Figure S2.** Distribution of germination index (GI) in the 326 wheat cultivars (**a**) and comparison of GI in different wheat zones (**b**), provinces (**c**) and grain colors (**d**). NWWZ, Northern Winter Wheat Zone; YHWZ, Yellow and Huai River Valleys Winter Wheat Zone; SWWZ, Southwestern Winter Wheat Zone; MLWZ, Middle and Lower Yangtze River Valleys Winter Wheat Zone. Different letters in individual year and mean value indicate significant differences of GI at *P* < 0.05.


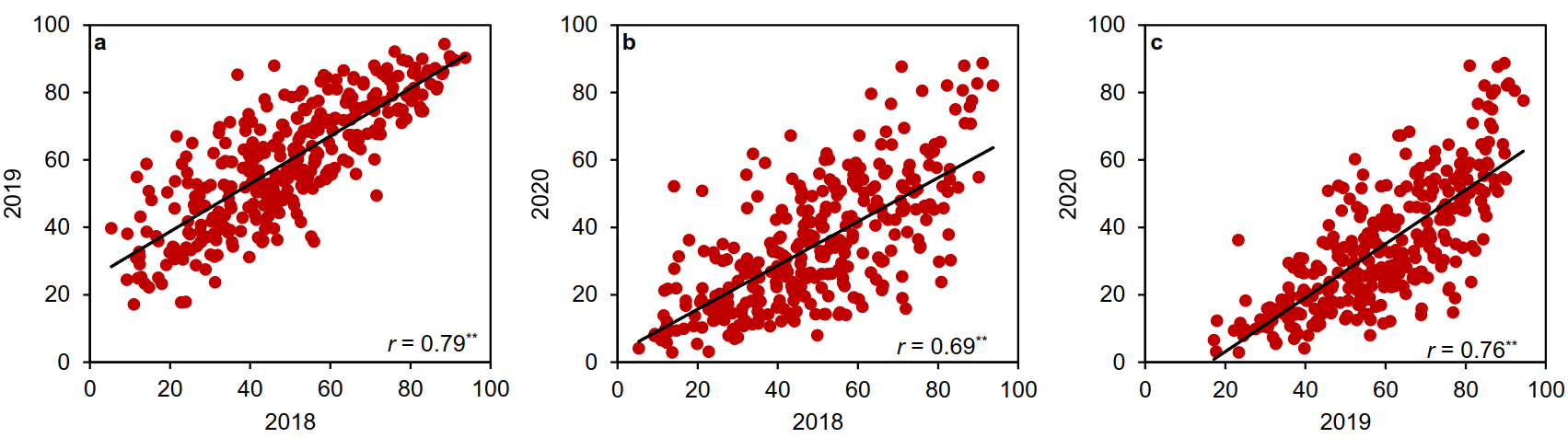


**Figure S3.** Correlation analysis of germination index between years. * and **, significant at *P* < 0.05 and *P* < 0.01, respectively. **a** Correlation between 2018 and 2019; **b** Correlation between 2018 and 2020; **c** Correlation between 2019 and 2020.


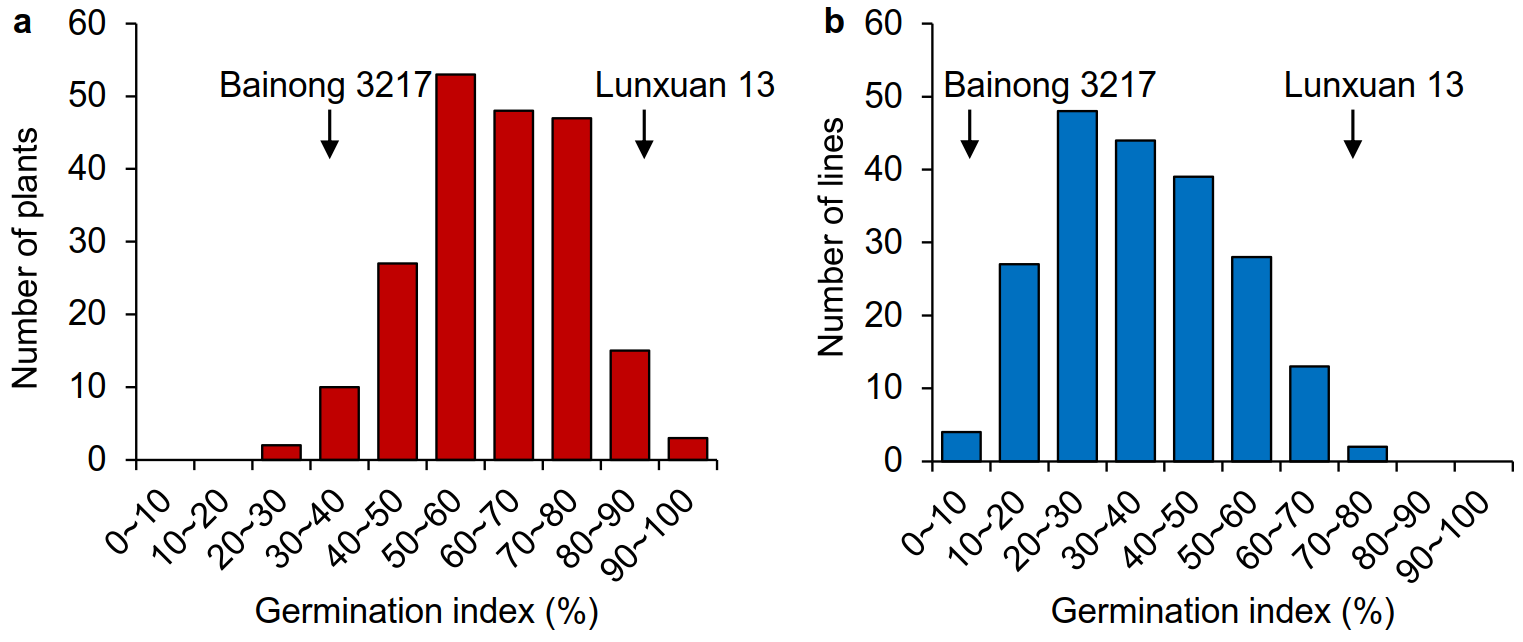


**Figure S4.** Distribution of germination index in the Lunxuan13 × Bainong 3217 F_2_ (**a**) and F_3_ (**b**) populations.

**Table S1 Detailed information on the code number, name, grain color, pedigree, year of release, origin and wheat zone for 326 Chinese winter wheat cultivars**

| Code number | Name | Grain color | Pedigree | Year of release | Origin | Wheat zone |
| --- | --- | --- | --- | --- | --- | --- |
| 1 | Jing 411 | white | Fengkang 2/Changfeng 1 | 1991 | Beijing | NWWZ |
| 2 | Jing 9428 | red | Jing 411/Yidunban | 1996 | Beijing | NWWZ |
| 3 | Jingdong 8 | red | (Afuleer/5238-016//Hong 4)/(You 7/Luo 10) | 1995 | Beijing | NWWZ |
| 4 | Jingdong 12 | red | Jingdong 23/Jingdong 17 | 2004 | Beijing | NWWZ |
| 5 | Jingdong 17 | white | Jingdong 8/RHT3//931 | 2006 | Beijing | NWWZ |
| 6 | Jingdong 22 | red | Taigu male sterile wheat recurrent selection | 2007 | Beijing | NWWZ |
| 7 | Jinghua 9 | red | Jingshi 467/Jingdong 8//Jingdan 93-219 | 2007 | Beijing | NWWZ |
| 8 | Jinmai 47 | white | 12057//Han 522/K37-30 | 1995 | Shanxi | NWWZ |
| 9 | Lunxuan 167 | red | Jingdong 8/Lunxuan 987 | 2014 | Beijing | NWWZ |
| 10 | Lunxuan 169 | red | Jingdong 8/Lunxuan 987 | 2014 | Beijing | NWWZ |
| 11 | Lunxuan 987 | red | Dwarf-male-sterile wheat recurrent selection population | 2003 | Beijing | NWWZ |
| 12 | Ningchun 4 | red | Suoruola 64/Hongtu | 1976 | Ningxia | NWWZ |
| 13 | Nongda 3432 | red | Nongda 9136/F 390 | 2005 | Beijing | NWWZ |
| 14 | Xindong 20 | white | 3286/Fu 79-220 | 1995 | Xinjiang | NWWZ |
| 15 | Xindong 22 | white | Nuosita/Huachun 84-1//79-4/Lovrin 13 | 1998 | Xinjiang | NWWZ |
| 16 | Zhongmai 8 | white | Hehua 971-3/JiZ 76 | 2009 | Tianjin | NWWZ |
| 17 | Zhongmai 9 | white | Siyang 936//(1523/1524)83jian 25 | 1997 | Beijing | NWWZ |
| 18 | Zhongmai 175 | white | BPM27/Jing 411 | 2007 | Beijing | NWWZ |
| 19 | Zhongmai 415 | white | Guinong 415/Jing 411//Jing 411 | 2010 | Beijing | NWWZ |
| 20 | Cangmai 6005 | red | Jiusan 32664 (96)/Jiusan3U117//Gelaini | 2010 | Hebei | YHWZ |
| 21 | Gao 2018 | white | 9411/98172 | 2005 | Hebei | YHWZ |
| 22 | Gaocheng 8901 | white | 7279/Xingfumai//Linzhangmai | 1998 | Hebei | YHWZ |
| 23 | Gaocheng 9415 | white | 8515/Annong 8455 | 2003 | Hebei | YHWZ |
| 24 | Gaoyou 503 | white | 78506/Zaoyou 504 | 2001 | Hebei | YHWZ |
| 25 | Gaoyou 9618 | white | 8515-4/8901-11-14 | 2005 | Hebei | YHWZ |
| 26 | Han 4564 | white | 88-6021/Shi 5144 | 2006 | Hebei | YHWZ |
| 27 | Han 4589 | white | Han 4032/85 zhong 47 | 2001 | Hebei | YHWZ |
| 28 | Han 5316 | white | Han 7808/CA 8059//85 zhong 47 | 2000 | Hebei | YHWZ |
| 29 | Han 6172 | white | 4032/Zhongyin 1 | 2003 | Hebei | YHWZ |
| 30 | Hanmai 11 | white | Han 87-1/Shi 5144 | 2007 | Hebei | YHWZ |
| 31 | Hanmai 13 | white | Shannongtai 91136/Jimai 36 | 2009 | Hebei | YHWZ |
| 32 | Hanmai 14 | white | Han 93-6182/Shannongtai 91136 | 2011 | Hebei | YHWZ |
| 33 | Hanmai 16 | white | Han 5267/Han 4564 | 2014 | Hebei | YHWZ |
| 34 | Hanmai 17 | white | Guan 35/Gao 9411 | 2016 | Hebei | YHWZ |
| 35 | Heng 4399 | white | Han 6172/Heng 28 | 2003 | Hebei | YHWZ |
| 36 | Heng 5229 | white | Ji 5418/Heng 5041 | 2004 | Hebei | YHWZ |
| 37 | Heng 6632 | white | Heixiaomai/Heng 8116 | 2013 | Hebei | YHWZ |
| 38 | Heng 7228 | white | Ji 5418/Heng 5041 | 2005 | Hebei | YHWZ |
| 39 | Hengguan 35 | white | 84Guan 749/Heng 87-4263 | 2004 | Hebei | YHWZ |
| 40 | Henong 326 | white | 84(252)/86(298) | 1997 | Hebei | YHWZ |
| 41 | Henong 6049 | white | Shi 6021/Henong 9145 | 2009 | Hebei | YHWZ |
| 42 | Henong 822 | white | 88S522/92(98) | 2004 | Hebei | YHWZ |
| 43 | Henong 972 | white | Taigu male sterile wheat recurrent selection | 1997 | Hebei | YHWZ |
| 44 | Ji 5265 | white | Ji 5006/9204 | 2009 | Hebei | YHWZ |
| 45 | Jimai 26 | white | Aiganzao/Lovrin 10/Jinfeng 1 | 1988 | Hebei | YHWZ |
| 46 | Jimai 30 | white | 78-3147/Shi 4414 | 2000 | Hebei | YHWZ |
| 47 | Jimai 325 | white | JI5157/Shi02-7221 | 2016 | Hebei | YHWZ |
| 48 | Jimai 36 | white | [(715017/Shanqian)/75-78]F_4_/9435 | 1992 | Hebei | YHWZ |
| 49 | Jimai 38 | white | Zhi 4001/Shi 4212-10 | 1998 | Hebei | YHWZ |
| 50 | Jimai 418 | white | Ji 5157/Shi 20-7221 | 2016 | Hebei | YHWZ |
| 51 | Jimai 42 | white | Jingshuang 9/Baofeng 7228 | 1997 | Hebei | YHWZ |
| 52 | Jimai 585 | white | Taigu male sterile wheat recurrent selection | 2011 | Hebei | YHWZ |
| 53 | Jinhe 9123 | white | Shi 4185/92R137 | 2008 | Hebei | YHWZ |
| 54 | Kenong 199 | white | Shi 4185/Kenong 9204 | 2006 | Hebei | YHWZ |
| 55 | Kenong 2009 | white | [(1BL-1RS148/BE-1)/Kenong1095]/Kenong1095 | 2015 | Hebei | YHWZ |
| 56 | Lunxuan 061 | white | Dwarf-male sterile wheat recurrent selection population | 2009 | Hebei | YHWZ |
| 57 | Lunxuan 103 | white | Shimai 12/Shijiazhuang 8 | 2015 | Hebei | YHWZ |
| 58 | Lunxuan 145 | white | Jimai22/169-5Z | 2020 | Hebei | YHWZ |
| 59 | Shi 4185 | white | Taigu male sterile wheat recurrent selection | 1997 | Hebei | YHWZ |
| 60 | Shi 4366 | white | Liangxing 99/Shiyou 17 | 2015 | Hebei | YHWZ |
| 61 | Shijiazhuang 8 | white | Shi 91-5065/Jimai 38 | 2001 | Hebei | YHWZ |
| 62 | Shiluan 02-1 | white | 9411/9430 | 2007 | Hebei | YHWZ |
| 63 | Shimai 12 | white | Shi91-5096/Jimai 23 | 2004 | Hebei | YHWZ |
| 64 | Shimai 14 | white | Shi 4185/92R137 | 2004 | Hebei | YHWZ |
| 65 | Shimai 15 | white | Jimai 38/92R137 | 2005 | Hebei | YHWZ |
| 66 | Shimai 18 | white | (92Jian 3/T447)F_2_/Jimai 38//Shi 41853 | 2008 | Hebei | YHWZ |
| 67 | Shimai 19 | white | Shi 4185/(Yanfu 188/Lin 8014) | 2009 | Hebei | YHWZ |
| 68 | Shimai 22 | white | Shi 4185/(Yanfu 188/Lin 8014) | 2013 | Hebei | YHWZ |
| 69 | Shinong 086 | white | Lumai 14/Han 6172 | 2014 | Hebei | YHWZ |
| 70 | Shixin 733 | white | Damuzhiai/Shixin 163 | 2001 | Hebei | YHWZ |
| 71 | Shixin 828 | white | 422/Shixin 163//612 | 2013 | Hebei | YHWZ |
| 72 | Shiyou 20 | white | Ji935-352/Jinan 17 | 2011 | Hebei | YHWZ |
| 73 | XiaoYan 81 | white | Xiaoyan 54/8602 | 2005 | Hebei | YHWZ |
| 74 | Xingmai 13 | white | Heng 9117-2 /Han 4589 | 2016 | Hebei | YHWZ |
| 75 | Xingmai 4 | white | KeyiNC 20/4564//Gaoyou 503 | 2007 | Hebei | YHWZ |
| 76 | Xingmai 6 | white | Jimai 36/Han 612 | 2008 | Hebei | YHWZ |
| 77 | Yingbo 700 | white | Taigu male sterile wheat/Ji 93-5031 | 2012 | Hebei | YHWZ |
| 78 | Zhongmai 155 | white | Jimai 19/Lumai 21 | 2003 | Hebei | YHWZ |
| 79 | Zhongxinmai 9 | white | D703/Han 4589 | 2015 | Hebei | YHWZ |
| 80 | Jimai 19 | white | Lumai 13/Linfen 5064 | 2001 | Shandong | YHWZ |
| 81 | Jimai 20 | white | Lumai 14/884187 | 2003 | Shandong | YHWZ |
| 82 | Jimai 21 | white | 965186/Chuannongda 84-1109//Ji 84-5418 | 2004 | Shandong | YHWZ |
| 83 | Jimai 22 | white | 935024/935106 | 2006 | Shandong | YHWZ |
| 84 | Jinan 13 | white | White Orofen//Huixianhong/Abbondanza | 1980 | Shandong | YHWZ |
| 85 | Jinan 16 | white | Taigu male sterile line/Shannongfu 63//775-1 | 1998 | Shandong | YHWZ |
| 86 | Jinan 17 | white | Linfen 5064/Lumai 13 | 1999 | Shandong | YHWZ |
| 87 | Liangxing 66 | white | Ji 91102/Ji 935031 | 2008 | Shandong | YHWZ |
| 88 | Liangxing 99 | white | (Ji 91102/Lumai 14)/PH85-16 | 2004 | Shandong | YHWZ |
| 89 | Linmai 4 | white | Lumai 23/Lin9015 | 2006 | Shandong | YHWZ |
| 90 | Lukenmai 9 | white | Xu 9935/Yanyou 361 | 2010 | Shandong | YHWZ |
| 91 | Lumai 1 | white | Aifeng 3/(Mengxian 201/Niuzhute)F_1_ | 1980 | Shandong | YHWZ |
| 92 | Lumai 13 | white | [Lovrin 13/71(17)6-1]74(11)1-1-3/Laiyang 584 | 1979 | Shandong | YHWZ |
| 93 | Lumai 14 | white | C149/F4530 | 1992 | Shandong | YHWZ |
| 94 | Lumai 15 | white | TaiYangmai 1-B1/757318//104-14 | 1996 | Shandong | YHWZ |
| 95 | Lumai 21 | white | Lumai 13/Yumai 2 | 1996 | Shandong | YHWZ |
| 96 | Lumai 22 | white | (Taishan 2/Yannong 15)F_1_/Jinghua 1 | 1996 | Shandong | YHWZ |
| 97 | Lumai 23 | white | Lumai 8/Laixiaomai | 1996 | Shandong | YHWZ |
| 98 | Lumai 7 | white | Lovrin 10/3/Weier/Youluo//Quxuan 26 | 1985 | Shandong | YHWZ |
| 99 | Luyuan 502 | white | 9940168/Jimai 19 | 2011 | Shandong | YHWZ |
| 100 | Qimai 2 | white | Weimai 8/05-38 | 2017 | Shandong | YHWZ |
| 101 | Qingfeng 1 | white | Lumai 14/Yannong 15 | 2006 | Shandong | YHWZ |
| 102 | Qingnong 2 | white | Lumai 14/Yannong 15//Aiganmai | 2010 | Shandong | YHWZ |
| 103 | Runong 14 | white | 84139//9215/876161 | 2010 | Shandong | YHWZ |
| 104 | Shannong 17 | white | L156/Laizhou 137 | 2009 | Shandong | YHWZ |
| 105 | Shannong 19 | white | (83(3)-113/1604)F_3_//886059 | 2010 | Shandong | YHWZ |
| 106 | Shannong 20 | white | PH 82-2-2/954072 | 2010 | Shandong | YHWZ |
| 107 | Shannong 22 | white | Taigu male sterile wheat recurrent selection | 2011 | Shandong | YHWZ |
| 108 | Shannong 24 | white | Taigu male sterile wheat recurrent selection | 2013 | Shandong | YHWZ |
| 109 | Shannong 28 | white | 4142/6125 | 2017 | Shandong | YHWZ |
| 110 | Shannong 30 | white | Tainong 18/Linmai 6 | 2017 | Shandong | YHWZ |
| 111 | Shannong 32 | white | 6125/954(5)-4 | 2016 | Shandong | YHWZ |
| 112 | Shannongfu 63 | white | Qubao/Orofen | 1982 | Shandong | YHWZ |
| 113 | Tainong 18 | white | Laizhou 137/369-7 | 2008 | Shandong | YHWZ |
| 114 | Taishan 1 | white | Bima 4/Zaoshu 1//Orofen | 1979 | Shandong | YHWZ |
| 115 | Taishan 21 | white | ((26744/Taishan 10)/Lumai 7)/Lumai 18 | 2003 | Shandong | YHWZ |
| 116 | Taishan 23 | white | 876161/881414 | 2004 | Shandong | YHWZ |
| 117 | Weimai 8 | white | 88-3149/Aus 621108 | 2003 | Shandong | YHWZ |
| 118 | Xinmai 296 | white | 935031/Lumai 23 | 2014 | Shandong | YHWZ |
| 119 | Yanfu 188 | white | Yanzhong 22/Xingmai 7721/Lumai 7 | 2002 | Shandong | YHWZ |
| 120 | Yanmai 98 | white | Jining 13/942 | 2010 | Shandong | YHWZ |
| 121 | Yannong 15 | white | Youbaomai/ST 2422/466 | 1982 | Shandong | YHWZ |
| 122 | Yannong 19 | white | Yan 1933/Shan 82-29 | 2001 | Shandong | YHWZ |
| 123 | Yannong 21 | white | Yan 1933/Shan 82-29 | 2002 | Shandong | YHWZ |
| 124 | Yannong 24 | white | Shan 229/Anmai 1 | 2004 | Shandong | YHWZ |
| 125 | Yannong 5286 | white | Lumai 14/945015 | 2007 | Shandong | YHWZ |
| 126 | Yannong 836 | white | Yan 9292 pedigree selection | 2013 | Shandong | YHWZ |
| 127 | Yannong 999 | white | Yanhangxuan 2/Lin 9511//YanBLU14-15 | 2016 | Shandong | YHWZ |
| 128 | Zhouyuan 9369 | white | PH 82-2-2/866-34 | 2007 | Shandong | YHWZ |
| 129 | Zimai 12 | white | 917065/910292 | 2001 | Shandong | YHWZ |
| 130 | Shunmai 1718 | white | 32S/Gabo | 2011 | Shanxi | YHWZ |
| 131 | Yaomai 16 | white | Jinmai 54/Beinong 8 | 2011 | Shanxi | YHWZ |
| 132 | Yunhan 618 | white | Yunhan 92-18/Xinchun 9 | 2010 | Shanxi | YHWZ |
| 133 | 04Zhong 36 | white | Bainong 64/Zhoumai 11 | 2006 | Henan | YHWZ |
| 134 | Aikang 58 | white | Zhoumai 11/Wenmai 6//Zhengzhou 8960 | 2005 | Henan | YHWZ |
| 135 | Bainong 160 | white | Duokang 893/Wenmai 6//Bainong 64/Wenmai 6 | 2007 | Henan | YHWZ |
| 136 | Bainong 207 | white | Duokang 893/Wenmai 6//Bainong 64/Wenmai 6 | 2013 | Henan | YHWZ |
| 137 | Bainong 3217 | white | Funo/Neixiang 5//Xiannong 39///Xinong 64/Yanda 24 | 1990 | Henan | YHWZ |
| 138 | Bainong 418 | white | Zhoumai 18/Aikang 58//Aikang 58 | 2015 | Henan | YHWZ |
| 139 | Baiquan 3039 | white | 65(14)3 pedigree selection | 1983 | Henan | YHWZ |
| 140 | Bonong 7023 | white | Funo pedigree selection | 1981 | Henan | YHWZ |
| 141 | Fanmai 5 | white | Ji 5418/Jingfan 309//Zhoumai 13 | 2005 | Henan | YHWZ |
| 142 | Fanmai 8 | white | Fanai 2/Yuanfan 3 | 2008 | Henan | YHWZ |
| 143 | Fengdecunmai 1 | white | Zhou 9811/Aikang 58 | 2011 | Henan | YHWZ |
| 144 | Fengwu 981 | white | Aizao 781/80(6)-3-3-10 | 2004 | Henan | YHWZ |
| 145 | Fumai 2008 | white | Aizao 781/80(6)-3-3-10 | 2006 | Henan | YHWZ |
| 146 | Guanmai 1 | white | Zhoumai 13/Bainong 64 | 2016 | Henan | YHWZ |
| 147 | Huaichuan 916 | white | Yumai 47/Xiaoyan 54 | 2011 | Henan | YHWZ |
| 148 | Huapei 3 | white | Hua 953350-1-2/Hua 962437-1-1 | 2006 | Henan | YHWZ |
| 149 | Huapei 5 | white | Yumai 18/Hua 4-3 | 2006 | Henan | YHWZ |
| 150 | Huapei 8 | white | 9824H-1-2/Zhengzhou 91138//Zhenzhou 91138 | 2009 | Henan | YHWZ |
| 151 | Junmai 99-7 | white | 98264/Yumai 52 | 2009 | Henan | YHWZ |
| 152 | Kaimai 18 | white | Kaimai 64/89zhong170//Kaimai 1003/Wen 2540 | 2006 | Henan | YHWZ |
| 153 | Kaimai 20 | white | Aikai 79/Kaimai 14 | 2011 | Henan | YHWZ |
| 154 | Kaimai 21 | white | Lu D 9401/Kaimai 16 | 2011 | Henan | YHWZ |
| 155 | Lankao 198 | white |  | 2011 | Henan | YHWZ |
| 156 | Luohan 11 | white | Yumai 25/Shannong 45 | 2008 | Henan | YHWZ |
| 157 | Luohan 13 | white | Luohan 2/Jinmai 47 | 2009 | Henan | YHWZ |
| 158 | Luohan 2 | white | Luoyang 77(111)/Jinmai 33 | 2001 | Henan | YHWZ |
| 159 | Luohan 6 | white | Yumai 49/Shannong 45 | 2006 | Henan | YHWZ |
| 160 | Luohan 7 | white | Yumai 41/Shannong 45 | 2007 | Henan | YHWZ |
| 161 | Luomai 18 | white | 4336/Zhoumai 16 | 2012 | Henan | YHWZ |
| 162 | Luomai 21 | white | Luomai 2/Zhoumai 13 | 2009 | Henan | YHWZ |
| 163 | Luomai 22 | white | Zhoumai 13/Yumai 49 | 2008 | Henan | YHWZ |
| 164 | Luomai 23 | white | Yumai 18/Huaiyin 9628 | 2009 | Henan | YHWZ |
| 165 | Luomai 24 | white | Luotai 911/huaimai 18 | 2013 | Henan | YHWZ |
| 166 | Luomai 26 | white | Kaimai 18/Aikang 58 | 2014 | Henan | YHWZ |
| 167 | Luomai 4 | white | 80(6)-3-3-10/Aizao 781 | 2003 | Henan | YHWZ |
| 168 | Luomai 8 | white | Yanzhong 1604/Wenmai 4 | 2007 | Henan | YHWZ |
| 169 | Lunxuan 13 | white | Zhoumai 16/Shimai 12 | 2018 | Henan | YHWZ |
| 170 | Pingan 6 | white | Laizhou 953/Wen 2540 | 2006 | Henan | YHWZ |
| 171 | Pingan 8 | white | Yumai 2/Zhoumai 13 | 2012 | Henan | YHWZ |
| 172 | Pingmai 998 | white | Shan 225/Zhoumai 9 | 2008 | Henan | YHWZ |
| 173 | Pumai 9 | white | Xuzhou 174/Neixiang 183//Yumai 24 | 2004 | Henan | YHWZ |
| 174 | Ruzhou 0319 | white | Taigu male sterile wheat recurrent selection | 2009 | Henan | YHWZ |
| 175 | Taikong 6 | white | Yummai 49 variant | 2003 | Henan | YHWZ |
| 176 | Taixue 7 | white | Yumai 57/Zhoumai 16 | 2011 | Henan | YHWZ |
| 177 | Tianmin 198 | white | R 81/Bainong 64//Yanzhan 4110 | 2014 | Henan | YHWZ |
| 178 | Wen 9519 | white | Lankao 4/Wen 2540 | 2004 | Henan | YHWZ |
| 179 | Wen 9629 | white | Yumai 21/Yumai 41 | 2004 | Henan | YHWZ |
| 180 | Wenmai 19 | white | Lankao 4/Wen 2540 | 2004 | Henan | YHWZ |
| 181 | Xinkemai 169 | white | Xinmai 18/Aikang 58 | 2018 | Henan | YHWZ |
| 182 | Xinmai 11 | white | Zhou 8826/Xinxiang 8577 | 2001 | Henan | YHWZ |
| 183 | Xinmai 18 | white | (C5/Xinxiang 3577)F_3_d1/Xinmai 9 | 2004 | Henan | YHWZ |
| 184 | Xinmai 19 | white | (C5/3577)F_3_/Xinmai 9 | 2006 | Henan | YHWZ |
| 185 | Xinmai 20 | white | Yanzhan 1/Wenmai 6 | 2007 | Henan | YHWZ |
| 186 | Xinmai 21 | white | Yanzhan 1/Xinmai 9 | 2009 | Henan | YHWZ |
| 187 | Xinmai 26 | white | Jinan 17/Xin 9408E1 | 2010 | Henan | YHWZ |
| 188 | Xinmai 29 | white | Yanzhan 4110/Zhoumai 16 | 2017 | Henan | YHWZ |
| 189 | Xinmai 30 | white | Xinmai 11/Zhoumai 16 | 2014 | Henan | YHWZ |
| 190 | Xinmai 9 | white | Yanzhan 1/Wenmai 6 | 1997 | Henan | YHWZ |
| 191 | Xinyuan 958 | white | Yumai 9/Yumai 34 | 2005 | Henan | YHWZ |
| 192 | Xuke 1 | white | 97-042/Luomai 4 | 2009 | Henan | YHWZ |
| 193 | Xuke 316 | white | Zhoumai 16/Bainong 64 | 2011 | Henan | YHWZ |
| 194 | Xuke 718 | white | Zhoumai 13/Luomai 4 | 2012 | Henan | YHWZ |
| 195 | Xunong 5 | white | Zhou 8846/Zhoumai 9 | 2005 | Henan | YHWZ |
| 196 | Yangao 1 | white | (806/B16)F_3_//Luoyang 7602 | 2002 | Henan | YHWZ |
| 197 | Yangao 21 | white | (Zhoumai 16/Yumai 49)F_2_//Yumai 18 | 2017 | Henan | YHWZ |
| 198 | Yanzhan 1 | white | (C39/78(6)9-2//Jimai 5418)F_3_/Yumai 18 | 2003 | Henan | YHWZ |
| 199 | Yanzhan 4110 | white | (C39/Xibei 78(6)9-2)/(FR81-3/Aizao 781-4)//Aizao 781-4 | 2003 | Henan | YHWZ |
| 200 | Yimai 6 | white | Zhoumai 9 pedigree selection/Yumai 18 | 2011 | Henan | YHWZ |
| 201 | Yubao 1 | white | Yumai 2/Zhou 8826 | 2008 | Henan | YHWZ |
| 202 | Yujiao 5 | white | Zheng 91138/Yumai 49 | 2011 | Henan | YHWZ |
| 203 | Yumai 10 | white | Zhongyin 4/Yanshi 4 | 1983 | Henan | YHWZ |
| 204 | Yumai 13 | white | Bainong 3217 pedigree selection | 1989 | Henan | YHWZ |
| 205 | Yumai 18 | white | Zhengzhou 761/Yanshi 4 | 1990 | Henan | YHWZ |
| 206 | Yumai 2 | white | 65(14)3/Kangxiuhuijuhong | 1983 | Henan | YHWZ |
| 207 | Yumai 21 | white | (Bainong 791/Yumai 2)/(Lu 1/Yanshi 4) | 1990 | Henan | YHWZ |
| 208 | Yumai 29 | white | 78(6)9-2/80(6)5-6-10 | 1993 | Henan | YHWZ |
| 209 | Yumai 34 | white | Lumai 1/Yumai 2 | 1994 | Henan | YHWZ |
| 210 | Yumai 38 | white | Luoyang 7602/7110 | 1995 | Henan | YHWZ |
| 211 | Yumai 416 | white | Yunong 49//Yumai 21/Yumai 35 | 2009 | Henan | YHWZ |
| 212 | Yumai 47 | white | Yumai 2/Baiquan 3139 | 1997 | Henan | YHWZ |
| 213 | Yumai 49 | white | Weng 2540 pedigree selection | 2000 | Henan | YHWZ |
| 214 | Yumai 49-198 | white | Yumai 49 pedigree selection | 2005 | Henan | YHWZ |
| 215 | Yumai 52 | white | Yumai 1/(Sicuandugan/Puyang 5) | 1998 | Henan | YHWZ |
| 216 | Yumai 54 | white | Bainong 8717/[(Yanda72-629-52/Shi82-5594)F_1_/Bainong 84-4046-1]F_2_ | 1998 | Henan | YHWZ |
| 217 | Yumai 58 | white | 394A/Siyang 188 | 1999 | Henan | YHWZ |
| 218 | Yumai 7 | white | Shanqianmai/Yanshi 4 | 1985 | Henan | YHWZ |
| 219 | Yumai 70 | white | (Mianyang 84-27/Neixiang 82C6)//Yumai 17 | 2000 | Henan | YHWZ |
| 220 | Yumai 70-36 | white | Neixiang 188 pedigree selection | 2004 | Henan | YHWZ |
| 221 | Yunong 202 | white | Yunong 27/Yunong 127 | 2007 | Henan | YHWZ |
| 222 | Yunong 949 | white | Zhengtaiyu 92215/90m434//90(232) | 2005 | Henan | YHWZ |
| 223 | Yunong 982 | white | HY 9153/Bainong 3217//Yumai 49 | 2009 | Henan | YHWZ |
| 224 | Zhengmai 004 | white | Yumai 13/90M434//Shi 89-6021 | 1998 | Henan | YHWZ |
| 225 | Zhengmai 366 | white | Yumai 47/PH 82-2-2 | 2005 | Henan | YHWZ |
| 226 | Zhengmai 379 | white | Zhoumai 13 /D9054-6 | 2016 | Henan | YHWZ |
| 227 | Zhengmai 7698 | white | Zhengmai 9405/4B269//Zhoumai 16 | 2011 | Henan | YHWZ |
| 228 | Zhengmai 9023 | white | (Xiaoyan 6/Xinogn 65//83-233/84-43)F_3_/3/Shan 213 | 2001 | Henan | YHWZ |
| 229 | Zhengmai 9962 | white | Ta 971832/Yumai 18 | 2009 | Henan | YHWZ |
| 230 | Zhengnong 16 | white | Zhengnong 7/Xiaoyan 6 | 2003 | Henan | YHWZ |
| 231 | Zhengnong 17 | white | Zhengnong 7/Xiaoyan 6 | 2003 | Henan | YHWZ |
| 232 | Zhengyumai 9987 | white | Yumai 21/Yumai 2//Yumai 57 | 2007 | Henan | YHWZ |
| 233 | Zhengzhou 3 | white | Fulanni/(Xinong 6028/Tainong 153) | 1965 | Henan | YHWZ |
| 234 | Zhengzhou 761 | white | 65(14)_1_//St2422/464/Zhengzhou 17 | 1976 | Henan | YHWZ |
| 235 | Zhongmai 1 | white | Luomai 4/97-26 pedigree selection | 2004 | Henan | YHWZ |
| 236 | Zhongmai 2 | white | 73-5272/Duofunuojiya//AUS///Punong3665-2 | 2007 | Henan | YHWZ |
| 237 | Zhongmai 875 | white | Zhoumai 16/Liken 4 | 2014 | Henan | YHWZ |
| 238 | Zhongmai 895 | white | Zhoumai 16/Liken 4 | 2012 | Henan | YHWZ |
| 239 | Zhongmai 998 | white | (Bainong 71-22/Yumai 18)//(Wen 2540/Lovrin 10) | 2008 | Henan | YHWZ |
| 240 | Zhongyu 10 | white | Yumai 41/Baofeng 94-24 | 2007 | Henan | YHWZ |
| 241 | Zhongyu 12 | white | Dwarf-male-sterile wheat/Bainong 64 | 2008 | Henan | YHWZ |
| 242 | Zhongyu 6 | white | Zhongyu 3/Lumai 14 | 2001 | Henan | YHWZ |
| 243 | Zhongyu 9 | white | Yumai 21/92R139 | 2004 | Henan | YHWZ |
| 244 | Zhongyu 9398 | white | Dwarf-male-sterile wheat/Xinmai 18 | 2012 | Henan | YHWZ |
| 245 | Zhoumai 11 | white | Zhou 8425B/Yumai 17 | 1998 | Henan | YHWZ |
| 246 | Zhoumai 16 | white | Zhou 9/Zhou 8425B | 2002 | Henan | YHWZ |
| 247 | Zhoumai 17 | white | Aizao 781/Zhou 8425B//Zhoumai 9 | 2004 | Henan | YHWZ |
| 248 | Zhoumai 18 | white | Neixiang 185/Zhoumai 9 | 2004 | Henan | YHWZ |
| 249 | Zhoumai 19 | white | Naixiang 185/Zhoumai 9 | 2008 | Henan | YHWZ |
| 250 | Zhoumai 20 | white | Zhou 9/Zhou 8425B | 2006 | Henan | YHWZ |
| 251 | Zhoumai 22 | white | Zhoumai 12/Wenmai 6//Zhoumai 13 | 2007 | Henan | YHWZ |
| 252 | Zhoumai 23 | white | Zhoumai 13/Xinmai 9 | 2008 | Henan | YHWZ |
| 253 | Zhoumai 24 | white | Zhoumai 16/Shanyou 225 | 2009 | Henan | YHWZ |
| 254 | Zhoumai 25 | white | La 95021/Zhoumai 12 | 2011 | Henan | YHWZ |
| 255 | Zhoumai 26 | white | Zhoumai 24/Zhoumai 22 | 2012 | Henan | YHWZ |
| 256 | Zhoumai 27 | white | Zhoumai 16/Aikang 58 | 2011 | Henan | YHWZ |
| 257 | Zhoumai 28 | white | Zhoumai 18/Zhoumai 22//Zhou 2168 | 2014 | Henan | YHWZ |
| 258 | Zhoumai 30 | white | Zhoumai 23/Zhoumai 18-15 | 2016 | Henan | YHWZ |
| 259 | Zhoumai 32 | white | Aikang 58/Zhoumai 24 | 2014 | Henan | YHWZ |
| 260 | Zhumai 4 | white | 89 zhong 170/Bian 8539-2 | 2004 | Henan | YHWZ |
| 261 | Aifeng 3 | white | Xiannong 39/58 (18) 2//Fengchan 3 | 1971 | Shaanxi | YHWZ |
| 262 | Fengchan 3 | white | Danmai 1/Xinong 6028 | 1964 | Shaanxi | YHWZ |
| 263 | Jinfeng 3 | white | Yumai 25/Danxuan912//Ta1C7 sterile wheat recurrent selection | 2005 | Shanxi | YHWZ |
| 264 | Shan 229 | white | Shan 7853//TB902/Xiaoyan 6 | 1993 | Shaanxi | YHWZ |
| 265 | Xiaan 8 | white | St1 472/506//Aiganzao | 1978 | Shaanxi | YHWZ |
| 266 | Xiaoyan 22 | white | Xiaoyan 6/775-1//Xiaoyan 107 | 1998 | Shaanxi | YHWZ |
| 267 | Xiaoyan 54 | white | Xiaoyan 6 pedigree selection | 2000 | Shaanxi | YHWZ |
| 268 | XiaoYan 6 | white | (ST 2422/464)/Xiaoyan 96 | 1981 | Shaanxi | YHWZ |
| 269 | Xinong 219 | white | Puyou 9 /Q0031-21//Xinong 928 | 2013 | Shaanxi | YHWZ |
| 270 | Xinong 529 | white | Mianyang 26/Xiaoyan 597 | 2017 | Shaanxi | YHWZ |
| 271 | Xinong 979 | white | Xinong 2611//918/95 | 2005 | Shaanxi | YHWZ |
| 272 | Wanmai 19 | white | Boai 7422/Yumai 2 | 1997 | Anhui | YHWZ |
| 273 | Wanmai 38 | white | Yanzhong 114/85-15-9 | 1997 | Anhui | YHWZ |
| 274 | Wanmai 50 | white | Yumai 29/Wanmai 19 | 2005 | Anhui | YHWZ |
| 275 | Wanmai 52 | white | Zhengzhou 8329/Wanmai 19 | 2007 | Anhui | YHWZ |
| 276 | Wanmai 53 | white | Yumai 29/Wanmai 19 | 2005 | Anhui | YHWZ |
| 277 | Baomai 10 | white | Shi 4185/96Y6 | 2008 | Jiangsu | YHWZ |
| 278 | Baomai 2 | white | Baofeng 3-2-16/Yanfu 188 | 2012 | Jiangsu | YHWZ |
| 279 | Huaimai 18 | white | Yumai 13/Lumai 14 | 2001 | Jiangsu | YHWZ |
| 280 | Huaimai 20 | white | Yumai 13/Lumai 14 | 2003 | Jiangsu | YHWZ |
| 281 | Huaimai 21 | white | Huaimai 17/Bainong 64 | 2008 | Jiangsu | YHWZ |
| 282 | Huaimai 22 | white | Huaimai 18/Yangmai 158 | 2007 | Jiangsu | YHWZ |
| 283 | Huaimai 28 | white | Zhoumai 13/Xinmai 9 | 2009 | Jiangsu | YHWZ |
| 284 | Huaimai 29 | white | Huaimai 20/Mianyang 04254 | 2009 | Jiangsu | YHWZ |
| 285 | Lianmai 2 | white | Yumai 49 variant | 2005 | Jiangsu | YHWZ |
| 286 | Xumai 30 | white | Zhou 91098/Xuzhou 25 | 2007 | Jiangsu | YHWZ |
| 287 | Xumai 856 | white | Zhengzhou 8329/Xuzhou 86195-14-4-4-1 | 2006 | Jiangsu | YHWZ |
| 288 | Xuzhou 24 | white | 7904-13-2-2/Aimengniu | 1994 | Jiangsu | YHWZ |
| 289 | Xuzhou 25 | white | 7904-13-2-2/Bainong 792 | 2000 | Jiangsu | YHWZ |
| 290 | Huacheng 3366 | white | Yannong 361/Su 266 | 2013 | Hubei | MLWZ |
| 291 | Huamai 2566 | red | Een 1/Hua 9528//Jiayin 175/6280 | 2008 | Hubei | MLWZ |
| 292 | Lunxuan 146 | white | Zhengmai 9023/Zhoumai 18//Zhoumai 18 | 2018 | Hubei | MLWZ |
| 293 | Xianmai 8 | white | Wanmai 369/Zhengmai 9023 | 2013 | Hubei | MLWZ |
| 294 | Nannong 0686 | red | MV964091/Ningchun 9 | 2010 | Jiangsu | MLWZ |
| 295 | Ningmai 13 | red | Ningmai 9 pedigree selection | 2006 | Jiangsu | MLWZ |
| 296 | Ningmai 16 | red | Ningmai 8/Ningmai 9 | 2009 | Jiangsu | MLWZ |
| 297 | Ningmai 9 | red | Changwu 131/Jinmai 14 | 2010 | Jiangsu | MLWZ |
| 298 | Shengxuan 6 | red | Ningmai 8/Ningmai 9 | 2009 | Jiangsu | MLWZ |
| 299 | Yangfumai 4 | red | Ningmai 8/Ningmai 9 | 2005 | Jiangsu | MLWZ |
| 300 | Yangmai 12 | red | Yangmai 158/3/TP114/Yangmai 5//85-853 | 2001 | Jiangsu | MLWZ |
| 301 | Yangmai 13 | red | Yang 88-84//Yangmai 3/Marist Dove | 2003 | Jiangsu | MLWZ |
| 302 | Yangmai 15 | red | Yang 89-40/Chuanyu 21536 | 2005 | Jiangsu | MLWZ |
| 303 | Yangmai 158 | red | Yangmai 4/St1472/506 | 1993 | Jiangsu | MLWZ |
| 304 | Yangmai 16 | red | Yang 91F138/Yang 90-3 | 2004 | Jiangsu | MLWZ |
| 305 | Yangmai 20 | red | Yangmai 9/Yangmai 10 | 2010 | Jiangsu | MLWZ |
| 306 | Yangmai 21 | red | Ningmai 9/Hongqumang | 2011 | Jiangsu | MLWZ |
| 307 | Yangmai 22 | red | Yangmai 9*3/97033-2 | 2012 | Jiangsu | MLWZ |
| 308 | Yangmai 23 | red | Yangmai 16/Yangfu 93-11 | 2013 | Jiangsu | MLWZ |
| 309 | Zhenmai 168 | red | Shumai 6/97G59 | 2007 | Jiangsu | MLWZ |
| 310 | Zhenmai 9 | red | Shumai 6/97G59 | 2010 | Jiangsu | MLWZ |
| 311 | Chuanmai 22 | red | Mianyang 11/Chuanmai 20 | 2001 | Sichuan | SWWZ |
| 312 | Chuanmai 24 | red | 8282-15/Mianyang 19 | 1994 | Sichuan | SWWZ |
| 313 | Chuanmai 28 | red | Wanya 2/77 Zhong 2874/Gaojiasuo/77 Zhong 2874/3/Mianyang 19 | 1997 | Sichuan | SWWZ |
| 314 | Chuanmai 42 | red | Syn-CD 768/SW 3243//Chuan 6415 | 2004 | Sichuan | SWWZ |
| 315 | Chuanmai 46 | white | Ta193-6280/96-5429 | 2005 | Sichuan | SWWZ |
| 316 | Chuanmai 47 | white | Syn-CD 768/Mianyang 26 | 2005 | Sichuan | SWWZ |
| 317 | Chuanmai 64 | red | Chuanmai 42/Chuanmai 16 | 2013 | Sichuan | SWWZ |
| 318 | Chuanmai 107 | white | 2496/80-28-7 | 2000 | Sichuan | SWWZ |
| 319 | Chuannong 16 | red | Chuanyu 12/87-429 | 2002 | Sichuan | SWWZ |
| 320 | Miannong 4 | red | (75-21-4/76-19)/(Miangyang 11/Alondras) | 1993 | Sichuan | SWWZ |
| 321 | Mianyang 11 | red | 70-5858/Fan 6 | 1984 | Sichuan | SWWZ |
| 322 | Mianyang 19 | red | Mianyang 11 pedigree selection | 1985 | Sichuan | SWWZ |
| 323 | Mianyang 20 | white | Miangyang 11 variant | 1988 | Sichuan | SWWZ |
| 324 | Mianyang 26 | white | Miangyang 20/Chuangyu 9 | 1995 | Sichuan | SWWZ |
| 325 | Neimai 8 | white | Mianyang 26/92R178 | 2003 | Sichuan | SWWZ |
| 326 | Neimai 9 | white | Mianyang26/92R178 | 2006 | Sichuan | SWWZ |

NWWZ, Northern Winter Wheat Zone; YHWZ, Yellow and Huai River Valleys Winter Wheat Zone; MLWZ, Middle and Lower Yangtze River Valleys Winter Wheat Zone; SWWZ, Southwestern Winter Wheat Zone.

**Table S2 Functional markers associated with pre-harvest sprouting resistance in wheat**

| Gene | Chromosome | Allele | Primer sequence (5ʹ-3ʹ) | Fragment size (bp) | Tm (℃) | Reference |
| --- | --- | --- | --- | --- | --- | --- |
| *TaSdr-A1* | 2AS | *TaSdr-A1a* | F: CGTCGGCAGACATCGACTCC | 1146 | 59 | Zhang et al. (2017) |
|  |  | *TaSdr-A1b* | R: GAAGCTCACTAGCTCAGAACACGC | 528, 618 |  |  |
| *TaSdr-B1* | 2BS | *TaSdr-B1a* | F: TCAGCAGACTTCGACTCGCA-FAM |  |  | In this study |
|  |  | *TaSdr-B1b* | F: TCAGCAGACTTCGACTCGCG-HEX |  |  |  |
|  |  |  | R: AGGATCTCGTTGGCCTTGACG |  |  |  |
| *Tamyb10-A1* | 3AL | *Tamyb10-A1a* | F: CTATGTGGATGGCCTTGGAT | 665 | 56 | Himi et al. (2011) |
|  |  |  | R: CTACCAGCTCGTTTGGGAAG |  |  |  |
|  |  | *Tamyb10-A1b* | F: TTTCAATCGAGTGGGCATAA | 536 | 56 |  |
|  |  |  | R: CCTGACGATGAGCTCCTCTT |  |  |  |
|  |  | *Tamyb10-A1a** | F: TCCCTACATGGGAGACAGAGA | 2750 | 56 |  |
|  |  |  | R: TGTTATCACATGCTGATCCTGA |  |  |  |
| *Tamyb10-B1* | 3BL | *Tamyb10-B1a* | F: AGCAAGAGGAACCTGCAGTC | 263 | 58 | Himi et al. (2011) |
|  |  | *Tamyb10-B1b* | R: GATGCCCTCCAGATCAAGGT | 282 |  |  |
| *Tamyb10-D1* | 3DL | *Tamyb10-D1a* | F: TAGGCCAACACCTTCTAAAC | — | 60 | Himi et al. (2011) |
|  |  | *Tamyb10-D1b* | R: AGGCACACCAGCTTATTTGG | 1353 |  |  |
| *TaVp-1B* | *3BL* | *Vp-1Ba, Vp-1Bb,*  *Vp-1Bc, Vp1-Bd,*  *Vp1-Be, Vp1-Bf* | F: TGCTCCTTTCCCAATTGG | 532, 725,  449, 507,  453, 616 | 60 | Chang et al. (2010) |
|  |  |  | R: TGCTTCTCTTCTCTCACCAGTG |  |  |  |
| *TaDFR-B* | 3BL | *TaDFR-Bb* | F: TGCGGTCTGGCGGGGTACGT | 432 | 60 | Bi et al. (2014) |
|  |  | *TaDFR-Ba/c* | R: ACGTCGAGAGAGAGAGGGAGGGG | 526 |  |  |
| *TaMKK3-A* | 4AL | *TaMKK3-Aa* | F: CACCAAAGAATAGAAATGCTCTCT | 887 | 62 | Torada et al. (2016) |
|  |  | *TaMKK3-Ab* | R: AGGAGTAGTTCTCATTGCGG | 605, 282 |  |  |
| *TaGASR34* | 7B | *GS34-7Ba* | F: ACACCTCGGTTCAATGCC | 900, 410 | 60 | Cheng et al. (2019) |
|  |  | *GS34-7Bb* | R: CCTTCTTGTACTGCGTCCC | 1310 |  |  |
| *TaMFT* (*TaPHS1*) | 3AS | *TaMFT-3Aa* | F: GGCTACGTGTCGCTTGAC | 252 | 56 | Jiang et al. (2018) |
|  | - 194 (33 bp InDel) | *TaMFT-3Ab* | R: GCGGCGGATTATTAACTG | 219 |  |  |
|  | - 222 (C/T) | *TaPHS1-222C* | F: TCACGCATCAGCGATCGAC-FAM |  |  | Shao et al. (2018) |
|  |  | *TaPHS1-222T* | F: TCACGCATCAGCGATCGAT -HEX |  |  |  |
|  |  |  | R: GCTTACGCTAAGCAGGTGGCTA |  |  |  |
|  | + 219 (12 bp InDel) | *TaMFT-A1a* | F: GAGCAAACATGTCCCGGTTCGTT | 331 | 57 | Lei et al. (2013) |
|  |  | *TaMFT-A1b* | R: ATCACCATGCACACACATACATAAATCACC | 319 |  |  |
|  | + 646 (G/A) | *TaPHS1-646G* | F: GGTGGAACAGATGCAACTAAAGG -FAM |  |  | Liu et al. (2013); |
|  |  | *TaPHS1-646A* | F: GGTGGAACAGATGCAACTAAAGA -HEX |  |  | Rasheed et al. (2016) |
|  |  |  | R: GTGAGTGTTATATGAAACTAATGATCCATT | |  |  |
|  | + 666 (T/A) | *TaPHS1-666T* | F: GTGAGTGTTATATGAAACTAATGATCCATTT-FAM | |  | Liu et al. (2013);  Rasheed et al. (2016) |
|  |  | *TaPHS1-666A* | F: GTGAGTGTTATATGAAACTAATGATCCATTA -HEX | |  |  |
|  |  |  | R: ACCGGGTGGAACAGATGCAACTAAA |  |  |  |

**Table S3** Analysis of variance for germination index (GI) in 326 winter wheat cultivars across three years

| Source | df | Mean square |
| --- | --- | --- |
| Genotype (G) | 325 | 0.26^**^ |
| Year (Y) | 2 | 15.15^**^ |
| G × Y interaction | 650 | 0.03^**^ |
| Error | 1954 | 0.01 |

**: significant at *P* ≤ 0.01.

**Table S4** Allelic variation of 10 genes associated with pre-harvest sprouting resistance and germination index (GI) in the 326 winter wheat cultivars

| Code number | Name | *TaSdr-A1* | *TaSdr-B1* | *Tamyb10-A1* | *Tamyb10-B1* | *Tamyb10-D1* | *TaVp-1B* | *TaDFR-B* | *TaMKK3-A* | *TaGASR34* | *TaMFT* | | | | | 2018GI | 2019GI | 2020GI |
| --- | --- | --- | --- | --- | --- | --- | --- | --- | --- | --- | --- | --- | --- | --- | --- | --- | --- | --- |
|  |  |  |  |  |  |  |  |  |  |  | - 194 | - 222 | + 219 | + 646 | + 666 |  |  |  |
| 1 | Jing 411 | *TaSdr-A1a* | *TaSdr-B1b* | *Tamyb10-A1a* | *Tamyb10-B1a* | *Tamyb10-D1a* | *Vp-1Ba* | *TaDFR-Ba* | *TaMKK3-Ab* | *GS34-7Ba* | *TaMFT-3Aa* | *TaPHS1-222T* | *TaMFT-A1a* | *TaPHS1-646G* | *TaPHS1-666A* | 79.00% | 72.30% | 62.60% |
| 2 | Jing 9428 | *TaSdr-A1a* | *TaSdr-B1b* | *Tamyb10-A1b* | *Tamyb10-B1b* | *Tamyb10-D1a* | *Vp-1Bc* | *TaDFR-Ba* | *TaMKK3-Ab* | *GS34-7Ba* | *TaMFT-3Aa* | *TaPHS1-222T* | *TaMFT-A1b* | *TaPHS1-646A* | *TaPHS1-666T* | 61.10% | 57.60% | 16.50% |
| 3 | Jingdong 8 | *TaSdr-A1a* | *TaSdr-B1b* | *Tamyb10-A1a* | *Tamyb10-B1b* | *Tamyb10-D1b* | *Vp-1Bc* | *TaDFR-Bb* | *TaMKK3-Aa* | *GS34-7Ba* | *TaMFT-3Ab* | *TaPHS1-222T* | *TaMFT-A1a* | *TaPHS1-646A* | *TaPHS1-666T* | 72.60% | 67.60% | 56.60% |
| 4 | Jingdong 12 | *TaSdr-A1a* | *TaSdr-B1b* | *Tamyb10-A1a* | *Tamyb10-B1b* | *Tamyb10-D1a* | *Vp-1Bc* | *TaDFR-Bb* | *TaMKK3-Aa* | *GS34-7Ba* | *TaMFT-3Ab* | *TaPHS1-222T* | *TaMFT-A1a* | *TaPHS1-646A* | *TaPHS1-666T* | 81.40% | 79.20% | 45.70% |
| 5 | Jingdong 17 | *TaSdr-A1a* | *TaSdr-B1b* | *Tamyb10-A1a* | *Tamyb10-B1a* | *Tamyb10-D1a* | *Vp-1Bc* | *TaDFR-Ba* | *TaMKK3-Aa* | *GS34-7Ba* | *TaMFT-3Aa* | *TaPHS1-222T* | *TaMFT-A1a* | *TaPHS1-646A* | *TaPHS1-666T* | 82.40% | 75.70% | 50.70% |
| 6 | Jingdong 22 | *TaSdr-A1a* | *TaSdr-B1b* | *Tamyb10-A1b* | *Tamyb10-B1b* | *Tamyb10-D1b* | *Vp-1Ba* | *TaDFR-Bb* | *TaMKK3-Aa* | *GS34-7Ba* | *TaMFT-3Ab* | *TaPHS1-222T* | *TaMFT-A1a* | *TaPHS1-646G* | *TaPHS1-666T* | 64.80% | 79.60% | 47.90% |
| 7 | Jinghua 9 | *TaSdr-A1a* | *TaSdr-B1b* | *Tamyb10-A1a* | *Tamyb10-B1b* | *Tamyb10-D1a* | *Vp-1Bc* | *TaDFR-Bb* | *TaMKK3-Aa* | *GS34-7Ba* | *TaMFT-3Ab* | *TaPHS1-222T* | *TaMFT-A1a* | *TaPHS1-646A* | *TaPHS1-666T* | 64.60% | 67.40% | 24.60% |
| 8 | Jinmai 47 | *TaSdr-A1a* | *TaSdr-B1b* | *Tamyb10-A1b* | *Tamyb10-B1a* | *Tamyb10-D1a* | *Vp-1Bc* | *TaDFR-Ba* | *TaMKK3-Aa* | *GS34-7Ba* | *TaMFT-3Ab* | *TaPHS1-222T* | *TaMFT-A1a* | *TaPHS1-646G* | *TaPHS1-666A* | 44.20% | 75.90% | 22.10% |
| 9 | Lunxuan 167 | *TaSdr-A1a* | *TaSdr-B1a* | *Tamyb10-A1b* | *Tamyb10-B1a* | *Tamyb10-D1a* | *Vp-1Ba* | *TaDFR-Bb* | *TaMKK3-Aa* | *GS34-7Ba* | *TaMFT-3Ab* | *TaPHS1-222T* | *TaMFT-A1a* | *TaPHS1-646A* | *TaPHS1-666T* | 51.00% | 61.90% | 26.20% |
| 10 | Lunxuan 169 | *TaSdr-A1b* | *TaSdr-B1b* | *Tamyb10-A1b* | *Tamyb10-B1a* | *Tamyb10-D1a* | *Vp-1Ba* | *TaDFR-Bb* | *—* | *GS34-7Ba* | *TaMFT-3Aa* | *TaPHS1-222T* | *TaMFT-A1a* | *TaPHS1-646G* | *TaPHS1-666A* | 42.00% | 37.00% | 28.10% |
| 11 | Lunxuan 987 | *TaSdr-A1a* | *TaSdr-B1a* | *Tamyb10-A1a* | *Tamyb10-B1b* | *Tamyb10-D1a* | *Vp-1Ba* | *TaDFR-Bb* | *TaMKK3-Aa* | *GS34-7Ba* | *TaMFT-3Aa* | *TaPHS1-222T* | *TaMFT-A1b* | *TaPHS1-646G* | *TaPHS1-666A* | 31.90% | 42.00% | 26.30% |
| 12 | Ningchun 4 | *TaSdr-A1b* | *TaSdr-B1b* | *Tamyb10-A1b* | *Tamyb10-B1a* | *Tamyb10-D1a* | *Vp-1Bc* | *TaDFR-Bb* | *TaMKK3-Ab* | *GS34-7Ba* | *TaMFT-3Ab* | *TaPHS1-222T* | *TaMFT-A1a* | *TaPHS1-646G* | *TaPHS1-666T* | 71.00% | 60.20% | 52.50% |
| 13 | Nongda 3432 | *TaSdr-A1a* | *TaSdr-B1b* | *Tamyb10-A1a* | *Tamyb10-B1a* | *Tamyb10-D1a* | *Vp-1Bc* | *TaDFR-Bb* | *TaMKK3-Aa* | *GS34-7Ba* | *TaMFT-3Aa* | *TaPHS1-222T* | *TaMFT-A1a* | *TaPHS1-646A* | *TaPHS1-666T* | 55.90% | 35.80% | 29.40% |
| 14 | Xindong 20 | *TaSdr-A1a* | *TaSdr-B1b* | *Tamyb10-A1a* | *Tamyb10-B1a* | *Tamyb10-D1a* | *Vp-1Bc* | *TaDFR-Bb* | *TaMKK3-Ab* | *GS34-7Ba* | *TaMFT-3Aa* | *TaPHS1-222T* | *TaMFT-A1b* | *TaPHS1-646G* | *TaPHS1-666A* | 58.40% | 85.10% | 43.30% |
| 15 | Xindong 22 | *TaSdr-A1a* | *TaSdr-B1a* | *Tamyb10-A1a* | *Tamyb10-B1a* | *Tamyb10-D1a* | *Vp-1Ba* | *TaDFR-Bb* | *TaMKK3-Ab* | *GS34-7Bb* | *TaMFT-3Aa* | *TaPHS1-222T* | *TaMFT-A1b* | *TaPHS1-646A* | *TaPHS1-666A* | 23.20% | 30.50% | 15.90% |
| 16 | Zhongmai 8 | *TaSdr-A1a* | *TaSdr-B1a* | *Tamyb10-A1a* | *Tamyb10-B1a* | *Tamyb10-D1a* | *Vp-1Bc* | *TaDFR-Ba* | *TaMKK3-Ab* | *GS34-7Ba* | *TaMFT-3Aa* | *TaPHS1-222T* | *TaMFT-A1b* | *TaPHS1-646G* | *TaPHS1-666A* | 83.00% | 90.00% | 54.30% |
| 17 | Zhongmai 9 | *TaSdr-A1a* | *TaSdr-B1b* | *Tamyb10-A1a* | *Tamyb10-B1a* | *Tamyb10-D1a* | *Vp-1Ba* | *TaDFR-Ba* | *TaMKK3-Aa* | *GS34-7Ba* | *TaMFT-3Ab* | *TaPHS1-222T* | *TaMFT-A1a* | *TaPHS1-646A* | *TaPHS1-666T* | 83.00% | 79.30% | 57.20% |
| 18 | Zhongmai 175 | *TaSdr-A1a* | *TaSdr-B1b* | *Tamyb10-A1a* | *Tamyb10-B1a* | *Tamyb10-D1a* | *Vp-1Ba* | *TaDFR-Ba* | *TaMKK3-Aa* | *GS34-7Ba* | *TaMFT-3Ab* | *TaPHS1-222T* | *TaMFT-A1a* | *TaPHS1-646A* | *TaPHS1-666T* | 93.70% | 90.30% | 82.10% |
| 19 | Zhongmai 415 | *TaSdr-A1a* | *TaSdr-B1b* | *Tamyb10-A1a* | *Tamyb10-B1a* | *Tamyb10-D1a* | *Vp-1Bc* | *TaDFR-Ba* | *TaMKK3-Ab* | *GS34-7Ba* | *TaMFT-3Aa* | *TaPHS1-222T* | *TaMFT-A1b* | *TaPHS1-646A* | *TaPHS1-666T* | 66.70% | 67.40% | 55.90% |
| 20 | Cangmai 6005 | *TaSdr-A1a* | *TaSdr-B1b* | *Tamyb10-A1b* | *Tamyb10-B1a* | *Tamyb10-D1a* | *Vp-1Ba* | *TaDFR-Bb* | *TaMKK3-Aa* | *GS34-7Ba* | *TaMFT-3Aa* | *TaPHS1-222T* | *TaMFT-A1b* | *TaPHS1-646G* | *TaPHS1-666A* | 27.80% | 35.80% | 22.20% |
| 21 | Gao 2018 | *TaSdr-A1a* | *TaSdr-B1b* | *Tamyb10-A1a* | *Tamyb10-B1a* | *Tamyb10-D1a* | *Vp-1Ba* | *TaDFR-Bb* | *TaMKK3-Aa* | *GS34-7Ba* | *TaMFT-3Aa* | *TaPHS1-222T* | *TaMFT-A1a* | *TaPHS1-646G* | *TaPHS1-666A* | 28.90% | 27.50% | 9.80% |
| 22 | Gaocheng 8901 | *TaSdr-A1a* | *TaSdr-B1b* | *Tamyb10-A1a* | *Tamyb10-B1a* | *Tamyb10-D1a* | *Vp-1Bc* | *TaDFR-Bb* | *TaMKK3-Ab* | *GS34-7Ba* | *TaMFT-3Ab* | *TaPHS1-222T* | *TaMFT-A1a* | *TaPHS1-646G* | *TaPHS1-666T* | 52.40% | 62.60% | 33.20% |
| 23 | Gaocheng 9415 | *TaSdr-A1a* | *TaSdr-B1b* | *Tamyb10-A1a* | *Tamyb10-B1a* | *Tamyb10-D1a* | *Vp-1Bc* | *TaDFR-Bb* | *TaMKK3-Ab* | *GS34-7Ba* | *TaMFT-3Ab* | *TaPHS1-222T* | *TaMFT-A1a* | *TaPHS1-646A* | *TaPHS1-666T* | 44.20% | 42.80% | 12.20% |
| 24 | Gaoyou 503 | *TaSdr-A1a* | *TaSdr-B1b* | *Tamyb10-A1a* | *Tamyb10-B1a* | *Tamyb10-D1a* | *Vp-1Bc* | *TaDFR-Bb* | *TaMKK3-Ab* | *GS34-7Ba* | *TaMFT-3Aa* | *TaPHS1-222T* | *TaMFT-A1b* | *TaPHS1-646G* | *TaPHS1-666A* | 49.40% | 54.60% | 14.30% |
| 25 | Gaoyou 9618 | *TaSdr-A1a* | *TaSdr-B1b* | *Tamyb10-A1a* | *Tamyb10-B1a* | *Tamyb10-D1a* | *Vp-1Bc* | *TaDFR-Bb* | *TaMKK3-Ab* | *GS34-7Ba* | *TaMFT-3Ab* | *TaPHS1-222T* | *TaMFT-A1a* | *TaPHS1-646G* | *TaPHS1-666T* | 68.50% | 75.70% | 64.60% |
| 26 | Han 4564 | *TaSdr-A1a* | *TaSdr-B1b* | *Tamyb10-A1a* | *Tamyb10-B1a* | *Tamyb10-D1a* | *Vp-1Bc* | *TaDFR-Ba* | *TaMKK3-Ab* | *GS34-7Bb* | *TaMFT-3Aa* | *TaPHS1-222T* | *TaMFT-A1b* | *TaPHS1-646G* | *TaPHS1-666A* | 82.30% | 84.70% | 82.10% |
| 27 | Han 4589 | *TaSdr-A1a* | *TaSdr-B1a* | *Tamyb10-A1a* | *Tamyb10-B1a* | *Tamyb10-D1a* | *Vp-1Ba* | *TaDFR-Bb* | *TaMKK3-Ab* | *GS34-7Ba* | *TaMFT-3Aa* | *TaPHS1-222T* | *TaMFT-A1a* | *TaPHS1-646G* | *TaPHS1-666A* | 86.80% | 81.70% | 71.00% |
| 28 | Han 5316 | *TaSdr-A1a* | *TaSdr-B1b* | *Tamyb10-A1a* | *Tamyb10-B1a* | *Tamyb10-D1a* | *Vp-1Bc* | *TaDFR-Bb* | *TaMKK3-Aa* | *GS34-7Ba* | *TaMFT-3Aa* | *TaPHS1-222T* | *TaMFT-A1b* | *TaPHS1-646G* | *TaPHS1-666A* | 21.10% | 30.60% | 10.30% |
| 29 | Han 6172 | *TaSdr-A1b* | *TaSdr-B1b* | *Tamyb10-A1a* | *Tamyb10-B1a* | *Tamyb10-D1a* | *Vp-1Ba* | *TaDFR-Bb* | *TaMKK3-Ab* | *GS34-7Ba* | *TaMFT-3Aa* | *TaPHS1-222T* | *TaMFT-A1a* | *TaPHS1-646G* | *TaPHS1-666A* | 84.40% | 86.60% | 75.00% |
| 30 | Hanmai 11 | *TaSdr-A1a* | *TaSdr-B1a* | *Tamyb10-A1a* | *Tamyb10-B1a* | *Tamyb10-D1a* | *Vp-1Ba* | *TaDFR-Bb* | *TaMKK3-Aa* | *GS34-7Ba* | *TaMFT-3Aa* | *TaPHS1-222T* | *TaMFT-A1b* | *TaPHS1-646G* | *TaPHS1-666A* | 45.30% | 46.90% | 35.10% |
| 31 | Hanmai 13 | *TaSdr-A1a* | *TaSdr-B1b* | *Tamyb10-A1a* | *Tamyb10-B1b* | *Tamyb10-D1a* | *Vp-1Bc* | *TaDFR-Ba* | *TaMKK3-Ab* | *GS34-7Bb* | *TaMFT-3Aa* | *TaPHS1-222T* | *TaMFT-A1b* | *TaPHS1-646A* | *TaPHS1-666T* | 19.90% | 32.60% | 5.40% |
| 32 | Hanmai 14 | *TaSdr-A1a* | *TaSdr-B1b* | *Tamyb10-A1a* | *Tamyb10-B1a* | *Tamyb10-D1a* | *Vp-1Ba* | *TaDFR-Ba* | *TaMKK3-Ab* | *GS34-7Ba* | *TaMFT-3Aa* | *TaPHS1-222T* | *TaMFT-A1b* | *TaPHS1-646G* | *TaPHS1-666A* | 68.30% | 83.00% | 76.70% |
| 33 | Hanmai 16 | *TaSdr-A1a* | *TaSdr-B1b* | *Tamyb10-A1a* | *Tamyb10-B1a* | *Tamyb10-D1a* | *Vp-1Bc* | *TaDFR-Ba* | *TaMKK3-Ab* | *GS34-7Ba* | *TaMFT-3Ab* | *TaPHS1-222T* | *TaMFT-A1a* | *TaPHS1-646A* | *TaPHS1-666T* | 46.20% | 54.20% | 45.00% |
| 34 | Hanmai 17 | *TaSdr-A1a* | *TaSdr-B1b* | *Tamyb10-A1a* | *Tamyb10-B1a* | *Tamyb10-D1a* | *Vp-1Ba* | *TaDFR-Bb* | *TaMKK3-Aa* | *GS34-7Ba* | *TaMFT-3Aa* | *TaPHS1-222T* | *TaMFT-A1a* | *TaPHS1-646G* | *TaPHS1-666A* | 45.80% | 64.40% | 22.50% |
| 35 | Heng 4399 | *TaSdr-A1a* | *TaSdr-B1b* | *Tamyb10-A1a* | *Tamyb10-B1a* | *Tamyb10-D1a* | *Vp-1Ba* | *TaDFR-Bb* | *TaMKK3-Aa* | *GS34-7Ba* | *TaMFT-3Aa* | *TaPHS1-222T* | *TaMFT-A1a* | *TaPHS1-646G* | *TaPHS1-666A* | 77.00% | 85.00% | 63.10% |
| 36 | Heng 5229 | *TaSdr-A1a* | *TaSdr-B1b* | *Tamyb10-A1a* | *Tamyb10-B1a* | *Tamyb10-D1a* | *Vp-1Bc* | *TaDFR-Ba* | *TaMKK3-Ab* | *GS34-7Ba* | *TaMFT-3Ab* | *TaPHS1-222T* | *TaMFT-A1a* | *TaPHS1-646A* | *TaPHS1-666T* | 65.10% | 67.40% | 31.00% |
| 37 | Heng 6632 | *TaSdr-A1a* | *TaSdr-B1b* | *Tamyb10-A1a* | *Tamyb10-B1a* | *Tamyb10-D1a* | *Vp-1Bc* | *TaDFR-Ba* | *TaMKK3-Ab* | *GS34-7Ba* | *TaMFT-3Ab* | *TaPHS1-222T* | *TaMFT-A1a* | *TaPHS1-646G* | *TaPHS1-666T* | 67.20% | 75.20% | 56.90% |
| 38 | Heng 7228 | *TaSdr-A1a* | *TaSdr-B1b* | *Tamyb10-A1a* | *Tamyb10-B1a* | *Tamyb10-D1a* | *Vp-1Bc* | *TaDFR-Ba* | *TaMKK3-Ab* | *GS34-7Ba* | *TaMFT-3Aa* | *TaPHS1-222T* | *TaMFT-A1a* | *TaPHS1-646G* | *TaPHS1-666A* | 50.90% | 58.90% | 42.50% |
| 39 | Hengguan 35 | *TaSdr-A1a* | *TaSdr-B1b* | *Tamyb10-A1a* | *Tamyb10-B1a* | *Tamyb10-D1a* | *Vp-1Bc* | *TaDFR-Ba* | *TaMKK3-Aa* | *GS34-7Ba* | *TaMFT-3Ab* | *TaPHS1-222T* | *TaMFT-A1b* | *TaPHS1-646A* | *TaPHS1-666T* | 59.50% | 81.00% | 59.10% |
| 40 | Henong 326 | *TaSdr-A1a* | *TaSdr-B1b* | *Tamyb10-A1a* | *Tamyb10-B1a* | *Tamyb10-D1a* | *Vp-1Bc* | *TaDFR-Bb* | *TaMKK3-Aa* | *GS34-7Ba* | *TaMFT-3Aa* | *TaPHS1-222C* | *TaMFT-A1b* | *TaPHS1-646G* | *TaPHS1-666A* | 22.80% | 32.60% | 15.70% |
| 41 | Henong 6049 | *TaSdr-A1a* | *TaSdr-B1b* | *Tamyb10-A1a* | *Tamyb10-B1a* | *Tamyb10-D1a* | *Vp-1Bc* | *TaDFR-Bb* | *TaMKK3-Ab* | *GS34-7Bb* | *TaMFT-3Aa* | *TaPHS1-222C* | *TaMFT-A1b* | *TaPHS1-646G* | *TaPHS1-666A* | 17.00% | 25.00% | 18.30% |
| 42 | Henong 822 | *TaSdr-A1a* | *TaSdr-B1b* | *Tamyb10-A1a* | *Tamyb10-B1a* | *Tamyb10-D1a* | *Vp-1Ba* | *TaDFR-Bb* | *TaMKK3-Aa* | *GS34-7Bb* | *TaMFT-3Ab* | *TaPHS1-222T* | *TaMFT-A1a* | *TaPHS1-646A* | *TaPHS1-666T* | 78.00% | 74.90% | 46.90% |
| 43 | Henong 972 | *TaSdr-A1b* | *TaSdr-B1b* | *Tamyb10-A1a* | *Tamyb10-B1a* | *Tamyb10-D1a* | *Vp-1Bb* | *TaDFR-Bb* | *TaMKK3-Aa* | *GS34-7Ba* | *TaMFT-3Aa* | *TaPHS1-222C* | *TaMFT-A1b* | *TaPHS1-646G* | *TaPHS1-666A* | 61.50% | 83.80% | 47.90% |
| 44 | Ji 5265 | *TaSdr-A1a* | *TaSdr-B1b* | *Tamyb10-A1a* | *Tamyb10-B1a* | *Tamyb10-D1a* | *Vp-1Bc* | *TaDFR-Ba* | *TaMKK3-Ab* | *GS34-7Ba* | *TaMFT-3Aa* | *TaPHS1-222T* | *TaMFT-A1b* | *TaPHS1-646G* | *TaPHS1-666A* | 52.50% | 67.00% | 26.20% |
| 45 | Jimai 26 | *TaSdr-A1a* | *TaSdr-B1b* | *Tamyb10-A1a* | *Tamyb10-B1a* | *Tamyb10-D1a* | *Vp-1Bc* | *TaDFR-Ba* | *TaMKK3-Ab* | *GS34-7Bb* | *TaMFT-3Aa* | *TaPHS1-222T* | *TaMFT-A1a* | *TaPHS1-646G* | *TaPHS1-666A* | 65.80% | 84.60% | 45.50% |
| 46 | Jimai 30 | *TaSdr-A1a* | *TaSdr-B1b* | *Tamyb10-A1a* | *Tamyb10-B1a* | *Tamyb10-D1a* | *Vp-1Ba* | *TaDFR-Bb* | *TaMKK3-Ab* | *GS34-7Ba* | *TaMFT-3Ab* | *TaPHS1-222T* | *TaMFT-A1a* | *TaPHS1-646A* | *TaPHS1-666T* | 43.60% | 78.00% | 54.50% |
| 47 | Jimai 325 | *TaSdr-A1a* | *TaSdr-B1b* | *Tamyb10-A1a* | *Tamyb10-B1a* | *Tamyb10-D1a* | *Vp-1Bb* | *TaDFR-Ba* | *TaMKK3-Ab* | *GS34-7Ba* | *TaMFT-3Aa* | *TaPHS1-222T* | *TaMFT-A1b* | *TaPHS1-646G* | *TaPHS1-666A* | 71.00% | 88.00% | 87.70% |
| 48 | Jimai 36 | *TaSdr-A1a* | *TaSdr-B1b* | *Tamyb10-A1a* | *Tamyb10-B1a* | *Tamyb10-D1a* | *Vp-1Bb* | *TaDFR-Bb* | *TaMKK3-Ab* | *GS34-7Ba* | *TaMFT-3Aa* | *TaPHS1-222T* | *TaMFT-A1a* | *TaPHS1-646G* | *TaPHS1-666A* | 59.00% | 54.40% | 55.60% |
| 49 | Jimai 38 | *TaSdr-A1a* | *TaSdr-B1b* | *Tamyb10-A1b* | *Tamyb10-B1a* | *Tamyb10-D1a* | *Vp-1Bc* | *TaDFR-Ba* | *—* | *GS34-7Bb* | *TaMFT-3Ab* | *TaPHS1-222T* | *TaMFT-A1a* | *TaPHS1-646G* | *TaPHS1-666A* | 71.50% | 86.70% | 69.50% |
| 50 | Jimai 418 | *TaSdr-A1a* | *TaSdr-B1b* | *Tamyb10-A1b* | *Tamyb10-B1a* | *Tamyb10-D1a* | *Vp-1Bc* | *TaDFR-Ba* | *TaMKK3-Ab* | *GS34-7Bb* | *TaMFT-3Aa* | *TaPHS1-222T* | *TaMFT-A1a* | *TaPHS1-646G* | *TaPHS1-666A* | 88.50% | 94.40% | 77.60% |
| 51 | Jimai 42 | *TaSdr-A1a* | *TaSdr-B1b* | *Tamyb10-A1b* | *Tamyb10-B1b* | *Tamyb10-D1a* | *Vp-1Bc* | *TaDFR-Bb* | *TaMKK3-Aa* | *GS34-7Ba* | *TaMFT-3Ab* | *TaPHS1-222T* | *TaMFT-A1a* | *TaPHS1-646A* | *TaPHS1-666T* | 57.60% | 83.30% | 58.60% |
| 52 | Jimai 585 | *TaSdr-A1a* | *TaSdr-B1a* | *Tamyb10-A1b* | *Tamyb10-B1a* | *Tamyb10-D1a* | *Vp-1Bc* | *TaDFR-Ba* | *TaMKK3-Ab* | *GS34-7Ba* | *TaMFT-3Ab* | *TaPHS1-222T* | *TaMFT-A1a* | *TaPHS1-646A* | *TaPHS1-666T* | 73.50% | 77.10% | 54.40% |
| 53 | Jinhe 9123 | *TaSdr-A1a* | *TaSdr-B1a* | *Tamyb10-A1a* | *Tamyb10-B1a* | *Tamyb10-D1a* | *Vp-1Bc* | *TaDFR-Ba* | *TaMKK3-Ab* | *GS34-7Ba* | *TaMFT-3Ab* | *TaPHS1-222T* | *TaMFT-A1a* | *TaPHS1-646A* | *TaPHS1-666T* | 78.00% | 89.70% | 62.00% |
| 54 | Kenong 199 | *TaSdr-A1a* | *TaSdr-B1b* | *Tamyb10-A1a* | *Tamyb10-B1a* | *Tamyb10-D1a* | *Vp-1Bc* | *TaDFR-Ba* | *TaMKK3-Ab* | *GS34-7Ba* | *TaMFT-3Aa* | *TaPHS1-222T* | *TaMFT-A1a* | *TaPHS1-646G* | *TaPHS1-666A* | 33.80% | 59.60% | 22.30% |
| 55 | Kenong 2009 | *TaSdr-A1a* | *TaSdr-B1b* | *Tamyb10-A1a* | *Tamyb10-B1a* | *Tamyb10-D1a* | *Vp-1Bc* | *TaDFR-Bb* | *TaMKK3-Ab* | *GS34-7Ba* | *TaMFT-3Ab* | *TaPHS1-222T* | *TaMFT-A1a* | *TaPHS1-646G* | *TaPHS1-666A* | 24.50% | 53.10% | 17.00% |
| 56 | Lunxuan 061 | *TaSdr-A1a* | *TaSdr-B1b* | *Tamyb10-A1a* | *Tamyb10-B1a* | *Tamyb10-D1a* | *Vp-1Ba* | *TaDFR-Bb* | *TaMKK3-Aa* | *GS34-7Ba* | *TaMFT-3Aa* | *TaPHS1-222T* | *TaMFT-A1a* | *TaPHS1-646A* | *TaPHS1-666T* | 12.40% | 31.40% | 12.10% |
| 57 | Lunxuan 103 | *TaSdr-A1a* | *TaSdr-B1b* | *Tamyb10-A1a* | *Tamyb10-B1a* | *Tamyb10-D1a* | *Vp-1Bb* | *TaDFR-Ba* | *TaMKK3-Aa* | *GS34-7Ba* | *TaMFT-3Ab* | *TaPHS1-222T* | *TaMFT-A1b* | *TaPHS1-646A* | *TaPHS1-666T* | 59.10% | 84.50% | 55.30% |
| 58 | Lunxuan 145 | *TaSdr-A1a* | *TaSdr-B1a* | *Tamyb10-A1a* | *Tamyb10-B1a* | *Tamyb10-D1a* | *Vp-1Ba* | *TaDFR-Bb* | *TaMKK3-Aa* | *GS34-7Ba* | *TaMFT-3Aa* | *TaPHS1-222T* | *TaMFT-A1a* | *TaPHS1-646G* | *TaPHS1-666A* | 55.60% | 51.50% | 35.90% |
| 59 | Shi 4185 | *TaSdr-A1a* | *TaSdr-B1a* | *Tamyb10-A1a* | *Tamyb10-B1a* | *Tamyb10-D1a* | *Vp-1Bc* | *TaDFR-Ba* | *TaMKK3-Ab* | *GS34-7Ba* | *TaMFT-3Ab* | *TaPHS1-222T* | *TaMFT-A1a* | *TaPHS1-646A* | *TaPHS1-666T* | 64.40% | 59.40% | 40.60% |
| 60 | Shi 4366 | *TaSdr-A1a* | *TaSdr-B1b* | *Tamyb10-A1a* | *Tamyb10-B1a* | *Tamyb10-D1a* | *Vp-1Bc* | *TaDFR-Ba* | *TaMKK3-Aa* | *GS34-7Ba* | *TaMFT-3Aa* | *TaPHS1-222T* | *TaMFT-A1a* | *TaPHS1-646A* | *TaPHS1-666T* | 50.70% | 48.10% | 33.00% |
| 61 | Shijiazhuang 8 | *TaSdr-A1a* | *TaSdr-B1b* | *Tamyb10-A1a* | *Tamyb10-B1a* | *Tamyb10-D1a* | *Vp-1Bc* | *TaDFR-Ba* | *TaMKK3-Ab* | *GS34-7Bb* | *TaMFT-3Ab* | *TaPHS1-222T* | *TaMFT-A1a* | *TaPHS1-646A* | *TaPHS1-666T* | 71.40% | 79.80% | 45.00% |
| 62 | Shiluan 02-1 | *TaSdr-A1a* | *TaSdr-B1b* | *Tamyb10-A1a* | *Tamyb10-B1a* | *Tamyb10-D1a* | *Vp-1Bc* | *TaDFR-Bb* | *TaMKK3-Aa* | *GS34-7Bb* | *TaMFT-3Ab* | *TaPHS1-222T* | *TaMFT-A1a* | *TaPHS1-646A* | *TaPHS1-666T* | 40.50% | 53.50% | 18.30% |
| 63 | Shimai 12 | *TaSdr-A1a* | *TaSdr-B1a* | *Tamyb10-A1a* | *Tamyb10-B1a* | *Tamyb10-D1a* | *Vp-1Ba* | *TaDFR-Bb* | *TaMKK3-Ab* | *GS34-7Ba* | *TaMFT-3Aa* | *TaPHS1-222T* | *TaMFT-A1b* | *TaPHS1-646G* | *TaPHS1-666A* | 38.20% | 57.60% | 35.30% |
| 64 | Shimai 14 | *TaSdr-A1a* | *TaSdr-B1a* | *Tamyb10-A1a* | *Tamyb10-B1a* | *Tamyb10-D1a* | *Vp-1Bc* | *TaDFR-Ba* | *TaMKK3-Ab* | *GS34-7Ba* | *TaMFT-3Ab* | *TaPHS1-222T* | *TaMFT-A1a* | *TaPHS1-646A* | *TaPHS1-666T* | 48.20% | 53.80% | 17.30% |
| 65 | Shimai 15 | *TaSdr-A1a* | *TaSdr-B1b* | *Tamyb10-A1a* | *Tamyb10-B1a* | *Tamyb10-D1a* | *Vp-1Bc* | *TaDFR-Ba* | *TaMKK3-Ab* | *GS34-7Bb* | *TaMFT-3Ab* | *TaPHS1-222T* | *TaMFT-A1a* | *TaPHS1-646A* | *TaPHS1-666T* | 63.30% | 86.60% | 79.60% |
| 66 | Shimai 18 | *TaSdr-A1a* | *TaSdr-B1a* | *Tamyb10-A1a* | *Tamyb10-B1a* | *Tamyb10-D1a* | *Vp-1Bc* | *TaDFR-Ba* | *TaMKK3-Ab* | *GS34-7Ba* | *TaMFT-3Aa* | *TaPHS1-222T* | *TaMFT-A1a* | *TaPHS1-646A* | *TaPHS1-666T* | 63.90% | 77.00% | 45.60% |
| 67 | Shimai 19 | *TaSdr-A1a* | *TaSdr-B1b* | *Tamyb10-A1a* | *Tamyb10-B1a* | *Tamyb10-D1a* | *Vp-1Ba* | *TaDFR-Ba* | *TaMKK3-Aa* | *GS34-7Ba* | *TaMFT-3Ab* | *TaPHS1-222T* | *TaMFT-A1a* | *TaPHS1-646A* | *TaPHS1-666T* | 52.10% | 66.00% | 46.10% |
| 68 | Shimai 22 | *TaSdr-A1a* | *TaSdr-B1b* | *Tamyb10-A1a* | *Tamyb10-B1a* | *Tamyb10-D1a* | *Vp-1Bc* | *TaDFR-Ba* | *TaMKK3-Ab* | *GS34-7Bb* | *TaMFT-3Ab* | *TaPHS1-222T* | *TaMFT-A1a* | *TaPHS1-646A* | *TaPHS1-666T* | 76.10% | 92.20% | 80.60% |
| 69 | Shinong 086 | *TaSdr-A1a* | *TaSdr-B1b* | *Tamyb10-A1b* | *Tamyb10-B1a* | *Tamyb10-D1a* | *Vp-1Ba* | *TaDFR-Bb* | *TaMKK3-Aa* | *GS34-7Ba* | *TaMFT-3Ab* | *TaPHS1-222T* | *TaMFT-A1b* | *TaPHS1-646G* | *TaPHS1-666A* | 26.30% | 45.30% | 35.00% |
| 70 | Shixin 733 | *TaSdr-A1a* | *TaSdr-B1a* | *Tamyb10-A1a* | *Tamyb10-B1a* | *Tamyb10-D1a* | *Vp-1Ba* | *TaDFR-Ba* | *TaMKK3-Ab* | *GS34-7Ba* | *TaMFT-3Aa* | *TaPHS1-222C* | *TaMFT-A1b* | *TaPHS1-646G* | *TaPHS1-666A* | 20.70% | 34.20% | 17.80% |
| 71 | Shixin 828 | *TaSdr-A1a* | *TaSdr-B1a* | *Tamyb10-A1a* | *Tamyb10-B1a* | *Tamyb10-D1a* | *Vp-1Ba* | *TaDFR-Ba* | *TaMKK3-Ab* | *GS34-7Ba* | *TaMFT-3Aa* | *TaPHS1-222T* | *TaMFT-A1a* | *TaPHS1-646G* | *TaPHS1-666A* | 46.80% | 36.30% | 19.50% |
| 72 | Shiyou 20 | *TaSdr-A1a* | *TaSdr-B1b* | *Tamyb10-A1a* | *Tamyb10-B1a* | *Tamyb10-D1a* | *Vp-1Bc* | *TaDFR-Ba* | *TaMKK3-Ab* | *GS34-7Ba* | *TaMFT-3Ab* | *TaPHS1-222T* | *TaMFT-A1a* | *TaPHS1-646G* | *TaPHS1-666A* | 77.00% | 75.20% | 42.20% |
| 73 | XiaoYan 81 | *TaSdr-A1a* | *TaSdr-B1a* | *Tamyb10-A1a* | *Tamyb10-B1a* | *Tamyb10-D1a* | *Vp-1Bc* | *TaDFR-Bb* | *TaMKK3-Aa* | *GS34-7Ba* | *TaMFT-3Aa* | *TaPHS1-222T* | *TaMFT-A1b* | *TaPHS1-646G* | *TaPHS1-666A* | 59.00% | 56.00% | 19.20% |
| 74 | Xingmai 13 | *TaSdr-A1a* | *TaSdr-B1a* | *Tamyb10-A1a* | *Tamyb10-B1a* | *Tamyb10-D1a* | *Vp-1Ba* | *TaDFR-Bb* | *TaMKK3-Aa* | *GS34-7Ba* | *TaMFT-3Ab* | *TaPHS1-222T* | *TaMFT-A1a* | *TaPHS1-646A* | *TaPHS1-666T* | 89.80% | 90.80% | 82.70% |
| 75 | Xingmai 4 | *TaSdr-A1a* | *TaSdr-B1b* | *Tamyb10-A1a* | *Tamyb10-B1a* | *Tamyb10-D1a* | *Vp-1Ba* | *TaDFR-Bb* | *TaMKK3-Aa* | *GS34-7Ba* | *TaMFT-3Ab* | *TaPHS1-222T* | *TaMFT-A1b* | *TaPHS1-646G* | *TaPHS1-666A* | 33.80% | 65.00% | 61.80% |
| 76 | Xingmai 6 | *TaSdr-A1a* | *TaSdr-B1b* | *Tamyb10-A1a* | *Tamyb10-B1a* | *Tamyb10-D1a* | *Vp-1Ba* | *TaDFR-Bb* | *TaMKK3-Aa* | *GS34-7Ba* | *TaMFT-3Ab* | *TaPHS1-222T* | *TaMFT-A1a* | *TaPHS1-646G* | *TaPHS1-666T* | 80.70% | 85.70% | 65.30% |
| 77 | Yingbo 700 | *TaSdr-A1a* | *TaSdr-B1b* | *Tamyb10-A1a* | *Tamyb10-B1a* | *Tamyb10-D1a* | *Vp-1Ba* | *TaDFR-Bb* | *TaMKK3-Aa* | *GS34-7Ba* | *TaMFT-3Aa* | *TaPHS1-222T* | *TaMFT-A1b* | *TaPHS1-646G* | *TaPHS1-666A* | 40.60% | 61.10% | 26.80% |
| 78 | Zhongmai 155 | *TaSdr-A1a* | *TaSdr-B1b* | *Tamyb10-A1a* | *Tamyb10-B1a* | *Tamyb10-D1a* | *Vp-1Ba* | *TaDFR-Bb* | *TaMKK3-Aa* | *GS34-7Ba* | *TaMFT-3Aa* | *TaPHS1-222T* | *TaMFT-A1a* | *TaPHS1-646G* | *TaPHS1-666A* | 41.00% | 60.30% | 45.10% |
| 79 | Zhongxinmai 9 | *TaSdr-A1a* | *TaSdr-B1b* | *Tamyb10-A1a* | *Tamyb10-B1a* | *Tamyb10-D1a* | *Vp-1Bc* | *TaDFR-Bb* | *TaMKK3-Aa* | *GS34-7Ba* | *TaMFT-3Aa* | *TaPHS1-222T* | *TaMFT-A1a* | *TaPHS1-646G* | *TaPHS1-666A* | 39.60% | 73.60% | 42.30% |
| 80 | Jimai 19 | *TaSdr-A1a* | *TaSdr-B1b* | *Tamyb10-A1a* | *Tamyb10-B1a* | *Tamyb10-D1a* | *Vp-1Ba* | *TaDFR-Bb* | *TaMKK3-Aa* | *GS34-7Ba* | *TaMFT-3Aa* | *TaPHS1-222T* | *TaMFT-A1b* | *TaPHS1-646G* | *TaPHS1-666A* | 19.30% | 50.40% | 13.70% |
| 81 | Jimai 20 | *TaSdr-A1a* | *TaSdr-B1b* | *Tamyb10-A1a* | *Tamyb10-B1a* | *Tamyb10-D1a* | *Vp-1Ba* | *TaDFR-Bb* | *TaMKK3-Aa* | *GS34-7Ba* | *TaMFT-3Aa* | *TaPHS1-222T* | *TaMFT-A1a* | *TaPHS1-646G* | *TaPHS1-666A* | 27.50% | 38.90% | 19.70% |
| 82 | Jimai 21 | *TaSdr-A1a* | *TaSdr-B1b* | *Tamyb10-A1a* | *Tamyb10-B1a* | *Tamyb10-D1a* | *Vp-1Bc* | *TaDFR-Bb* | *TaMKK3-Aa* | *GS34-7Ba* | *TaMFT-3Aa* | *TaPHS1-222T* | *TaMFT-A1a* | *TaPHS1-646G* | *TaPHS1-666A* | 38.40% | 63.00% | 26.90% |
| 83 | Jimai 22 | *TaSdr-A1a* | *TaSdr-B1b* | *Tamyb10-A1a* | *Tamyb10-B1a* | *Tamyb10-D1a* | *Vp-1Ba* | *TaDFR-Bb* | *TaMKK3-Aa* | *GS34-7Ba* | *TaMFT-3Aa* | *TaPHS1-222T* | *TaMFT-A1a* | *TaPHS1-646G* | *TaPHS1-666A* | 52.70% | 41.60% | 19.60% |
| 84 | Jinan 13 | *TaSdr-A1a* | *TaSdr-B1b* | *Tamyb10-A1a* | *Tamyb10-B1b* | *Tamyb10-D1a* | *Vp-1Ba* | *TaDFR-Bb* | *TaMKK3-Ab* | *GS34-7Ba* | *TaMFT-3Aa* | *TaPHS1-222T* | *TaMFT-A1a* | *TaPHS1-646G* | *TaPHS1-666A* | 47.70% | 54.70% | 25.60% |
| 85 | Jinan 16 | *TaSdr-A1a* | *TaSdr-B1b* | *Tamyb10-A1a* | *Tamyb10-B1a* | *Tamyb10-D1a* | *Vp-1Bb* | *TaDFR-Bb* | *TaMKK3-Aa* | *GS34-7Ba* | *TaMFT-3Aa* | *TaPHS1-222T* | *TaMFT-A1b* | *TaPHS1-646G* | *TaPHS1-666A* | 30.10% | 52.60% | 24.10% |
| 86 | Jinan 17 | *TaSdr-A1a* | *TaSdr-B1b* | *Tamyb10-A1a* | *Tamyb10-B1a* | *Tamyb10-D1a* | *Vp-1Ba* | *TaDFR-Bb* | *TaMKK3-Aa* | *GS34-7Ba* | *TaMFT-3Ab* | *TaPHS1-222T* | *TaMFT-A1a* | *TaPHS1-646G* | *TaPHS1-666A* | 51.00% | 62.30% | 40.20% |
| 87 | Liangxing 66 | *TaSdr-A1a* | *TaSdr-B1b* | *Tamyb10-A1a* | *Tamyb10-B1a* | *Tamyb10-D1a* | *Vp-1Bc* | *TaDFR-Bb* | *TaMKK3-Aa* | *GS34-7Ba* | *TaMFT-3Aa* | *TaPHS1-222T* | *TaMFT-A1a* | *TaPHS1-646G* | *TaPHS1-666A* | 40.50% | 64.20% | 32.40% |
| 88 | Liangxing 99 | *TaSdr-A1a* | *TaSdr-B1b* | *Tamyb10-A1a* | *Tamyb10-B1a* | *Tamyb10-D1a* | *Vp-1Ba* | *TaDFR-Bb* | *TaMKK3-Aa* | *GS34-7Ba* | *TaMFT-3Aa* | *TaPHS1-222T* | *TaMFT-A1a* | *TaPHS1-646G* | *TaPHS1-666A* | 47.20% | 44.30% | 35.60% |
| 89 | Linmai 4 | *TaSdr-A1b* | *TaSdr-B1b* | *Tamyb10-A1a* | *Tamyb10-B1a* | *Tamyb10-D1a* | *Vp-1Ba* | *TaDFR-Bb* | *TaMKK3-Aa* | *GS34-7Ba* | *TaMFT-3Aa* | *TaPHS1-222T* | *TaMFT-A1b* | *TaPHS1-646G* | *TaPHS1-666A* | 42.50% | 69.00% | 26.90% |
| 90 | Lukenmai 9 | *TaSdr-A1a* | *TaSdr-B1b* | *Tamyb10-A1a* | *Tamyb10-B1a* | *Tamyb10-D1a* | *Vp-1Bc* | *TaDFR-Ba* | *TaMKK3-Ab* | *GS34-7Ba* | *TaMFT-3Ab* | *TaPHS1-222T* | *TaMFT-A1a* | *TaPHS1-646A* | *TaPHS1-666T* | 59.60% | 57.00% | 41.40% |
| 91 | Lumai 1 | *TaSdr-A1a* | *TaSdr-B1b* | *Tamyb10-A1a* | *Tamyb10-B1a* | *Tamyb10-D1a* | *Vp-1Ba* | *TaDFR-Bb* | *TaMKK3-Aa* | *GS34-7Ba* | *TaMFT-3Aa* | *TaPHS1-222T* | *TaMFT-A1b* | *TaPHS1-646A* | *TaPHS1-666T* | 51.90% | 60.20% | 44.60% |
| 92 | Lumai 13 | *TaSdr-A1a* | *TaSdr-B1b* | *Tamyb10-A1a* | *Tamyb10-B1a* | *Tamyb10-D1a* | *Vp-1Ba* | *TaDFR-Bb* | *TaMKK3-Aa* | *GS34-7Ba* | *TaMFT-3Aa* | *TaPHS1-222T* | *TaMFT-A1b* | *TaPHS1-646G* | *TaPHS1-666A* | 55.70% | 68.00% | 50.90% |
| 93 | Lumai 14 | *TaSdr-A1a* | *TaSdr-B1b* | *Tamyb10-A1a* | *Tamyb10-B1a* | *Tamyb10-D1a* | *Vp-1Ba* | *TaDFR-Bb* | *TaMKK3-Ab* | *GS34-7Ba* | *TaMFT-3Aa* | *TaPHS1-222T* | *TaMFT-A1a* | *TaPHS1-646G* | *TaPHS1-666A* | 23.00% | 58.80% | 17.00% |
| 94 | Lumai 15 | *TaSdr-A1a* | *TaSdr-B1b* | *Tamyb10-A1a* | *Tamyb10-B1a* | *Tamyb10-D1a* | *Vp-1Bc* | *TaDFR-Bb* | *TaMKK3-Aa* | *GS34-7Ba* | *TaMFT-3Aa* | *TaPHS1-222T* | *TaMFT-A1a* | *TaPHS1-646G* | *TaPHS1-666A* | 12.60% | 43.10% | 21.80% |
| 95 | Lumai 21 | *TaSdr-A1a* | *TaSdr-B1b* | *Tamyb10-A1a* | *Tamyb10-B1a* | *Tamyb10-D1a* | *Vp-1Bc* | *TaDFR-Bb* | *TaMKK3-Aa* | *GS34-7Ba* | *TaMFT-3Aa* | *TaPHS1-222T* | *TaMFT-A1a* | *TaPHS1-646G* | *TaPHS1-666A* | 42.20% | 55.00% | 30.90% |
| 96 | Lumai 22 | *TaSdr-A1a* | *TaSdr-B1b* | *Tamyb10-A1a* | *Tamyb10-B1a* | *Tamyb10-D1a* | *Vp-1Bc* | *TaDFR-Bb* | *TaMKK3-Aa* | *GS34-7Ba* | *TaMFT-3Aa* | *TaPHS1-222T* | *TaMFT-A1a* | *TaPHS1-646A* | *TaPHS1-666T* | 54.20% | 68.50% | 53.10% |
| 97 | Lumai 23 | *TaSdr-A1a* | *TaSdr-B1b* | *Tamyb10-A1a* | *Tamyb10-B1a* | *Tamyb10-D1a* | *Vp-1Ba* | *TaDFR-Bb* | *TaMKK3-Aa* | *GS34-7Ba* | *TaMFT-3Aa* | *TaPHS1-222T* | *TaMFT-A1b* | *TaPHS1-646G* | *TaPHS1-666A* | 77.80% | 71.00% | 58.50% |
| 98 | Lumai 7 | *TaSdr-A1a* | *TaSdr-B1a* | *Tamyb10-A1a* | *Tamyb10-B1a* | *Tamyb10-D1a* | *Vp-1Ba* | *TaDFR-Ba* | *TaMKK3-Aa* | *GS34-7Ba* | *TaMFT-3Aa* | *TaPHS1-222T* | *TaMFT-A1b* | *TaPHS1-646A* | *TaPHS1-666T* | 33.70% | 43.60% | 11.20% |
| 99 | Luyuan 502 | *TaSdr-A1a* | *TaSdr-B1b* | *Tamyb10-A1a* | *Tamyb10-B1a* | *Tamyb10-D1a* | *Vp-1Bc* | *TaDFR-Bb* | *TaMKK3-Aa* | *GS34-7Ba* | *TaMFT-3Aa* | *TaPHS1-222T* | *TaMFT-A1b* | *TaPHS1-646G* | *TaPHS1-666A* | 44.70% | 43.40% | 15.00% |
| 100 | Qimai 2 | *TaSdr-A1a* | *TaSdr-B1b* | *Tamyb10-A1a* | *Tamyb10-B1a* | *Tamyb10-D1a* | *Vp-1Bc* | *TaDFR-Bb* | *TaMKK3-Aa* | *GS34-7Ba* | *TaMFT-3Aa* | *TaPHS1-222T* | *TaMFT-A1a* | *TaPHS1-646G* | *TaPHS1-666A* | 38.90% | 68.70% | 26.80% |
| 101 | Qingfeng 1 | *TaSdr-A1a* | *TaSdr-B1b* | *Tamyb10-A1a* | *Tamyb10-B1a* | *Tamyb10-D1a* | *Vp-1Bb* | *TaDFR-Ba* | *TaMKK3-Aa* | *GS34-7Ba* | *TaMFT-3Aa* | *TaPHS1-222T* | *TaMFT-A1a* | *TaPHS1-646G* | *TaPHS1-666A* | 24.30% | 56.20% | 12.60% |
| 102 | Qingnong 2 | *TaSdr-A1a* | *TaSdr-B1b* | *Tamyb10-A1a* | *Tamyb10-B1a* | *Tamyb10-D1a* | *Vp-1Bb* | *TaDFR-Bb* | *TaMKK3-Aa* | *GS34-7Ba* | *TaMFT-3Aa* | *TaPHS1-222T* | *TaMFT-A1a* | *TaPHS1-646G* | *TaPHS1-666A* | 34.80% | 51.00% | 25.20% |
| 103 | Runong 14 | *TaSdr-A1a* | *TaSdr-B1b* | *Tamyb10-A1a* | *Tamyb10-B1a* | *Tamyb10-D1a* | *Vp-1Bd* | *TaDFR-Bb* | *TaMKK3-Aa* | *GS34-7Ba* | *TaMFT-3Aa* | *TaPHS1-222T* | *TaMFT-A1a* | *TaPHS1-646G* | *TaPHS1-666A* | 32.40% | 39.30% | 30.80% |
| 104 | Shannong 17 | *TaSdr-A1a* | *TaSdr-B1b* | *Tamyb10-A1a* | *Tamyb10-B1a* | *Tamyb10-D1a* | *Vp-1Bc* | *TaDFR-Bb* | *TaMKK3-Ab* | *GS34-7Ba* | *TaMFT-3Ab* | *TaPHS1-222T* | *TaMFT-A1a* | *TaPHS1-646A* | *TaPHS1-666T* | 47.30% | 63.30% | 26.30% |
| 105 | Shannong 19 | *TaSdr-A1a* | *TaSdr-B1b* | *Tamyb10-A1a* | *Tamyb10-B1a* | *Tamyb10-D1a* | *Vp-1Ba* | *TaDFR-Bb* | *TaMKK3-Ab* | *GS34-7Ba* | *TaMFT-3Aa* | *TaPHS1-222T* | *TaMFT-A1a* | *TaPHS1-646A* | *TaPHS1-666T* | 60.60% | 68.80% | 57.60% |
| 106 | Shannong 20 | *TaSdr-A1a* | *TaSdr-B1b* | *Tamyb10-A1a* | *Tamyb10-B1a* | *Tamyb10-D1a* | *Vp-1Ba* | *TaDFR-Bb* | *TaMKK3-Aa* | *GS34-7Ba* | *TaMFT-3Aa* | *TaPHS1-222T* | *TaMFT-A1a* | *TaPHS1-646G* | *TaPHS1-666A* | 47.60% | 49.00% | 45.00% |
| 107 | Shannong 22 | *TaSdr-A1a* | *TaSdr-B1b* | *Tamyb10-A1a* | *Tamyb10-B1a* | *Tamyb10-D1a* | *Vp-1Ba* | *TaDFR-Bb* | *TaMKK3-Aa* | *GS34-7Ba* | *TaMFT-3Aa* | *TaPHS1-222T* | *TaMFT-A1a* | *TaPHS1-646G* | *TaPHS1-666A* | 35.20% | 52.10% | 29.60% |
| 108 | Shannong 24 | *TaSdr-A1a* | *TaSdr-B1b* | *Tamyb10-A1a* | *Tamyb10-B1a* | *Tamyb10-D1a* | *Vp-1Bc* | *TaDFR-Bb* | *TaMKK3-Aa* | *GS34-7Ba* | *TaMFT-3Aa* | *TaPHS1-222T* | *TaMFT-A1a* | *TaPHS1-646G* | *TaPHS1-666A* | 40.40% | 71.30% | 36.80% |
| 109 | Shannong 28 | *TaSdr-A1a* | *TaSdr-B1b* | *Tamyb10-A1a* | *Tamyb10-B1a* | *Tamyb10-D1a* | *Vp-1Bc* | *TaDFR-Bb* | *TaMKK3-Ab* | *GS34-7Ba* | *TaMFT-3Aa* | *TaPHS1-222T* | *TaMFT-A1a* | *TaPHS1-646G* | *TaPHS1-666A* | 50.90% | 63.30% | 31.40% |
| 110 | Shannong 30 | *TaSdr-A1a* | *TaSdr-B1b* | *Tamyb10-A1a* | *Tamyb10-B1a* | *Tamyb10-D1a* | *Vp-1Bc* | *TaDFR-Bb* | *TaMKK3-Ab* | *GS34-7Ba* | *TaMFT-3Aa* | *TaPHS1-222T* | *TaMFT-A1a* | *TaPHS1-646G* | *TaPHS1-666A* | 40.60% | 65.10% | 28.30% |
| 111 | Shannong 32 | *TaSdr-A1a* | *TaSdr-B1b* | *Tamyb10-A1a* | *Tamyb10-B1a* | *Tamyb10-D1a* | *Vp-1Bc* | *TaDFR-Bb* | *TaMKK3-Aa* | *GS34-7Ba* | *TaMFT-3Aa* | *TaPHS1-222T* | *TaMFT-A1a* | *TaPHS1-646G* | *TaPHS1-666A* | 26.00% | 46.70% | 18.10% |
| 112 | Shannongfu 63 | *TaSdr-A1a* | *TaSdr-B1b* | *Tamyb10-A1a* | *Tamyb10-B1a* | *Tamyb10-D1a* | *Vp-1Ba* | *TaDFR-Bb* | *—* | *GS34-7Ba* | *TaMFT-3Ab* | *TaPHS1-222T* | *TaMFT-A1a* | *TaPHS1-646A* | *TaPHS1-666T* | 42.10% | 61.30% | 42.70% |
| 113 | Tainong 18 | *TaSdr-A1a* | *TaSdr-B1b* | *Tamyb10-A1a* | *Tamyb10-B1a* | *Tamyb10-D1a* | *Vp-1Bc* | *TaDFR-Ba* | *TaMKK3-Ab* | *GS34-7Ba* | *TaMFT-3Aa* | *TaPHS1-222T* | *TaMFT-A1a* | *TaPHS1-646G* | *TaPHS1-666A* | 60.90% | 72.30% | 54.50% |
| 114 | Taishan 1 | *TaSdr-A1a* | *TaSdr-B1b* | *Tamyb10-A1a* | *Tamyb10-B1a* | *Tamyb10-D1a* | *Vp-1Bb* | *TaDFR-Bb* | *TaMKK3-Aa* | *GS34-7Ba* | *TaMFT-3Aa* | *TaPHS1-222T* | *TaMFT-A1a* | *TaPHS1-646A* | *TaPHS1-666T* | 79.20% | 89.20% | 64.70% |
| 115 | Taishan 21 | *TaSdr-A1a* | *TaSdr-B1b* | *Tamyb10-A1a* | *Tamyb10-B1a* | *Tamyb10-D1a* | *Vp-1Bc* | *TaDFR-Ba* | *TaMKK3-Ab* | *GS34-7Ba* | *TaMFT-3Ab* | *TaPHS1-222T* | *TaMFT-A1a* | *TaPHS1-646A* | *TaPHS1-666T* | 58.90% | 72.00% | 54.00% |
| 116 | Taishan 23 | *TaSdr-A1a* | *TaSdr-B1b* | *Tamyb10-A1a* | *Tamyb10-B1a* | *Tamyb10-D1a* | *Vp-1Ba* | *TaDFR-Bb* | *TaMKK3-Aa* | *GS34-7Ba* | *TaMFT-3Aa* | *TaPHS1-222T* | *TaMFT-A1a* | *TaPHS1-646G* | *TaPHS1-666A* | 61.30% | 72.20% | 54.20% |
| 117 | Weimai 8 | *TaSdr-A1a* | *TaSdr-B1b* | *Tamyb10-A1a* | *Tamyb10-B1a* | *Tamyb10-D1a* | *Vp-1Bc* | *TaDFR-Bb* | *TaMKK3-Aa* | *GS34-7Ba* | *TaMFT-3Aa* | *TaPHS1-222T* | *TaMFT-A1b* | *TaPHS1-646G* | *TaPHS1-666A* | 58.90% | 71.10% | 53.50% |
| 118 | Xinmai 296 | *TaSdr-A1a* | *TaSdr-B1b* | *Tamyb10-A1a* | *Tamyb10-B1a* | *Tamyb10-D1a* | *Vp-1Ba* | *TaDFR-Ba* | *TaMKK3-Aa* | *GS34-7Ba* | *TaMFT-3Aa* | *TaPHS1-222T* | *TaMFT-A1a* | *TaPHS1-646G* | *TaPHS1-666A* | 63.20% | 60.10% | 26.70% |
| 119 | Yanfu 188 | *TaSdr-A1a* | *TaSdr-B1b* | *Tamyb10-A1a* | *Tamyb10-B1a* | *Tamyb10-D1a* | *Vp-1Ba* | *TaDFR-Ba* | *TaMKK3-Ab* | *GS34-7Ba* | *TaMFT-3Ab* | *TaPHS1-222T* | *TaMFT-A1a* | *TaPHS1-646G* | *TaPHS1-666A* | 53.20% | 51.50% | 34.00% |
| 120 | Yanmai 98 | *TaSdr-A1a* | *TaSdr-B1a* | *Tamyb10-A1a* | *Tamyb10-B1a* | *Tamyb10-D1a* | *Vp-1Bc* | *TaDFR-Ba* | *TaMKK3-Aa* | *GS34-7Ba* | *TaMFT-3Aa* | *TaPHS1-222T* | *TaMFT-A1a* | *TaPHS1-646A* | *TaPHS1-666T* | 71.60% | 67.70% | 46.00% |
| 121 | Yannong 15 | *TaSdr-A1a* | *TaSdr-B1b* | *Tamyb10-A1a* | *Tamyb10-B1a* | *Tamyb10-D1a* | *Vp-1Bc* | *TaDFR-Bb* | *TaMKK3-Aa* | *GS34-7Ba* | *TaMFT-3Aa* | *TaPHS1-222T* | *TaMFT-A1a* | *TaPHS1-646G* | *TaPHS1-666A* | 43.40% | 46.60% | 22.60% |
| 122 | Yannong 19 | *TaSdr-A1a* | *TaSdr-B1b* | *Tamyb10-A1a* | *Tamyb10-B1a* | *Tamyb10-D1a* | *Vp-1Bc* | *TaDFR-Ba* | *TaMKK3-Ab* | *GS34-7Ba* | *TaMFT-3Aa* | *TaPHS1-222T* | *TaMFT-A1a* | *TaPHS1-646G* | *TaPHS1-666A* | 41.60% | 50.50% | 25.60% |
| 123 | Yannong 21 | *TaSdr-A1a* | *TaSdr-B1b* | *Tamyb10-A1a* | *Tamyb10-B1a* | *Tamyb10-D1a* | *Vp-1Bc* | *TaDFR-Bb* | *TaMKK3-Ab* | *GS34-7Ba* | *TaMFT-3Aa* | *TaPHS1-222T* | *TaMFT-A1a* | *TaPHS1-646G* | *TaPHS1-666A* | 43.60% | 52.60% | 22.90% |
| 124 | Yannong 24 | *TaSdr-A1a* | *TaSdr-B1b* | *Tamyb10-A1a* | *Tamyb10-B1a* | *Tamyb10-D1a* | *Vp-1Bc* | *TaDFR-Bb* | *TaMKK3-Aa* | *GS34-7Ba* | *TaMFT-3Aa* | *TaPHS1-222T* | *TaMFT-A1a* | *TaPHS1-646G* | *TaPHS1-666A* | 39.80% | 31.10% | 16.30% |
| 125 | Yannong 5286 | *TaSdr-A1a* | *TaSdr-B1b* | *Tamyb10-A1a* | *Tamyb10-B1a* | *Tamyb10-D1a* | *Vp-1Bc* | *TaDFR-Bb* | *TaMKK3-Aa* | *GS34-7Ba* | *TaMFT-3Aa* | *TaPHS1-222T* | *TaMFT-A1a* | *TaPHS1-646G* | *TaPHS1-666A* | 44.80% | 49.60% | 34.10% |
| 126 | Yannong 836 | *TaSdr-A1a* | *TaSdr-B1b* | *Tamyb10-A1a* | *Tamyb10-B1a* | *Tamyb10-D1a* | *Vp-1Bc* | *TaDFR-Bb* | *TaMKK3-Aa* | *GS34-7Ba* | *TaMFT-3Aa* | *TaPHS1-222T* | *TaMFT-A1b* | *TaPHS1-646G* | *TaPHS1-666A* | 24.50% | 41.70% | 19.70% |
| 127 | Yannong 999 | *TaSdr-A1a* | *TaSdr-B1b* | *Tamyb10-A1a* | *Tamyb10-B1a* | *Tamyb10-D1a* | *Vp-1Ba* | *TaDFR-Bb* | *TaMKK3-Aa* | *GS34-7Ba* | *TaMFT-3Aa* | *TaPHS1-222T* | *TaMFT-A1b* | *TaPHS1-646G* | *TaPHS1-666A* | 45.80% | 51.00% | 48.50% |
| 128 | Zhouyuan 9369 | *TaSdr-A1a* | *TaSdr-B1b* | *Tamyb10-A1a* | *Tamyb10-B1a* | *Tamyb10-D1a* | *Vp-1Bc* | *TaDFR-Bb* | *TaMKK3-Aa* | *GS34-7Ba* | *TaMFT-3Aa* | *TaPHS1-222T* | *TaMFT-A1b* | *TaPHS1-646G* | *TaPHS1-666A* | 55.10% | 56.70% | 27.40% |
| 129 | Zimai 12 | *TaSdr-A1a* | *TaSdr-B1b* | *Tamyb10-A1a* | *Tamyb10-B1a* | *Tamyb10-D1a* | *Vp-1Bc* | *TaDFR-Ba* | *TaMKK3-Ab* | *GS34-7Bb* | *TaMFT-3Aa* | *TaPHS1-222T* | *TaMFT-A1b* | *TaPHS1-646G* | *TaPHS1-666A* | 51.80% | 72.30% | 60.00% |
| 130 | Shunmai 1718 | *TaSdr-A1a* | *TaSdr-B1b* | *Tamyb10-A1a* | *Tamyb10-B1a* | *Tamyb10-D1a* | *Vp-1Bc* | *TaDFR-Bb* | *TaMKK3-Aa* | *GS34-7Ba* | *TaMFT-3Ab* | *TaPHS1-222T* | *TaMFT-A1a* | *TaPHS1-646G* | *TaPHS1-666A* | 45.90% | 64.10% | 31.00% |
| 131 | Yaomai 16 | *TaSdr-A1a* | *TaSdr-B1b* | *Tamyb10-A1a* | *Tamyb10-B1a* | *Tamyb10-D1a* | *Vp-1Bc* | *TaDFR-Bb* | *TaMKK3-Aa* | *GS34-7Ba* | *TaMFT-3Aa* | *TaPHS1-222T* | *TaMFT-A1b* | *TaPHS1-646G* | *TaPHS1-666A* | 53.00% | 80.40% | 54.20% |
| 132 | Yunhan 618 | *TaSdr-A1a* | *TaSdr-B1a* | *Tamyb10-A1a* | *Tamyb10-B1a* | *Tamyb10-D1a* | *Vp-1Bc* | *TaDFR-Bb* | *TaMKK3-Ab* | *GS34-7Ba* | *TaMFT-3Aa* | *TaPHS1-222T* | *TaMFT-A1b* | *TaPHS1-646G* | *TaPHS1-666A* | 33.40% | 52.70% | 26.70% |
| 133 | 04Zhong 36 | *TaSdr-A1a* | *TaSdr-B1b* | *Tamyb10-A1b* | *Tamyb10-B1b* | *Tamyb10-D1a* | *Vp-1Bc* | *TaDFR-Bb* | *TaMKK3-Aa* | *GS34-7Ba* | *TaMFT-3Aa* | *TaPHS1-222T* | *TaMFT-A1b* | *TaPHS1-646G* | *TaPHS1-666A* | 31.10% | 31.30% | 16.00% |
| 134 | Aikang 58 | *TaSdr-A1b* | *TaSdr-B1b* | *Tamyb10-A1a* | *Tamyb10-B1a* | *Tamyb10-D1a* | *Vp-1Ba* | *TaDFR-Bb* | *TaMKK3-Ab* | *GS34-7Ba* | *TaMFT-3Ab* | *TaPHS1-222T* | *TaMFT-A1a* | *TaPHS1-646A* | *TaPHS1-666T* | 34.60% | 48.70% | 17.70% |
| 135 | Bainong 160 | *TaSdr-A1a* | *TaSdr-B1b* | *Tamyb10-A1a* | *Tamyb10-B1a* | *Tamyb10-D1a* | *Vp-1Bc* | *TaDFR-Ba* | *TaMKK3-Aa* | *GS34-7Bb* | *TaMFT-3Aa* | *TaPHS1-222T* | *TaMFT-A1b* | *TaPHS1-646G* | *TaPHS1-666A* | 24.00% | 34.00% | 14.70% |
| 136 | Bainong 207 | *TaSdr-A1a* | *TaSdr-B1b* | *Tamyb10-A1a* | *Tamyb10-B1a* | *Tamyb10-D1a* | *Vp-1Ba* | *TaDFR-Ba* | *TaMKK3-Aa* | *GS34-7Ba* | *TaMFT-3Aa* | *TaPHS1-222T* | *TaMFT-A1b* | *TaPHS1-646G* | *TaPHS1-666A* | 35.40% | 59.40% | 25.10% |
| 137 | Bainong 3217 | *TaSdr-A1a* | *TaSdr-B1a* | *Tamyb10-A1a* | *Tamyb10-B1a* | *Tamyb10-D1a* | *Vp-1Ba* | *TaDFR-Bb* | *TaMKK3-Ab* | *GS34-7Ba* | *TaMFT-3Aa* | *TaPHS1-222C* | *TaMFT-A1a* | *TaPHS1-646G* | *TaPHS1-666A* | 12.30% | 32.70% | 5.90% |
| 138 | Bainong 418 | *TaSdr-A1a* | *TaSdr-B1b* | *Tamyb10-A1a* | *Tamyb10-B1a* | *Tamyb10-D1a* | *Vp-1Ba* | *TaDFR-Bb* | *TaMKK3-Ab* | *GS34-7Ba* | *TaMFT-3Aa* | *TaPHS1-222T* | *TaMFT-A1b* | *TaPHS1-646G* | *TaPHS1-666A* | 46.60% | 54.60% | 19.60% |
| 139 | Baiquan 3039 | *TaSdr-A1a* | *TaSdr-B1a* | *Tamyb10-A1a* | *Tamyb10-B1a* | *Tamyb10-D1a* | *Vp-1Ba* | *TaDFR-Ba* | *TaMKK3-Aa* | *GS34-7Ba* | *TaMFT-3Aa* | *TaPHS1-222C* | *TaMFT-A1b* | *TaPHS1-646G* | *TaPHS1-666A* | 13.70% | 23.30% | 3.00% |
| 140 | Bonong 7023 | *TaSdr-A1a* | *TaSdr-B1b* | *Tamyb10-A1a* | *Tamyb10-B1a* | *Tamyb10-D1b* | *Vp-1Ba* | *TaDFR-Bb* | *TaMKK3-Ab* | *GS34-7Ba* | *TaMFT-3Aa* | *TaPHS1-222T* | *TaMFT-A1b* | *TaPHS1-646A* | *TaPHS1-666T* | 30.10% | 31.90% | 7.40% |
| 141 | Fanmai 5 | *TaSdr-A1a* | *TaSdr-B1b* | *Tamyb10-A1a* | *Tamyb10-B1a* | *Tamyb10-D1a* | *Vp-1Bc* | *TaDFR-Bb* | *TaMKK3-Aa* | *GS34-7Ba* | *TaMFT-3Aa* | *TaPHS1-222C* | *TaMFT-A1b* | *TaPHS1-646G* | *TaPHS1-666A* | 25.00% | 37.90% | 14.50% |
| 142 | Fanmai 8 | *TaSdr-A1a* | *TaSdr-B1a* | *Tamyb10-A1a* | *Tamyb10-B1a* | *Tamyb10-D1a* | *Vp-1Ba* | *TaDFR-Bb* | *TaMKK3-Aa* | *GS34-7Ba* | *TaMFT-3Aa* | *TaPHS1-222T* | *TaMFT-A1b* | *TaPHS1-646G* | *TaPHS1-666A* | 19.00% | 28.90% | 10.80% |
| 143 | Fengdecunmai 1 | *TaSdr-A1b* | *TaSdr-B1b* | *Tamyb10-A1a* | *Tamyb10-B1a* | *Tamyb10-D1a* | *Vp-1Ba* | *TaDFR-Bb* | *TaMKK3-Ab* | *GS34-7Ba* | *TaMFT-3Ab* | *TaPHS1-222T* | *TaMFT-A1a* | *TaPHS1-646A* | *TaPHS1-666T* | 66.00% | 75.70% | 21.40% |
| 144 | Fengwu 981 | *TaSdr-A1a* | *TaSdr-B1b* | *Tamyb10-A1a* | *Tamyb10-B1a* | *Tamyb10-D1a* | *Vp-1Ba* | *TaDFR-Bb* | *TaMKK3-Ab* | *GS34-7Ba* | *TaMFT-3Ab* | *TaPHS1-222T* | *TaMFT-A1a* | *—* | *TaPHS1-666T* | 55.50% | 76.90% | 14.80% |
| 145 | Fumai 2008 | *TaSdr-A1a* | *TaSdr-B1b* | *Tamyb10-A1a* | *Tamyb10-B1a* | *Tamyb10-D1a* | *Vp-1Ba* | *TaDFR-Bb* | *TaMKK3-Aa* | *GS34-7Ba* | *TaMFT-3Ab* | *TaPHS1-222T* | *TaMFT-A1a* | *TaPHS1-646A* | *TaPHS1-666T* | 73.00% | 85.60% | 58.60% |
| 146 | Guanmai 1 | *TaSdr-A1a* | *TaSdr-B1b* | *Tamyb10-A1a* | *Tamyb10-B1a* | *Tamyb10-D1a* | *Vp-1Ba* | *TaDFR-Bb* | *TaMKK3-Aa* | *GS34-7Ba* | *TaMFT-3Aa* | *TaPHS1-222T* | *TaMFT-A1b* | *TaPHS1-646G* | *TaPHS1-666A* | 70.70% | 78.70% | 47.90% |
| 147 | Huaichuan 916 | *TaSdr-A1a* | *TaSdr-B1a* | *Tamyb10-A1a* | *Tamyb10-B1a* | *Tamyb10-D1a* | *Vp-1Bc* | *TaDFR-Bb* | *TaMKK3-Ab* | *GS34-7Ba* | *TaMFT-3Aa* | *TaPHS1-222T* | *TaMFT-A1a* | *TaPHS1-646G* | *TaPHS1-666A* | 45.90% | 39.60% | 26.40% |
| 148 | Huapei 3 | *TaSdr-A1a* | *TaSdr-B1b* | *Tamyb10-A1a* | *Tamyb10-B1a* | *Tamyb10-D1a* | *Vp-1Ba* | *TaDFR-Ba* | *TaMKK3-Ab* | *GS34-7Ba* | *TaMFT-3Ab* | *TaPHS1-222T* | *TaMFT-A1a* | *TaPHS1-646G* | *TaPHS1-666A* | 69.20% | 67.80% | 37.90% |
| 149 | Huapei 5 | *TaSdr-A1a* | *TaSdr-B1b* | *Tamyb10-A1a* | *Tamyb10-B1a* | *Tamyb10-D1a* | *Vp-1Bc* | *TaDFR-Bb* | *TaMKK3-Ab* | *GS34-7Ba* | *TaMFT-3Ab* | *TaPHS1-222T* | *TaMFT-A1a* | *TaPHS1-646G* | *TaPHS1-666A* | 80.90% | 81.30% | 23.80% |
| 150 | Huapei 8 | *TaSdr-A1a* | *TaSdr-B1b* | *Tamyb10-A1a* | *Tamyb10-B1a* | *Tamyb10-D1a* | *Vp-1Ba* | *TaDFR-Ba* | *TaMKK3-Aa* | *GS34-7Ba* | *TaMFT-3Ab* | *TaPHS1-222T* | *TaMFT-A1a* | *TaPHS1-646A* | *TaPHS1-666T* | 85.00% | 82.60% | 51.80% |
| 151 | Junmai 99-7 | *TaSdr-A1a* | *TaSdr-B1b* | *Tamyb10-A1b* | *Tamyb10-B1a* | *Tamyb10-D1a* | *Vp-1Bc* | *TaDFR-Ba* | *TaMKK3-Aa* | *GS34-7Ba* | *TaMFT-3Ab* | *TaPHS1-222T* | *TaMFT-A1b* | *TaPHS1-646G* | *TaPHS1-666A* | 51.00% | 46.60% | 22.10% |
| 152 | Kaimai 18 | *TaSdr-A1a* | *TaSdr-B1b* | *Tamyb10-A1a* | *Tamyb10-B1a* | *Tamyb10-D1a* | *Vp-1Ba* | *TaDFR-Bb* | *TaMKK3-Ab* | *GS34-7Ba* | *TaMFT-3Aa* | *TaPHS1-222T* | *TaMFT-A1a* | *TaPHS1-646G* | *TaPHS1-666A* | 54.30% | 52.90% | 46.00% |
| 153 | Kaimai 20 | *TaSdr-A1b* | *TaSdr-B1b* | *Tamyb10-A1a* | *Tamyb10-B1a* | *Tamyb10-D1a* | *Vp-1Ba* | *TaDFR-Bb* | *TaMKK3-Aa* | *GS34-7Ba* | *TaMFT-3Aa* | *TaPHS1-222T* | *TaMFT-A1a* | *TaPHS1-646G* | *TaPHS1-666A* | 75.60% | 81.50% | 34.40% |
| 154 | Kaimai 21 | *TaSdr-A1a* | *TaSdr-B1b* | *Tamyb10-A1a* | *Tamyb10-B1a* | *Tamyb10-D1a* | *Vp-1Ba* | *TaDFR-Bb* | *TaMKK3-Ab* | *GS34-7Ba* | *TaMFT-3Aa* | *TaPHS1-222T* | *TaMFT-A1b* | *TaPHS1-646G* | *TaPHS1-666A* | 45.50% | 48.20% | 52.40% |
| 155 | Lankao 198 | *TaSdr-A1a* | *TaSdr-B1a* | *Tamyb10-A1a* | *Tamyb10-B1a* | *Tamyb10-D1a* | *Vp-1Bc* | *TaDFR-Bb* | *TaMKK3-Ab* | *GS34-7Ba* | *TaMFT-3Aa* | *TaPHS1-222T* | *TaMFT-A1b* | *TaPHS1-646G* | *TaPHS1-666A* | 53.40% | 68.70% | 26.30% |
| 156 | Luohan 11 | *TaSdr-A1a* | *TaSdr-B1b* | *Tamyb10-A1a* | *Tamyb10-B1a* | *Tamyb10-D1a* | *Vp-1Ba* | *TaDFR-Bb* | *TaMKK3-Aa* | *GS34-7Ba* | *TaMFT-3Ab* | *TaPHS1-222T* | *TaMFT-A1a* | *TaPHS1-646G* | *TaPHS1-666T* | 9.20% | 24.50% | 7.80% |
| 157 | Luohan 13 | *TaSdr-A1a* | *TaSdr-B1a* | *Tamyb10-A1a* | *Tamyb10-B1a* | *Tamyb10-D1a* | *Vp-1Bc* | *TaDFR-Ba* | *TaMKK3-Aa* | *GS34-7Ba* | *TaMFT-3Aa* | *TaPHS1-222C* | *TaMFT-A1a* | *TaPHS1-646G* | *TaPHS1-666A* | 44.00% | 50.70% | 18.30% |
| 158 | Luohan 2 | *TaSdr-A1a* | *TaSdr-B1a* | *Tamyb10-A1a* | *Tamyb10-B1a* | *Tamyb10-D1a* | *Vp-1Bc* | *TaDFR-Ba* | *TaMKK3-Ab* | *GS34-7Ba* | *TaMFT-3Aa* | *TaPHS1-222C* | *TaMFT-A1b* | *TaPHS1-646G* | *TaPHS1-666A* | 41.50% | 42.30% | 15.80% |
| 159 | Luohan 6 | *TaSdr-A1a* | *TaSdr-B1b* | *Tamyb10-A1a* | *Tamyb10-B1a* | *Tamyb10-D1a* | *Vp-1Ba* | *TaDFR-Bb* | *TaMKK3-Aa* | *GS34-7Ba* | *TaMFT-3Ab* | *TaPHS1-222T* | *TaMFT-A1a* | *TaPHS1-646G* | *TaPHS1-666T* | 48.00% | 70.50% | 37.70% |
| 160 | Luohan 7 | *TaSdr-A1a* | *TaSdr-B1b* | *Tamyb10-A1a* | *Tamyb10-B1a* | *Tamyb10-D1a* | *Vp-1Bc* | *TaDFR-Bb* | *TaMKK3-Aa* | *GS34-7Ba* | *TaMFT-3Ab* | *TaPHS1-222T* | *TaMFT-A1a* | *TaPHS1-646G* | *TaPHS1-666A* | 14.20% | 38.70% | 27.70% |
| 161 | Luomai 18 | *TaSdr-A1a* | *TaSdr-B1b* | *Tamyb10-A1a* | *Tamyb10-B1a* | *Tamyb10-D1a* | *Vp-1Ba* | *TaDFR-Bb* | *TaMKK3-Aa* | *GS34-7Ba* | *TaMFT-3Aa* | *TaPHS1-222T* | *TaMFT-A1b* | *TaPHS1-646G* | *TaPHS1-666A* | 28.70% | 46.40% | 14.60% |
| 162 | Luomai 21 | *TaSdr-A1a* | *TaSdr-B1b* | *Tamyb10-A1a* | *Tamyb10-B1a* | *Tamyb10-D1a* | *Vp-1Ba* | *TaDFR-Bb* | *TaMKK3-Aa* | *GS34-7Bb* | *TaMFT-3Aa* | *TaPHS1-222T* | *TaMFT-A1a* | *TaPHS1-646G* | *TaPHS1-666A* | 56.30% | 59.70% | 23.60% |
| 163 | Luomai 22 | *TaSdr-A1a* | *TaSdr-B1b* | *Tamyb10-A1a* | *Tamyb10-B1a* | *Tamyb10-D1a* | *Vp-1Ba* | *TaDFR-Bb* | *TaMKK3-Ab* | *GS34-7Ba* | *TaMFT-3Ab* | *TaPHS1-222T* | *TaMFT-A1a* | *TaPHS1-646A* | *TaPHS1-666T* | 43.70% | 39.50% | 21.50% |
| 164 | Luomai 23 | *TaSdr-A1a* | *TaSdr-B1b* | *Tamyb10-A1a* | *Tamyb10-B1a* | *Tamyb10-D1a* | *Vp-1Bc* | *TaDFR-Bb* | *TaMKK3-Aa* | *GS34-7Ba* | *TaMFT-3Aa* | *TaPHS1-222T* | *TaMFT-A1a* | *TaPHS1-646G* | *TaPHS1-666A* | 21.60% | 67.00% | 33.00% |
| 165 | Luomai 24 | *TaSdr-A1a* | *TaSdr-B1a* | *Tamyb10-A1a* | *Tamyb10-B1a* | *Tamyb10-D1a* | *Vp-1Ba* | *TaDFR-Bb* | *TaMKK3-Aa* | *GS34-7Ba* | *TaMFT-3Aa* | *TaPHS1-222C* | *TaMFT-A1b* | *TaPHS1-646G* | *TaPHS1-666A* | 17.90% | 23.20% | 36.30% |
| 166 | Luomai 26 | *TaSdr-A1a* | *TaSdr-B1b* | *Tamyb10-A1a* | *Tamyb10-B1a* | *Tamyb10-D1a* | *Vp-1Bc* | *TaDFR-Bb* | *TaMKK3-Aa* | *GS34-7Ba* | *TaMFT-3Ab* | *TaPHS1-222T* | *TaMFT-A1a* | *TaPHS1-646A* | *TaPHS1-666T* | 57.00% | 68.90% | 14.00% |
| 167 | Luomai 4 | *TaSdr-A1a* | *TaSdr-B1b* | *Tamyb10-A1a* | *Tamyb10-B1a* | *Tamyb10-D1a* | *Vp-1Ba* | *TaDFR-Bb* | *TaMKK3-Aa* | *GS34-7Ba* | *TaMFT-3Aa* | *TaPHS1-222T* | *TaMFT-A1b* | *TaPHS1-646G* | *TaPHS1-666A* | 14.80% | 22.20% | 9.40% |
| 168 | Luomai 8 | *TaSdr-A1a* | *TaSdr-B1b* | *Tamyb10-A1a* | *Tamyb10-B1a* | *Tamyb10-D1a* | *Vp-1Ba* | *TaDFR-Bb* | *TaMKK3-Aa* | *GS34-7Ba* | *TaMFT-3Ab* | *TaPHS1-222T* | *TaMFT-A1b* | *TaPHS1-646G* | *TaPHS1-666A* | 31.80% | 31.70% | 12.40% |
| 169 | Lunxuan 13 | *TaSdr-A1a* | *TaSdr-B1b* | *Tamyb10-A1a* | *Tamyb10-B1a* | *Tamyb10-D1a* | *Vp-1Ba* | *TaDFR-Bb* | *TaMKK3-Aa* | *GS34-7Ba* | *TaMFT-3Aa* | *TaPHS1-222T* | *TaMFT-A1a* | *TaPHS1-646G* | *TaPHS1-666A* | 88.20% | 86.10% | 70.80% |
| 170 | Pingan 6 | *TaSdr-A1a* | *TaSdr-B1a* | *Tamyb10-A1a* | *Tamyb10-B1a* | *Tamyb10-D1a* | *Vp-1Bc* | *TaDFR-Ba* | *TaMKK3-Aa* | *GS34-7Ba* | *TaMFT-3Aa* | *TaPHS1-222T* | *TaMFT-A1a* | *TaPHS1-646G* | *TaPHS1-666A* | 58.80% | 71.10% | 37.90% |
| 171 | Pingan 8 | *TaSdr-A1a* | *TaSdr-B1b* | *Tamyb10-A1a* | *Tamyb10-B1a* | *Tamyb10-D1a* | *Vp-1Bc* | *TaDFR-Ba* | *TaMKK3-Aa* | *GS34-7Ba* | *TaMFT-3Aa* | *TaPHS1-222T* | *TaMFT-A1a* | *TaPHS1-646G* | *TaPHS1-666A* | 75.00% | 84.40% | 36.50% |
| 172 | Pingmai 998 | *TaSdr-A1a* | *TaSdr-B1b* | *Tamyb10-A1a* | *Tamyb10-B1a* | *Tamyb10-D1a* | *Vp-1Bc* | *TaDFR-Bb* | *TaMKK3-Aa* | *GS34-7Ba* | *TaMFT-3Aa* | *TaPHS1-222T* | *TaMFT-A1a* | *TaPHS1-646G* | *TaPHS1-666A* | 49.00% | 56.90% | 37.30% |
| 173 | Pumai 9 | *TaSdr-A1a* | *TaSdr-B1a* | *Tamyb10-A1a* | *Tamyb10-B1a* | *Tamyb10-D1a* | *Vp-1Ba* | *TaDFR-Bb* | *TaMKK3-Aa* | *GS34-7Ba* | *TaMFT-3Aa* | *TaPHS1-222T* | *TaMFT-A1a* | *TaPHS1-646G* | *TaPHS1-666A* | 37.60% | 38.90% | 14.20% |
| 174 | Ruzhou 0319 | *TaSdr-A1a* | *TaSdr-B1b* | *Tamyb10-A1a* | *Tamyb10-B1a* | *Tamyb10-D1a* | *Vp-1Bc* | *TaDFR-Bb* | *TaMKK3-Ab* | *GS34-7Ba* | *TaMFT-3Aa* | *TaPHS1-222T* | *TaMFT-A1b* | *TaPHS1-646G* | *TaPHS1-666A* | 58.70% | 54.70% | 36.80% |
| 175 | Taikong 6 | *TaSdr-A1a* | *TaSdr-B1b* | *Tamyb10-A1a* | *Tamyb10-B1a* | *Tamyb10-D1a* | *Vp-1Bc* | *TaDFR-Bb* | *TaMKK3-Ab* | *GS34-7Ba* | *TaMFT-3Ab* | *TaPHS1-222T* | *TaMFT-A1a* | *TaPHS1-646G* | *TaPHS1-666T* | 82.70% | 74.50% | 37.90% |
| 176 | Taixue 7 | *TaSdr-A1a* | *TaSdr-B1b* | *Tamyb10-A1b* | *Tamyb10-B1a* | *Tamyb10-D1a* | *Vp-1Ba* | *TaDFR-Ba* | *TaMKK3-Aa* | *GS34-7Ba* | *TaMFT-3Ab* | *TaPHS1-222T* | *TaMFT-A1a* | *TaPHS1-646A* | *TaPHS1-666T* | 81.10% | 87.30% | 52.10% |
| 177 | Tianmin 198 | *TaSdr-A1a* | *TaSdr-B1a* | *Tamyb10-A1a* | *Tamyb10-B1a* | *Tamyb10-D1a* | *Vp-1Bc* | *TaDFR-Ba* | *TaMKK3-Ab* | *GS34-7Ba* | *TaMFT-3Ab* | *TaPHS1-222T* | *TaMFT-A1a* | *TaPHS1-646G* | *TaPHS1-666A* | 39.60% | 46.00% | 23.80% |
| 178 | Wen 9519 | *TaSdr-A1a* | *TaSdr-B1a* | *Tamyb10-A1a* | *Tamyb10-B1a* | *Tamyb10-D1a* | *Vp-1Bc* | *TaDFR-Bb* | *TaMKK3-Ab* | *GS34-7Ba* | *TaMFT-3Aa* | *TaPHS1-222T* | *TaMFT-A1b* | *TaPHS1-646G* | *TaPHS1-666A* | 53.30% | 57.00% | 35.40% |
| 179 | Wen 9629 | *TaSdr-A1a* | *TaSdr-B1b* | *Tamyb10-A1a* | *Tamyb10-B1a* | *Tamyb10-D1a* | *Vp-1Bc* | *TaDFR-Bb* | *TaMKK3-Aa* | *GS34-7Ba* | *TaMFT-3Aa* | *TaPHS1-222T* | *TaMFT-A1a* | *TaPHS1-646G* | *TaPHS1-666T* | 71.00% | 68.80% | 25.40% |
| 180 | Wenmai 19 | *TaSdr-A1a* | *TaSdr-B1a* | *Tamyb10-A1a* | *Tamyb10-B1a* | *Tamyb10-D1a* | *Vp-1Bc* | *TaDFR-Ba* | *TaMKK3-Aa* | *GS34-7Ba* | *TaMFT-3Ab* | *TaPHS1-222T* | *TaMFT-A1a* | *TaPHS1-646G* | *TaPHS1-666A* | 45.70% | 51.80% | 29.20% |
| 181 | Xinkemai 169 | *TaSdr-A1a* | *TaSdr-B1b* | *Tamyb10-A1a* | *Tamyb10-B1a* | *Tamyb10-D1a* | *Vp-1Ba* | *TaDFR-Ba* | *TaMKK3-Ab* | *GS34-7Ba* | *TaMFT-3Ab* | *TaPHS1-222T* | *TaMFT-A1a* | *TaPHS1-646G* | *TaPHS1-666A* | 48.30% | 70.30% | 41.50% |
| 182 | Xinmai 11 | *TaSdr-A1a* | *TaSdr-B1b* | *Tamyb10-A1b* | *Tamyb10-B1a* | *Tamyb10-D1a* | *Vp-1Bc* | *TaDFR-Bb* | *TaMKK3-Aa* | *GS34-7Ba* | *TaMFT-3Aa* | *TaPHS1-222C* | *TaMFT-A1b* | *TaPHS1-646G* | *TaPHS1-666A* | 29.20% | 37.10% | 7.00% |
| 183 | Xinmai 18 | *TaSdr-A1a* | *TaSdr-B1b* | *Tamyb10-A1a* | *Tamyb10-B1a* | *Tamyb10-D1a* | *Vp-1Bc* | *TaDFR-Ba* | *TaMKK3-Aa* | *GS34-7Ba* | *TaMFT-3Aa* | *TaPHS1-222T* | *TaMFT-A1b* | *TaPHS1-646G* | *TaPHS1-666A* | 42.10% | 56.90% | 25.50% |
| 184 | Xinmai 19 | *TaSdr-A1b* | *TaSdr-B1a* | *Tamyb10-A1b* | *Tamyb10-B1a* | *Tamyb10-D1b* | *Vp-1Ba* | *TaDFR-Bb* | *TaMKK3-Aa* | *GS34-7Ba* | *TaMFT-3Aa* | *TaPHS1-222T* | *TaMFT-A1a* | *TaPHS1-646G* | *TaPHS1-666A* | 38.80% | 51.90% | 32.90% |
| 185 | Xinmai 20 | *TaSdr-A1a* | *TaSdr-B1b* | *Tamyb10-A1a* | *Tamyb10-B1a* | *Tamyb10-D1a* | *Vp-1Bc* | *TaDFR-Bb* | *TaMKK3-Aa* | *GS34-7Ba* | *TaMFT-3Aa* | *TaPHS1-222T* | *TaMFT-A1b* | *TaPHS1-646G* | *TaPHS1-666A* | 51.60% | 72.60% | 31.20% |
| 186 | Xinmai 21 | *TaSdr-A1a* | *TaSdr-B1a* | *Tamyb10-A1a* | *Tamyb10-B1a* | *Tamyb10-D1a* | *Vp-1Bc* | *TaDFR-Ba* | *TaMKK3-Ab* | *GS34-7Ba* | *TaMFT-3Aa* | *TaPHS1-222T* | *TaMFT-A1a* | *TaPHS1-646G* | *TaPHS1-666A* | 43.20% | 36.10% | 14.40% |
| 187 | Xinmai 26 | *TaSdr-A1a* | *TaSdr-B1a* | *Tamyb10-A1a* | *Tamyb10-B1a* | *Tamyb10-D1a* | *Vp-1Ba* | *TaDFR-Ba* | *TaMKK3-Aa* | *GS34-7Ba* | *TaMFT-3Aa* | *TaPHS1-222T* | *TaMFT-A1a* | *TaPHS1-646G* | *TaPHS1-666A* | 33.00% | 59.00% | 28.10% |
| 188 | Xinmai 29 | *TaSdr-A1a* | *TaSdr-B1b* | *Tamyb10-A1a* | *Tamyb10-B1a* | *Tamyb10-D1a* | *Vp-1Ba* | *TaDFR-Ba* | *TaMKK3-Ab* | *GS34-7Ba* | *TaMFT-3Aa* | *TaPHS1-222T* | *TaMFT-A1b* | *TaPHS1-646G* | *TaPHS1-666A* | 46.40% | 55.50% | 38.20% |
| 189 | Xinmai 30 | *TaSdr-A1a* | *TaSdr-B1b* | *Tamyb10-A1a* | *Tamyb10-B1a* | *Tamyb10-D1a* | *Vp-1Bc* | *TaDFR-Ba* | *TaMKK3-Ab* | *GS34-7Ba* | *TaMFT-3Aa* | *TaPHS1-222T* | *TaMFT-A1b* | *TaPHS1-646A* | *TaPHS1-666A* | 65.90% | 64.70% | 29.90% |
| 190 | Xinmai 9 | *TaSdr-A1a* | *TaSdr-B1b* | *Tamyb10-A1a* | *Tamyb10-B1a* | *Tamyb10-D1a* | *Vp-1Bc* | *TaDFR-Ba* | *TaMKK3-Aa* | *GS34-7Ba* | *TaMFT-3Aa* | *TaPHS1-222T* | *TaMFT-A1a* | *TaPHS1-646G* | *TaPHS1-666A* | 26.30% | 49.20% | 30.10% |
| 191 | Xinyuan 958 | *TaSdr-A1b* | *TaSdr-B1b* | *Tamyb10-A1a* | *Tamyb10-B1a* | *Tamyb10-D1a* | *Vp-1Bc* | *TaDFR-Ba* | *TaMKK3-Ab* | *GS34-7Ba* | *TaMFT-3Ab* | *TaPHS1-222T* | *TaMFT-A1a* | *TaPHS1-646A* | *TaPHS1-666T* | 91.10% | 89.60% | 88.80% |
| 192 | Xuke 1 | *TaSdr-A1a* | *TaSdr-B1b* | *Tamyb10-A1a* | *Tamyb10-B1a* | *Tamyb10-D1a* | *Vp-1Ba* | *TaDFR-Bb* | *TaMKK3-Ab* | *GS34-7Ba* | *TaMFT-3Ab* | *TaPHS1-222T* | *TaMFT-A1a* | *TaPHS1-646A* | *TaPHS1-666T* | 79.60% | 80.20% | 57.70% |
| 193 | Xuke 316 | *TaSdr-A1a* | *TaSdr-B1b* | *Tamyb10-A1a* | *Tamyb10-B1a* | *Tamyb10-D1a* | *Vp-1Bc* | *TaDFR-Ba* | *TaMKK3-Aa* | *GS34-7Ba* | *TaMFT-3Aa* | *TaPHS1-222T* | *TaMFT-A1a* | *TaPHS1-646G* | *TaPHS1-666A* | 34.50% | 38.50% | 26.90% |
| 194 | Xuke 718 | *TaSdr-A1a* | *TaSdr-B1a* | *Tamyb10-A1a* | *Tamyb10-B1a* | *Tamyb10-D1a* | *Vp-1Bc* | *TaDFR-Ba* | *TaMKK3-Aa* | *GS34-7Ba* | *TaMFT-3Aa* | *TaPHS1-222T* | *TaMFT-A1a* | *TaPHS1-646A* | *TaPHS1-666T* | 74.20% | 87.10% | 54.20% |
| 195 | Xunong 5 | *TaSdr-A1a* | *TaSdr-B1b* | *Tamyb10-A1a* | *Tamyb10-B1a* | *Tamyb10-D1a* | *Vp-1Ba* | *TaDFR-Bb* | *TaMKK3-Aa* | *GS34-7Ba* | *TaMFT-3Aa* | *TaPHS1-222T* | *TaMFT-A1b* | *TaPHS1-646A* | *TaPHS1-666T* | 86.60% | 81.00% | 88.00% |
| 196 | Yangao 1 | *TaSdr-A1a* | *TaSdr-B1b* | *Tamyb10-A1a* | *Tamyb10-B1a* | *Tamyb10-D1a* | *Vp-1Ba* | *TaDFR-Bb* | *TaMKK3-Ab* | *GS34-7Ba* | *TaMFT-3Aa* | *TaPHS1-222T* | *TaMFT-A1b* | *TaPHS1-646G* | *TaPHS1-666A* | 72.40% | 70.80% | 38.70% |
| 197 | Yangao 21 | *TaSdr-A1a* | *TaSdr-B1b* | *Tamyb10-A1a* | *Tamyb10-B1a* | *Tamyb10-D1a* | *Vp-1Ba* | *TaDFR-Bb* | *TaMKK3-Ab* | *GS34-7Ba* | *TaMFT-3Aa* | *TaPHS1-222T* | *TaMFT-A1a* | *TaPHS1-646G* | *TaPHS1-666A* | 61.60% | 81.00% | 50.30% |
| 198 | Yanzhan 1 | *TaSdr-A1a* | *TaSdr-B1a* | *Tamyb10-A1a* | *Tamyb10-B1a* | *Tamyb10-D1a* | *Vp-1Bc* | *TaDFR-Bb* | *TaMKK3-Aa* | *GS34-7Ba* | *TaMFT-3Aa* | *TaPHS1-222T* | *TaMFT-A1b* | *TaPHS1-646G* | *TaPHS1-666A* | 27.30% | 49.40% | 30.50% |
| 199 | Yanzhan 4110 | *TaSdr-A1a* | *TaSdr-B1a* | *Tamyb10-A1a* | *Tamyb10-B1a* | *Tamyb10-D1a* | *Vp-1Ba* | *TaDFR-Bb* | *TaMKK3-Ab* | *GS34-7Ba* | *TaMFT-3Aa* | *TaPHS1-222T* | *TaMFT-A1b* | *TaPHS1-646G* | *TaPHS1-666A* | 39.90% | 61.00% | 28.50% |
| 200 | Yimai 6 | *TaSdr-A1a* | *TaSdr-B1b* | *Tamyb10-A1a* | *Tamyb10-B1a* | *Tamyb10-D1a* | *Vp-1Ba* | *TaDFR-Bb* | *TaMKK3-Ab* | *GS34-7Ba* | *TaMFT-3Ab* | *TaPHS1-222T* | *TaMFT-A1a* | *TaPHS1-646G* | *TaPHS1-666T* | 86.20% | 87.20% | 80.70% |
| 201 | Yubao 1 | *TaSdr-A1a* | *TaSdr-B1b* | *Tamyb10-A1a* | *Tamyb10-B1a* | *Tamyb10-D1a* | *Vp-1Bc* | *TaDFR-Ba* | *TaMKK3-Aa* | *GS34-7Ba* | *TaMFT-3Ab* | *TaPHS1-222T* | *TaMFT-A1a* | *TaPHS1-646A* | *TaPHS1-666T* | 71.00% | 74.70% | 44.90% |
| 202 | Yujiao 5 | *TaSdr-A1a* | *TaSdr-B1b* | *Tamyb10-A1a* | *Tamyb10-B1a* | *Tamyb10-D1a* | *Vp-1Bc* | *TaDFR-Bb* | *TaMKK3-Ab* | *GS34-7Ba* | *TaMFT-3Aa* | *TaPHS1-222T* | *TaMFT-A1a* | *TaPHS1-646G* | *TaPHS1-666A* | 49.00% | 68.50% | 35.70% |
| 203 | Yumai 10 | *TaSdr-A1a* | *TaSdr-B1b* | *Tamyb10-A1a* | *Tamyb10-B1a* | *Tamyb10-D1a* | *Vp-1Ba* | *TaDFR-Ba* | *TaMKK3-Aa* | *GS34-7Ba* | *TaMFT-3Ab* | *TaPHS1-222T* | *TaMFT-A1a* | *TaPHS1-646A* | *TaPHS1-666T* | 56.80% | 71.90% | 32.40% |
| 204 | Yumai 13 | *TaSdr-A1b* | *TaSdr-B1b* | *Tamyb10-A1a* | *Tamyb10-B1a* | *Tamyb10-D1a* | *Vp-1Ba* | *TaDFR-Bb* | *TaMKK3-Ab* | *GS34-7Ba* | *TaMFT-3Aa* | *TaPHS1-222C* | *TaMFT-A1b* | *TaPHS1-646G* | *TaPHS1-666A* | 30.90% | 62.00% | 28.90% |
| 205 | Yumai 18 | *TaSdr-A1a* | *TaSdr-B1a* | *Tamyb10-A1b* | *Tamyb10-B1a* | *Tamyb10-D1a* | *Vp-1Ba* | *TaDFR-Bb* | *TaMKK3-Ab* | *GS34-7Ba* | *TaMFT-3Aa* | *TaPHS1-222T* | *TaMFT-A1b* | *TaPHS1-646G* | *TaPHS1-666A* | 29.00% | 51.80% | 33.90% |
| 206 | Yumai 2 | *TaSdr-A1a* | *TaSdr-B1b* | *Tamyb10-A1a* | *Tamyb10-B1a* | *Tamyb10-D1a* | *Vp-1Ba* | *TaDFR-Ba* | *TaMKK3-Aa* | *GS34-7Ba* | *TaMFT-3Aa* | *TaPHS1-222T* | *TaMFT-A1b* | *TaPHS1-646G* | *TaPHS1-666T* | 38.10% | 42.90% | 10.70% |
| 207 | Yumai 21 | *TaSdr-A1a* | *TaSdr-B1b* | *Tamyb10-A1a* | *Tamyb10-B1a* | *Tamyb10-D1a* | *Vp-1Bc* | *TaDFR-Bb* | *TaMKK3-Ab* | *GS34-7Ba* | *TaMFT-3Aa* | *TaPHS1-222T* | *TaMFT-A1b* | *TaPHS1-646G* | *TaPHS1-666A* | 57.00% | 77.50% | 35.00% |
| 208 | Yumai 29 | *TaSdr-A1a* | *TaSdr-B1b* | *Tamyb10-A1a* | *Tamyb10-B1a* | *Tamyb10-D1a* | *Vp-1Bc* | *TaDFR-Bb* | *TaMKK3-Aa* | *GS34-7Ba* | *TaMFT-3Aa* | *TaPHS1-222T* | *TaMFT-A1a* | *TaPHS1-646G* | *TaPHS1-666A* | 54.30% | 54.00% | 51.60% |
| 209 | Yumai 34 | *TaSdr-A1b* | *TaSdr-B1a* | *Tamyb10-A1a* | *Tamyb10-B1a* | *Tamyb10-D1a* | *Vp-1Bc* | *TaDFR-Bb* | *TaMKK3-Aa* | *GS34-7Ba* | *TaMFT-3Aa* | *TaPHS1-222T* | *TaMFT-A1b* | *TaPHS1-646G* | *TaPHS1-666A* | 47.50% | 61.10% | 42.10% |
| 210 | Yumai 38 | *TaSdr-A1b* | *TaSdr-B1a* | *Tamyb10-A1a* | *Tamyb10-B1a* | *Tamyb10-D1a* | *Vp-1Ba* | *TaDFR-Ba* | *TaMKK3-Aa* | *GS34-7Ba* | *TaMFT-3Aa* | *TaPHS1-222T* | *TaMFT-A1a* | *TaPHS1-646G* | *TaPHS1-666A* | 50.40% | 78.70% | 55.90% |
| 211 | Yumai 416 | *TaSdr-A1b* | *TaSdr-B1b* | *Tamyb10-A1a* | *Tamyb10-B1a* | *Tamyb10-D1a* | *Vp-1Bc* | *TaDFR-Ba* | *TaMKK3-Aa* | *GS34-7Ba* | *TaMFT-3Aa* | *TaPHS1-222T* | *TaMFT-A1b* | *TaPHS1-646G* | *TaPHS1-666A* | 54.90% | 69.20% | 49.50% |
| 212 | Yumai 47 | *TaSdr-A1a* | *TaSdr-B1b* | *Tamyb10-A1a* | *Tamyb10-B1a* | *Tamyb10-D1a* | *Vp-1Ba* | *TaDFR-Ba* | *TaMKK3-Ab* | *GS34-7Ba* | *TaMFT-3Ab* | *TaPHS1-222T* | *TaMFT-A1a* | *TaPHS1-646A* | *TaPHS1-666T* | 74.90% | 83.60% | 51.30% |
| 213 | Yumai 49 | *TaSdr-A1a* | *TaSdr-B1b* | *Tamyb10-A1a* | *Tamyb10-B1a* | *Tamyb10-D1a* | *Vp-1Bc* | *TaDFR-Ba* | *TaMKK3-Aa* | *GS34-7Ba* | *TaMFT-3Ab* | *TaPHS1-222T* | *TaMFT-A1a* | *TaPHS1-646G* | *TaPHS1-666T* | 35.20% | 45.70% | 21.00% |
| 214 | Yumai 49-198 | *TaSdr-A1a* | *TaSdr-B1b* | *Tamyb10-A1a* | *Tamyb10-B1a* | *Tamyb10-D1a* | *Vp-1Bc* | *TaDFR-Ba* | *TaMKK3-Ab* | *GS34-7Ba* | *TaMFT-3Ab* | *TaPHS1-222T* | *TaMFT-A1b* | *TaPHS1-646A* | *TaPHS1-666T* | 43.00% | 35.60% | 13.50% |
| 215 | Yumai 52 | *TaSdr-A1b* | *TaSdr-B1b* | *Tamyb10-A1a* | *Tamyb10-B1a* | *Tamyb10-D1a* | *Vp-1Ba* | *TaDFR-Bb* | *TaMKK3-Ab* | *GS34-7Ba* | *TaMFT-3Aa* | *TaPHS1-222T* | *TaMFT-A1a* | *TaPHS1-646G* | *TaPHS1-666T* | 43.70% | 49.90% | 12.80% |
| 216 | Yumai 54 | *TaSdr-A1b* | *TaSdr-B1b* | *Tamyb10-A1a* | *Tamyb10-B1a* | *Tamyb10-D1a* | *Vp-1Bc* | *TaDFR-Ba* | *TaMKK3-Aa* | *GS34-7Ba* | *TaMFT-3Aa* | *TaPHS1-222T* | *TaMFT-A1b* | *TaPHS1-646G* | *TaPHS1-666A* | 21.10% | 45.60% | 50.90% |
| 217 | Yumai 58 | *TaSdr-A1a* | *TaSdr-B1b* | *Tamyb10-A1a* | *Tamyb10-B1b* | *Tamyb10-D1a* | *Vp-1Bc* | *TaDFR-Ba* | *TaMKK3-Aa* | *GS34-7Ba* | *TaMFT-3Ab* | *TaPHS1-222T* | *TaMFT-A1a* | *TaPHS1-646G* | *TaPHS1-666T* | 41.00% | 61.10% | 12.20% |
| 218 | Yumai 7 | *TaSdr-A1a* | *TaSdr-B1a* | *Tamyb10-A1a* | *Tamyb10-B1a* | *Tamyb10-D1a* | *Vp-1Bc* | *TaDFR-Ba* | *TaMKK3-Ab* | *GS34-7Ba* | *TaMFT-3Aa* | *TaPHS1-222T* | *TaMFT-A1a* | *TaPHS1-646G* | *TaPHS1-666A* | 43.20% | 63.80% | 67.20% |
| 219 | Yumai 70 | *TaSdr-A1a* | *TaSdr-B1a* | *Tamyb10-A1a* | *Tamyb10-B1a* | *Tamyb10-D1a* | *Vp-1Bc* | *TaDFR-Bb* | *TaMKK3-Aa* | *GS34-7Ba* | *TaMFT-3Aa* | *TaPHS1-222T* | *TaMFT-A1b* | *TaPHS1-646G* | *TaPHS1-666A* | 43.60% | 62.10% | 23.90% |
| 220 | Yumai 70-36 | *TaSdr-A1a* | *TaSdr-B1b* | *Tamyb10-A1a* | *Tamyb10-B1a* | *Tamyb10-D1a* | *Vp-1Bc* | *TaDFR-Ba* | *TaMKK3-Ab* | *GS34-7Ba* | *TaMFT-3Aa* | *TaPHS1-222T* | *TaMFT-A1b* | *TaPHS1-646G* | *TaPHS1-666A* | 41.90% | 53.10% | 26.20% |
| 221 | Yunong 202 | *TaSdr-A1a* | *TaSdr-B1b* | *Tamyb10-A1a* | *Tamyb10-B1a* | *Tamyb10-D1a* | *Vp-1Bc* | *TaDFR-Ba* | *TaMKK3-Ab* | *GS34-7Ba* | *TaMFT-3Aa* | *TaPHS1-222T* | *TaMFT-A1b* | *TaPHS1-646G* | *TaPHS1-666A* | 35.00% | 46.50% | 15.00% |
| 222 | Yunong 949 | *TaSdr-A1b* | *TaSdr-B1b* | *Tamyb10-A1a* | *Tamyb10-B1a* | *Tamyb10-D1a* | *Vp-1Bc* | *TaDFR-Ba* | *TaMKK3-Aa* | *GS34-7Ba* | *TaMFT-3Aa* | *TaPHS1-222T* | *TaMFT-A1b* | *TaPHS1-646G* | *TaPHS1-666A* | 56.30% | 70.50% | 25.00% |
| 223 | Yunong 982 | *TaSdr-A1a* | *TaSdr-B1b* | *Tamyb10-A1a* | *Tamyb10-B1a* | *Tamyb10-D1a* | *Vp-1Bc* | *TaDFR-Ba* | *TaMKK3-Ab* | *GS34-7Ba* | *TaMFT-3Aa* | *TaPHS1-222T* | *TaMFT-A1b* | *TaPHS1-646G* | *TaPHS1-666A* | 25.10% | 33.20% | 15.00% |
| 224 | Zhengmai 004 | *TaSdr-A1a* | *TaSdr-B1b* | *Tamyb10-A1a* | *Tamyb10-B1a* | *Tamyb10-D1a* | *Vp-1Bc* | *TaDFR-Ba* | *TaMKK3-Ab* | *GS34-7Bb* | *TaMFT-3Aa* | *TaPHS1-222T* | *TaMFT-A1a* | *TaPHS1-646A* | *TaPHS1-666T* | 80.30% | 77.50% | 29.80% |
| 225 | Zhengmai 366 | *TaSdr-A1a* | *TaSdr-B1b* | *Tamyb10-A1a* | *Tamyb10-B1a* | *Tamyb10-D1a* | *Vp-1Bc* | *TaDFR-Bb* | *TaMKK3-Aa* | *GS34-7Ba* | *TaMFT-3Aa* | *TaPHS1-222T* | *TaMFT-A1a* | *TaPHS1-646G* | *TaPHS1-666A* | 15.30% | 48.10% | 31.40% |
| 226 | Zhengmai 379 | *TaSdr-A1a* | *TaSdr-B1b* | *Tamyb10-A1a* | *Tamyb10-B1a* | *Tamyb10-D1a* | *Vp-1Bc* | *TaDFR-Ba* | *TaMKK3-Ab* | *GS34-7Ba* | *TaMFT-3Aa* | *TaPHS1-222T* | *TaMFT-A1a* | *TaPHS1-646G* | *TaPHS1-666T* | 64.30% | 78.50% | 52.10% |
| 227 | Zhengmai 7698 | *TaSdr-A1a* | *TaSdr-B1b* | *Tamyb10-A1a* | *Tamyb10-B1a* | *Tamyb10-D1a* | *Vp-1Ba* | *TaDFR-Bb* | *TaMKK3-Aa* | *GS34-7Ba* | *TaMFT-3Aa* | *TaPHS1-222T* | *TaMFT-A1b* | *TaPHS1-646G* | *TaPHS1-666A* | 32.40% | 69.70% | 45.70% |
| 228 | Zhengmai 9023 | *TaSdr-A1a* | *TaSdr-B1a* | *Tamyb10-A1a* | *Tamyb10-B1a* | *Tamyb10-D1a* | *Vp-1Bc* | *TaDFR-Bb* | *TaMKK3-Ab* | *GS34-7Bb* | *TaMFT-3Aa* | *TaPHS1-222T* | *TaMFT-A1b* | *TaPHS1-646G* | *TaPHS1-666A* | 35.00% | 71.20% | 49.20% |
| 229 | Zhengmai 9962 | *TaSdr-A1a* | *TaSdr-B1a* | *Tamyb10-A1a* | *Tamyb10-B1a* | *Tamyb10-D1a* | *Vp-1Ba* | *TaDFR-Ba* | *TaMKK3-Ab* | *GS34-7Ba* | *TaMFT-3Aa* | *TaPHS1-222C* | *TaMFT-A1b* | *TaPHS1-646G* | *TaPHS1-666A* | 35.40% | 35.60% | 13.00% |
| 230 | Zhengnong 16 | *TaSdr-A1a* | *TaSdr-B1a* | *Tamyb10-A1a* | *Tamyb10-B1a* | *Tamyb10-D1a* | *Vp-1Bc* | *TaDFR-Bb* | *TaMKK3-Aa* | *GS34-7Ba* | *TaMFT-3Aa* | *TaPHS1-222T* | *TaMFT-A1b* | *TaPHS1-646G* | *TaPHS1-666A* | 77.00% | 74.60% | 45.70% |
| 231 | Zhengnong 17 | *TaSdr-A1a* | *TaSdr-B1b* | *Tamyb10-A1a* | *Tamyb10-B1a* | *Tamyb10-D1a* | *Vp-1Bc* | *TaDFR-Bb* | *TaMKK3-Ab* | *GS34-7Ba* | *TaMFT-3Aa* | *TaPHS1-222T* | *TaMFT-A1b* | *TaPHS1-646G* | *TaPHS1-666A* | 56.10% | 60.60% | 27.80% |
| 232 | Zhengyumai 9987 | *TaSdr-A1a* | *TaSdr-B1b* | *Tamyb10-A1a* | *Tamyb10-B1a* | *Tamyb10-D1a* | *Vp-1Bc* | *TaDFR-Ba* | *TaMKK3-Aa* | *GS34-7Ba* | *TaMFT-3Aa* | *TaPHS1-222T* | *TaMFT-A1a* | *TaPHS1-646G* | *TaPHS1-666A* | 65.80% | 83.90% | 64.70% |
| 233 | Zhengzhou 3 | *TaSdr-A1a* | *TaSdr-B1a* | *Tamyb10-A1a* | *Tamyb10-B1a* | *Tamyb10-D1b* | *Vp-1Bc* | *TaDFR-Bb* | *TaMKK3-Aa* | *GS34-7Ba* | *TaMFT-3Aa* | *TaPHS1-222T* | *TaMFT-A1a* | *TaPHS1-646G* | *TaPHS1-666A* | 90.20% | 89.40% | 54.90% |
| 234 | Zhengzhou 761 | *TaSdr-A1a* | *TaSdr-B1a* | *Tamyb10-A1a* | *Tamyb10-B1a* | *Tamyb10-D1a* | *Vp-1Bc* | *TaDFR-Ba* | *TaMKK3-Ab* | *GS34-7Ba* | *TaMFT-3Aa* | *TaPHS1-222T* | *TaMFT-A1b* | *TaPHS1-646G* | *TaPHS1-666A* | 11.70% | 55.00% | 21.30% |
| 235 | Zhongmai 1 | *TaSdr-A1a* | *TaSdr-B1b* | *Tamyb10-A1a* | *Tamyb10-B1a* | *Tamyb10-D1a* | *Vp-1Ba* | *TaDFR-Bb* | *TaMKK3-Aa* | *GS34-7Ba* | *TaMFT-3Aa* | *TaPHS1-222T* | *TaMFT-A1b* | *TaPHS1-646G* | *TaPHS1-666A* | 44.20% | 40.50% | 20.40% |
| 236 | Zhongmai 2 | *TaSdr-A1a* | *TaSdr-B1a* | *Tamyb10-A1a* | *Tamyb10-B1a* | *Tamyb10-D1a* | *Vp-1Bb* | *TaDFR-Bb* | *TaMKK3-Aa* | *GS34-7Ba* | *TaMFT-3Aa* | *TaPHS1-222T* | *TaMFT-A1b* | *TaPHS1-646G* | *TaPHS1-666A* | 30.40% | 41.70% | 11.00% |
| 237 | Zhongmai 875 | *TaSdr-A1a* | *TaSdr-B1b* | *Tamyb10-A1a* | *Tamyb10-B1a* | *Tamyb10-D1a* | *Vp-1Bc* | *TaDFR-Ba* | *TaMKK3-Aa* | *GS34-7Ba* | *TaMFT-3Ab* | *TaPHS1-222T* | *TaMFT-A1a* | *TaPHS1-646A* | *TaPHS1-666T* | 67.00% | 65.90% | 68.40% |
| 238 | Zhongmai 895 | *TaSdr-A1a* | *TaSdr-B1b* | *Tamyb10-A1a* | *Tamyb10-B1a* | *Tamyb10-D1a* | *Vp-1Ba* | *TaDFR-Bb* | *TaMKK3-Aa* | *GS34-7Ba* | *TaMFT-3Aa* | *TaPHS1-222T* | *TaMFT-A1b* | *TaPHS1-646G* | *TaPHS1-666A* | 48.60% | 79.30% | 49.30% |
| 239 | Zhongmai 998 | *TaSdr-A1a* | *TaSdr-B1b* | *Tamyb10-A1a* | *Tamyb10-B1a* | *Tamyb10-D1a* | *Vp-1Ba* | *TaDFR-Bb* | *TaMKK3-Aa* | *GS34-7Ba* | *TaMFT-3Aa* | *TaPHS1-222T* | *TaMFT-A1a* | *TaPHS1-646G* | *TaPHS1-666A* | 66.40% | 74.30% | 43.20% |
| 240 | Zhongyu 10 | *TaSdr-A1a* | *TaSdr-B1b* | *Tamyb10-A1a* | *Tamyb10-B1a* | *Tamyb10-D1a* | *Vp-1Ba* | *TaDFR-Ba* | *TaMKK3-Aa* | *GS34-7Ba* | *TaMFT-3Aa* | *TaPHS1-222T* | *TaMFT-A1a* | *TaPHS1-646A* | *TaPHS1-666T* | 74.50% | 74.10% | 45.30% |
| 241 | Zhongyu 12 | *TaSdr-A1a* | *TaSdr-B1b* | *Tamyb10-A1a* | *Tamyb10-B1a* | *Tamyb10-D1a* | *Vp-1Ba* | *TaDFR-Ba* | *TaMKK3-Aa* | *GS34-7Ba* | *TaMFT-3Aa* | *TaPHS1-222T* | *TaMFT-A1b* | *TaPHS1-646G* | *TaPHS1-666A* | 67.50% | 63.20% | 42.30% |
| 242 | Zhongyu 6 | *TaSdr-A1a* | *TaSdr-B1a* | *Tamyb10-A1a* | *Tamyb10-B1a* | *Tamyb10-D1a* | *Vp-1Bc* | *TaDFR-Bb* | *TaMKK3-Ab* | *GS34-7Ba* | *TaMFT-3Ab* | *TaPHS1-222T* | *TaMFT-A1a* | *TaPHS1-646G* | *TaPHS1-666A* | 60.40% | 63.20% | 67.20% |
| 243 | Zhongyu 9 | *TaSdr-A1a* | *TaSdr-B1b* | *Tamyb10-A1a* | *Tamyb10-B1a* | *Tamyb10-D1a* | *Vp-1Bb* | *TaDFR-Bb* | *TaMKK3-Aa* | *GS34-7Ba* | *TaMFT-3Ab* | *TaPHS1-222T* | *TaMFT-A1a* | *—* | *—* | 56.00% | 59.00% | 33.90% |
| 244 | Zhongyu 9398 | *TaSdr-A1a* | *TaSdr-B1a* | *Tamyb10-A1a* | *Tamyb10-B1a* | *Tamyb10-D1a* | *Vp-1Bc* | *TaDFR-Ba* | *TaMKK3-Ab* | *GS34-7Ba* | *TaMFT-3Aa* | *TaPHS1-222T* | *TaMFT-A1b* | *TaPHS1-646G* | *TaPHS1-666A* | 46.60% | 45.80% | 40.70% |
| 245 | Zhoumai 11 | *TaSdr-A1a* | *TaSdr-B1b* | *Tamyb10-A1a* | *Tamyb10-B1a* | *Tamyb10-D1a* | *Vp-1Ba* | *TaDFR-Bb* | *TaMKK3-Aa* | *GS34-7Ba* | *TaMFT-3Ab* | *TaPHS1-222T* | *TaMFT-A1a* | *TaPHS1-646G* | *TaPHS1-666A* | 38.70% | 71.00% | 34.10% |
| 246 | Zhoumai 16 | *TaSdr-A1a* | *TaSdr-B1b* | *Tamyb10-A1a* | *Tamyb10-B1a* | *Tamyb10-D1a* | *Vp-1Ba* | *TaDFR-Bb* | *TaMKK3-Aa* | *GS34-7Ba* | *TaMFT-3Aa* | *TaPHS1-222T* | *TaMFT-A1a* | *TaPHS1-646G* | *TaPHS1-666A* | 63.30% | 71.80% | 51.10% |
| 247 | Zhoumai 17 | *TaSdr-A1a* | *TaSdr-B1a* | *Tamyb10-A1a* | *Tamyb10-B1a* | *Tamyb10-D1a* | *Vp-1Ba* | *TaDFR-Bb* | *TaMKK3-Ab* | *GS34-7Ba* | *TaMFT-3Aa* | *TaPHS1-222T* | *TaMFT-A1b* | *TaPHS1-646G* | *TaPHS1-666A* | 69.30% | 75.90% | 42.30% |
| 248 | Zhoumai 18 | *TaSdr-A1b* | *TaSdr-B1b* | *Tamyb10-A1a* | *Tamyb10-B1a* | *Tamyb10-D1a* | *Vp-1Ba* | *TaDFR-Bb* | *TaMKK3-Ab* | *GS34-7Ba* | *TaMFT-3Aa* | *TaPHS1-222T* | *TaMFT-A1b* | *TaPHS1-646G* | *TaPHS1-666A* | 61.20% | 72.50% | 32.40% |
| 249 | Zhoumai 19 | *TaSdr-A1a* | *TaSdr-B1a* | *Tamyb10-A1a* | *Tamyb10-B1a* | *Tamyb10-D1a* | *Vp-1Bc* | *TaDFR-Bb* | *TaMKK3-Aa* | *GS34-7Ba* | *TaMFT-3Aa* | *TaPHS1-222T* | *TaMFT-A1b* | *TaPHS1-646G* | *TaPHS1-666A* | 47.00% | 66.80% | 25.00% |
| 250 | Zhoumai 20 | *TaSdr-A1a* | *TaSdr-B1a* | *Tamyb10-A1a* | *Tamyb10-B1a* | *Tamyb10-D1a* | *Vp-1Ba* | *TaDFR-Bb* | *TaMKK3-Aa* | *GS34-7Ba* | *TaMFT-3Aa* | *TaPHS1-222T* | *TaMFT-A1b* | *—* | *—* | 32.20% | 68.10% | 55.70% |
| 251 | Zhoumai 22 | *TaSdr-A1a* | *TaSdr-B1b* | *Tamyb10-A1a* | *Tamyb10-B1a* | *Tamyb10-D1a* | *Vp-1Ba* | *TaDFR-Bb* | *TaMKK3-Ab* | *GS34-7Ba* | *TaMFT-3Aa* | *TaPHS1-222T* | *TaMFT-A1b* | *TaPHS1-646G* | *TaPHS1-666T* | 55.40% | 64.10% | 36.30% |
| 252 | Zhoumai 23 | *TaSdr-A1a* | *TaSdr-B1a* | *Tamyb10-A1a* | *Tamyb10-B1a* | *Tamyb10-D1a* | *Vp-1Bc* | *TaDFR-Bb* | *TaMKK3-Aa* | *GS34-7Ba* | *TaMFT-3Ab* | *TaPHS1-222T* | *TaMFT-A1a* | *TaPHS1-646A* | *TaPHS1-666T* | 65.40% | 78.60% | 58.90% |
| 253 | Zhoumai 24 | *TaSdr-A1a* | *TaSdr-B1b* | *Tamyb10-A1a* | *Tamyb10-B1a* | *Tamyb10-D1a* | *Vp-1Ba* | *TaDFR-Ba* | *TaMKK3-Ab* | *GS34-7Ba* | *TaMFT-3Aa* | *TaPHS1-222T* | *TaMFT-A1b* | *TaPHS1-646G* | *TaPHS1-666T* | 35.20% | 59.40% | 11.50% |
| 254 | Zhoumai 25 | *TaSdr-A1a* | *TaSdr-B1b* | *Tamyb10-A1a* | *Tamyb10-B1a* | *Tamyb10-D1a* | *Vp-1Ba* | *TaDFR-Bb* | *TaMKK3-Ab* | *GS34-7Ba* | *TaMFT-3Aa* | *TaPHS1-222T* | *TaMFT-A1a* | *TaPHS1-646G* | *TaPHS1-666A* | 39.70% | 61.40% | 22.30% |
| 255 | Zhoumai 26 | *TaSdr-A1a* | *TaSdr-B1b* | *Tamyb10-A1a* | *Tamyb10-B1a* | *Tamyb10-D1a* | *Vp-1Ba* | *TaDFR-Bb* | *TaMKK3-Aa* | *GS34-7Ba* | *TaMFT-3Aa* | *TaPHS1-222T* | *TaMFT-A1a* | *TaPHS1-646G* | *TaPHS1-666A* | 71.50% | 49.40% | 51.70% |
| 256 | Zhoumai 27 | *TaSdr-A1a* | *TaSdr-B1b* | *Tamyb10-A1a* | *Tamyb10-B1a* | *Tamyb10-D1a* | *Vp-1Bc* | *TaDFR-Bb* | *TaMKK3-Aa* | *GS34-7Ba* | *TaMFT-3Aa* | *TaPHS1-222T* | *TaMFT-A1a* | *TaPHS1-646G* | *TaPHS1-666A* | 57.40% | 53.60% | 43.10% |
| 257 | Zhoumai 28 | *TaSdr-A1a* | *TaSdr-B1b* | *Tamyb10-A1a* | *Tamyb10-B1a* | *Tamyb10-D1a* | *Vp-1Ba* | *TaDFR-Bb* | *TaMKK3-Ab* | *GS34-7Ba* | *TaMFT-3Aa* | *TaPHS1-222T* | *TaMFT-A1b* | *TaPHS1-646G* | *TaPHS1-666A* | 57.50% | 73.90% | 28.40% |
| 258 | Zhoumai 30 | *TaSdr-A1a* | *TaSdr-B1b* | *Tamyb10-A1a* | *Tamyb10-B1a* | *Tamyb10-D1a* | *Vp-1Ba* | *TaDFR-Bb* | *TaMKK3-Aa* | *GS34-7Ba* | *TaMFT-3Aa* | *TaPHS1-222T* | *TaMFT-A1b* | *TaPHS1-646G* | *TaPHS1-666A* | 41.40% | 54.80% | 31.70% |
| 259 | Zhoumai 32 | *TaSdr-A1b* | *TaSdr-B1b* | *Tamyb10-A1a* | *Tamyb10-B1a* | *Tamyb10-D1a* | *Vp-1Ba* | *TaDFR-Bb* | *TaMKK3-Ab* | *GS34-7Ba* | *TaMFT-3Aa* | *TaPHS1-222T* | *TaMFT-A1b* | *TaPHS1-646G* | *TaPHS1-666A* | 59.60% | 65.20% | 52.10% |
| 260 | Zhumai 4 | *TaSdr-A1b* | *TaSdr-B1b* | *Tamyb10-A1a* | *Tamyb10-B1a* | *Tamyb10-D1a* | *Vp-1Bc* | *TaDFR-Ba* | *TaMKK3-Ab* | *GS34-7Ba* | *TaMFT-3Aa* | *TaPHS1-222T* | *TaMFT-A1a* | *TaPHS1-646G* | *TaPHS1-666A* | 66.40% | 55.80% | 30.10% |
| 261 | Aifeng 3 | *TaSdr-A1a* | *TaSdr-B1b* | *Tamyb10-A1a* | *Tamyb10-B1a* | *Tamyb10-D1a* | *Vp-1Bc* | *TaDFR-Ba* | *TaMKK3-Aa* | *GS34-7Ba* | *TaMFT-3Aa* | *TaPHS1-222T* | *TaMFT-A1a* | *TaPHS1-646A* | *TaPHS1-666T* | 71.10% | 77.30% | 19.00% |
| 262 | Fengchan 3 | *TaSdr-A1a* | *TaSdr-B1a* | *Tamyb10-A1a* | *Tamyb10-B1a* | *Tamyb10-D1a* | *Vp-1Bc* | *TaDFR-Ba* | *TaMKK3-Aa* | *GS34-7Ba* | *TaMFT-3Aa* | *TaPHS1-222C* | *TaMFT-A1a* | *TaPHS1-646G* | *TaPHS1-666A* | 5.30% | 39.70% | 4.10% |
| 263 | Jinfeng 3 | *TaSdr-A1b* | *TaSdr-B1b* | *Tamyb10-A1a* | *Tamyb10-B1a* | *Tamyb10-D1a* | *Vp-1Bc* | *TaDFR-Ba* | *TaMKK3-Aa* | *GS34-7Ba* | *TaMFT-3Ab* | *TaPHS1-222T* | *TaMFT-A1a* | *TaPHS1-646G* | *TaPHS1-666A* | 55.20% | 37.20% | 14.10% |
| 264 | Shan 229 | *TaSdr-A1a* | *TaSdr-B1a* | *Tamyb10-A1a* | *Tamyb10-B1a* | *Tamyb10-D1a* | *Vp-1Bc* | *TaDFR-Bb* | *TaMKK3-Ab* | *GS34-7Ba* | *TaMFT-3Aa* | *TaPHS1-222T* | *TaMFT-A1b* | *TaPHS1-646G* | *TaPHS1-666A* | 26.70% | 52.80% | 12.60% |
| 265 | Xiaan 8 | *TaSdr-A1a* | *TaSdr-B1b* | *Tamyb10-A1a* | *Tamyb10-B1a* | *Tamyb10-D1a* | *Vp-1Bc* | *TaDFR-Bb* | *TaMKK3-Aa* | *GS34-7Bb* | *TaMFT-3Aa* | *TaPHS1-222T* | *TaMFT-A1b* | *—* | *—* | 40.80% | 47.00% | 37.20% |
| 266 | Xiaoyan 22 | *TaSdr-A1a* | *TaSdr-B1b* | *Tamyb10-A1a* | *Tamyb10-B1a* | *Tamyb10-D1a* | *Vp-1Ba* | *TaDFR-Bb* | *TaMKK3-Ab* | *GS34-7Ba* | *TaMFT-3Aa* | *TaPHS1-222T* | *TaMFT-A1a* | *TaPHS1-646A* | *TaPHS1-666T* | 74.00% | 84.70% | 50.40% |
| 267 | Xiaoyan 54 | *TaSdr-A1a* | *TaSdr-B1b* | *Tamyb10-A1a* | *Tamyb10-B1a* | *Tamyb10-D1a* | *Vp-1Bc* | *TaDFR-Ba* | *TaMKK3-Ab* | *GS34-7Ba* | *TaMFT-3Aa* | *TaPHS1-222T* | *TaMFT-A1b* | *TaPHS1-646G* | *TaPHS1-666A* | 33.40% | 40.30% | 18.20% |
| 268 | XiaoYan 6 | *TaSdr-A1a* | *TaSdr-B1a* | *Tamyb10-A1a* | *Tamyb10-B1a* | *Tamyb10-D1a* | *Vp-1Bc* | *TaDFR-Bb* | *TaMKK3-Ab* | *GS34-7Ba* | *TaMFT-3Aa* | *TaPHS1-222T* | *TaMFT-A1b* | *TaPHS1-646G* | *TaPHS1-666A* | 14.70% | 50.80% | 21.90% |
| 269 | Xinong 219 | *TaSdr-A1a* | *TaSdr-B1b* | *Tamyb10-A1a* | *Tamyb10-B1a* | *Tamyb10-D1a* | *Vp-1Bc* | *TaDFR-Ba* | *TaMKK3-Ab* | *GS34-7Ba* | *TaMFT-3Aa* | *TaPHS1-222T* | *TaMFT-A1a* | *TaPHS1-646G* | *TaPHS1-666A* | 51.20% | 63.60% | 50.80% |
| 270 | Xinong 529 | *TaSdr-A1b* | *TaSdr-B1a* | *Tamyb10-A1a* | *Tamyb10-B1a* | *Tamyb10-D1a* | *Vp-1Bc* | *TaDFR-Ba* | *TaMKK3-Ab* | *GS34-7Ba* | *TaMFT-3Aa* | *TaPHS1-222T* | *TaMFT-A1a* | *TaPHS1-646G* | *TaPHS1-666A* | 39.70% | 56.60% | 22.70% |
| 271 | Xinong 979 | *TaSdr-A1a* | *TaSdr-B1a* | *Tamyb10-A1a* | *Tamyb10-B1a* | *Tamyb10-D1a* | *Vp-1Bc* | *TaDFR-Bb* | *TaMKK3-Ab* | *GS34-7Ba* | *TaMFT-3Ab* | *TaPHS1-222T* | *TaMFT-A1a* | *TaPHS1-646G* | *TaPHS1-666A* | 25.50% | 65.00% | 20.30% |
| 272 | Wanmai 19 | *TaSdr-A1a* | *TaSdr-B1b* | *Tamyb10-A1a* | *Tamyb10-B1a* | *Tamyb10-D1a* | *Vp-1Bc* | *TaDFR-Bb* | *TaMKK3-Aa* | *GS34-7Ba* | *TaMFT-3Aa* | *TaPHS1-222T* | *TaMFT-A1b* | *TaPHS1-646G* | *TaPHS1-666A* | 57.00% | 61.70% | 40.50% |
| 273 | Wanmai 38 | *TaSdr-A1a* | *TaSdr-B1b* | *Tamyb10-A1a* | *Tamyb10-B1a* | *Tamyb10-D1a* | *Vp-1Bc* | *TaDFR-Bb* | *TaMKK3-Aa* | *GS34-7Ba* | *TaMFT-3Aa* | *TaPHS1-222T* | *TaMFT-A1b* | *TaPHS1-646A* | *TaPHS1-666T* | 14.10% | 58.90% | 52.20% |
| 274 | Wanmai 50 | *TaSdr-A1a* | *TaSdr-B1b* | *Tamyb10-A1a* | *Tamyb10-B1a* | *Tamyb10-D1a* | *Vp-1Bc* | *TaDFR-Bb* | *TaMKK3-Aa* | *GS34-7Ba* | *TaMFT-3Aa* | *TaPHS1-222T* | *TaMFT-A1a* | *TaPHS1-646A* | *TaPHS1-666T* | 72.00% | 69.00% | 15.90% |
| 275 | Wanmai 52 | *TaSdr-A1a* | *TaSdr-B1b* | *Tamyb10-A1a* | *Tamyb10-B1a* | *Tamyb10-D1a* | *Vp-1Bc* | *TaDFR-Ba* | *TaMKK3-Aa* | *GS34-7Ba* | *TaMFT-3Ab* | *TaPHS1-222T* | *TaMFT-A1a* | *TaPHS1-646G* | *TaPHS1-666A* | 16.60% | 37.30% | 9.90% |
| 276 | Wanmai 53 | *TaSdr-A1a* | *TaSdr-B1a* | *Tamyb10-A1a* | *Tamyb10-B1a* | *Tamyb10-D1a* | *Vp-1Bc* | *TaDFR-Bb* | *TaMKK3-Aa* | *GS34-7Ba* | *TaMFT-3Ab* | *TaPHS1-222T* | *TaMFT-A1a* | *TaPHS1-646A* | *TaPHS1-666T* | 52.00% | 55.50% | 14.30% |
| 277 | Baomai 10 | *TaSdr-A1a* | *TaSdr-B1a* | *Tamyb10-A1a* | *Tamyb10-B1a* | *Tamyb10-D1a* | *Vp-1Bc* | *TaDFR-Ba* | *TaMKK3-Aa* | *GS34-7Ba* | *TaMFT-3Aa* | *TaPHS1-222T* | *TaMFT-A1a* | *TaPHS1-646G* | *TaPHS1-666A* | 41.10% | 43.80% | 27.40% |
| 278 | Baomai 2 | *TaSdr-A1a* | *TaSdr-B1a* | *Tamyb10-A1a* | *Tamyb10-B1a* | *Tamyb10-D1a* | *Vp-1Bc* | *TaDFR-Ba* | *TaMKK3-Ab* | *GS34-7Ba* | *TaMFT-3Aa* | *TaPHS1-222T* | *TaMFT-A1b* | *TaPHS1-646G* | *TaPHS1-666A* | 35.70% | 34.30% | 18.10% |
| 279 | Huaimai 18 | *TaSdr-A1a* | *TaSdr-B1b* | *Tamyb10-A1b* | *Tamyb10-B1a* | *Tamyb10-D1b* | *Vp-1Ba* | *TaDFR-Bb* | *TaMKK3-Ab* | *GS34-7Ba* | *TaMFT-3Ab* | *TaPHS1-222T* | *TaMFT-A1a* | *TaPHS1-646A* | *TaPHS1-666T* | 87.90% | 85.50% | 75.80% |
| 280 | Huaimai 20 | *TaSdr-A1a* | *TaSdr-B1b* | *Tamyb10-A1a* | *Tamyb10-B1a* | *Tamyb10-D1a* | *Vp-1Bc* | *TaDFR-Bb* | *TaMKK3-Aa* | *GS34-7Ba* | *TaMFT-3Aa* | *TaPHS1-222T* | *TaMFT-A1a* | *TaPHS1-646G* | *TaPHS1-666A* | 24.00% | 61.00% | 32.60% |
| 281 | Huaimai 21 | *TaSdr-A1a* | *TaSdr-B1a* | *Tamyb10-A1a* | *Tamyb10-B1a* | *Tamyb10-D1a* | *Vp-1Bc* | *TaDFR-Ba* | *TaMKK3-Aa* | *GS34-7Ba* | *TaMFT-3Ab* | *TaPHS1-222T* | *TaMFT-A1a* | *TaPHS1-646A* | *TaPHS1-666T* | 48.20% | 48.10% | 26.60% |
| 282 | Huaimai 22 | *TaSdr-A1a* | *TaSdr-B1b* | *Tamyb10-A1a* | *Tamyb10-B1a* | *Tamyb10-D1a* | *Vp-1Bc* | *TaDFR-Bb* | *TaMKK3-Aa* | *GS34-7Ba* | *TaMFT-3Aa* | *TaPHS1-222T* | *TaMFT-A1a* | *TaPHS1-646G* | *TaPHS1-666A* | 27.60% | 35.50% | 17.40% |
| 283 | Huaimai 28 | *TaSdr-A1a* | *TaSdr-B1b* | *Tamyb10-A1a* | *Tamyb10-B1a* | *Tamyb10-D1b* | *Vp-1Ba* | *TaDFR-Bb* | *TaMKK3-Aa* | *GS34-7Ba* | *TaMFT-3Aa* | *TaPHS1-222T* | *TaMFT-A1a* | *TaPHS1-646G* | *TaPHS1-666A* | 54.40% | 54.80% | 34.00% |
| 284 | Huaimai 29 | *TaSdr-A1a* | *TaSdr-B1a* | *Tamyb10-A1a* | *Tamyb10-B1a* | *Tamyb10-D1a* | *Vp-1Bc* | *TaDFR-Bb* | *TaMKK3-Aa* | *GS34-7Ba* | *TaMFT-3Aa* | *TaPHS1-222T* | *TaMFT-A1a* | *TaPHS1-646G* | *TaPHS1-666A* | 39.80% | 58.40% | 32.70% |
| 285 | Lianmai 2 | *TaSdr-A1a* | *TaSdr-B1b* | *Tamyb10-A1a* | *Tamyb10-B1a* | *Tamyb10-D1a* | *Vp-1Bc* | *TaDFR-Bb* | *TaMKK3-Aa* | *GS34-7Ba* | *TaMFT-3Aa* | *TaPHS1-222T* | *TaMFT-A1a* | *TaPHS1-646G* | *TaPHS1-666A* | 21.20% | 53.70% | 21.90% |
| 286 | Xumai 30 | *TaSdr-A1a* | *TaSdr-B1b* | *Tamyb10-A1a* | *Tamyb10-B1a* | *Tamyb10-D1a* | *Vp-1Bc* | *TaDFR-Bb* | *TaMKK3-Aa* | *GS34-7Ba* | *TaMFT-3Aa* | *TaPHS1-222T* | *TaMFT-A1b* | *TaPHS1-646G* | *TaPHS1-666A* | 44.40% | 52.90% | 21.40% |
| 287 | Xumai 856 | *TaSdr-A1a* | *TaSdr-B1a* | *Tamyb10-A1a* | *Tamyb10-B1a* | *Tamyb10-D1a* | *Vp-1Ba* | *TaDFR-Bb* | *TaMKK3-Aa* | *GS34-7Ba* | *TaMFT-3Aa* | *TaPHS1-222T* | *TaMFT-A1a* | *TaPHS1-646G* | *TaPHS1-666A* | 49.90% | 56.10% | 8.00% |
| 288 | Xuzhou 24 | *TaSdr-A1a* | *TaSdr-B1b* | *Tamyb10-A1a* | *Tamyb10-B1a* | *Tamyb10-D1a* | *Vp-1Ba* | *TaDFR-Bb* | *TaMKK3-Aa* | *GS34-7Ba* | *TaMFT-3Aa* | *TaPHS1-222T* | *TaMFT-A1b* | *TaPHS1-646G* | *TaPHS1-666A* | 46.00% | 88.00% | 50.70% |
| 289 | Xuzhou 25 | *TaSdr-A1a* | *TaSdr-B1a* | *Tamyb10-A1a* | *Tamyb10-B1a* | *Tamyb10-D1a* | *Vp-1Ba* | *TaDFR-Ba* | *TaMKK3-Aa* | *GS34-7Bb* | *TaMFT-3Aa* | *TaPHS1-222T* | *TaMFT-A1b* | *TaPHS1-646G* | *TaPHS1-666A* | 52.20% | 79.10% | 62.00% |
| 290 | Huacheng 3366 | *TaSdr-A1a* | *TaSdr-B1b* | *Tamyb10-A1a* | *Tamyb10-B1a* | *Tamyb10-D1a* | *Vp-1Bc* | *TaDFR-Bb* | *TaMKK3-Aa* | *GS34-7Ba* | *TaMFT-3Aa* | *TaPHS1-222T* | *TaMFT-A1a* | *TaPHS1-646G* | *TaPHS1-666A* | 51.40% | 57.10% | 40.00% |
| 291 | Huamai 2566 | *TaSdr-A1b* | *TaSdr-B1a* | *Tamyb10-A1b* | *Tamyb10-B1a* | *Tamyb10-D1a* | *Vp-1Bc* | *TaDFR-Bb* | *TaMKK3-Ab* | *GS34-7Ba* | *TaMFT-3Ab* | *TaPHS1-222T* | *TaMFT-A1a* | *TaPHS1-646A* | *TaPHS1-666T* | 21.00% | 34.00% | 18.60% |
| 292 | Lunxuan 146 | *TaSdr-A1a* | *TaSdr-B1b* | *Tamyb10-A1a* | *Tamyb10-B1a* | *Tamyb10-D1a* | *Vp-1Ba* | *TaDFR-Bb* | *TaMKK3-Ab* | *GS34-7Ba* | *TaMFT-3Ab* | *TaPHS1-222T* | *TaMFT-A1a* | *TaPHS1-646G* | *TaPHS1-666A* | 68.30% | 82.40% | 33.10% |
| 293 | Xianmai 8 | *TaSdr-A1a* | *TaSdr-B1b* | *Tamyb10-A1a* | *Tamyb10-B1a* | *Tamyb10-D1a* | *Vp-1Bc* | *TaDFR-Ba* | *TaMKK3-Ab* | *GS34-7Ba* | *TaMFT-3Aa* | *TaPHS1-222T* | *TaMFT-A1b* | *TaPHS1-646G* | *TaPHS1-666A* | 53.40% | 75.10% | 36.10% |
| 294 | Nannong 0686 | *TaSdr-A1a* | *TaSdr-B1a* | *Tamyb10-A1b* | *Tamyb10-B1a* | *Tamyb10-D1a* | *Vp-1Bc* | *TaDFR-Ba* | *TaMKK3-Ab* | *GS34-7Bb* | *TaMFT-3Ab* | *TaPHS1-222T* | *TaMFT-A1a* | *TaPHS1-646A* | *TaPHS1-666T* | 12.40% | 29.00% | 10.60% |
| 295 | Ningmai 13 | *TaSdr-A1a* | *TaSdr-B1b* | *Tamyb10-A1a* | *Tamyb10-B1b* | *Tamyb10-D1a* | *Vp-1Ba* | *TaDFR-Bb* | *TaMKK3-Ab* | *GS34-7Bb* | *TaMFT-3Aa* | *TaPHS1-222T* | *TaMFT-A1a* | *TaPHS1-646G* | *TaPHS1-666A* | 35.00% | 39.20% | 19.00% |
| 296 | Ningmai 16 | *TaSdr-A1a* | *TaSdr-B1a* | *Tamyb10-A1a* | *Tamyb10-B1a* | *Tamyb10-D1b* | *Vp-1Ba* | *TaDFR-Ba* | *TaMKK3-Ab* | *GS34-7Bb* | *TaMFT-3Ab* | *TaPHS1-222T* | *TaMFT-A1a* | *TaPHS1-646A* | *TaPHS1-666T* | 17.00% | 35.90% | 16.80% |
| 297 | Ningmai 9 | *TaSdr-A1a* | *TaSdr-B1b* | *Tamyb10-A1a* | *Tamyb10-B1a* | *Tamyb10-D1b* | *Vp-1Ba* | *TaDFR-Ba* | *TaMKK3-Ab* | *GS34-7Bb* | *TaMFT-3Ab* | *TaPHS1-222T* | *TaMFT-A1a* | *TaPHS1-646G* | *TaPHS1-666T* | 31.00% | 38.10% | 14.60% |
| 298 | Shengxuan 6 | *TaSdr-A1a* | *TaSdr-B1a* | *Tamyb10-A1a* | *Tamyb10-B1a* | *Tamyb10-D1b* | *Vp-1Ba* | *TaDFR-Ba* | *TaMKK3-Ab* | *GS34-7Bb* | *TaMFT-3Ab* | *TaPHS1-222T* | *TaMFT-A1a* | *TaPHS1-646A* | *TaPHS1-666T* | 32.10% | 44.60% | 17.60% |
| 299 | Yangfumai 4 | *TaSdr-A1a* | *TaSdr-B1b* | *Tamyb10-A1b* | *Tamyb10-B1a* | *Tamyb10-D1b* | *Vp-1Ba* | *TaDFR-Ba* | *TaMKK3-Ab* | *GS34-7Bb* | *TaMFT-3Aa* | *TaPHS1-222T* | *TaMFT-A1b* | *TaPHS1-646G* | *TaPHS1-666A* | 12.00% | 24.90% | 9.80% |
| 300 | Yangmai 12 | *TaSdr-A1a* | *TaSdr-B1a* | *Tamyb10-A1b* | *Tamyb10-B1a* | *Tamyb10-D1b* | *Vp-1Bc* | *TaDFR-Ba* | *TaMKK3-Aa* | *GS34-7Bb* | *TaMFT-3Aa* | *TaPHS1-222T* | *TaMFT-A1b* | *TaPHS1-646G* | *TaPHS1-666A* | 23.90% | 17.90% | 12.30% |
| 301 | Yangmai 13 | *TaSdr-A1a* | *TaSdr-B1b* | *Tamyb10-A1a* | *Tamyb10-B1b* | *Tamyb10-D1a* | *Vp-1Bc* | *TaDFR-Bb* | *TaMKK3-Aa* | *GS34-7Ba* | *TaMFT-3Ab* | *TaPHS1-222T* | *TaMFT-A1b* | *TaPHS1-646G* | *TaPHS1-666A* | 44.80% | 46.90% | 23.00% |
| 302 | Yangmai 15 | *TaSdr-A1a* | *TaSdr-B1a* | *Tamyb10-A1a* | *Tamyb10-B1a* | *Tamyb10-D1b* | *Vp-1Bc* | *TaDFR-Bb* | *TaMKK3-Aa* | *GS34-7Ba* | *TaMFT-3Aa* | *TaPHS1-222T* | *TaMFT-A1b* | *TaPHS1-646G* | *TaPHS1-666A* | 22.80% | 17.70% | 3.10% |
| 303 | Yangmai 158 | *TaSdr-A1a* | *TaSdr-B1a* | *Tamyb10-A1a* | *Tamyb10-B1a* | *Tamyb10-D1b* | *Vp-1Bc* | *TaDFR-Ba* | *TaMKK3-Aa* | *GS34-7Bb* | *TaMFT-3Ab* | *TaPHS1-222T* | *TaMFT-A1a* | *TaPHS1-646G* | *TaPHS1-666A* | 31.20% | 23.70% | 11.60% |
| 304 | Yangmai 16 | *TaSdr-A1a* | *TaSdr-B1a* | *Tamyb10-A1b* | *Tamyb10-B1b* | *Tamyb10-D1b* | *Vp-1Bc* | *TaDFR-Ba* | *TaMKK3-Aa* | *GS34-7Bb* | *TaMFT-3Aa* | *TaPHS1-222T* | *TaMFT-A1a* | *TaPHS1-646A* | *TaPHS1-666T* | 12.80% | 25.20% | 9.00% |
| 305 | Yangmai 20 | *TaSdr-A1b* | *TaSdr-B1a* | *Tamyb10-A1b* | *Tamyb10-B1a* | *Tamyb10-D1b* | *Vp-1Bc* | *TaDFR-Bb* | *TaMKK3-Ab* | *GS34-7Ba* | *TaMFT-3Aa* | *TaPHS1-222T* | *TaMFT-A1b* | *TaPHS1-646G* | *TaPHS1-666A* | 11.00% | 17.10% | 6.60% |
| 306 | Yangmai 21 | *TaSdr-A1a* | *TaSdr-B1a* | *Tamyb10-A1b* | *Tamyb10-B1a* | *Tamyb10-D1b* | *Vp-1Bc* | *TaDFR-Ba* | *TaMKK3-Ab* | *GS34-7Bb* | *TaMFT-3Aa* | *TaPHS1-222T* | *TaMFT-A1a* | *TaPHS1-646A* | *TaPHS1-666T* | 27.70% | 40.70% | 7.90% |
| 307 | Yangmai 22 | *TaSdr-A1a* | *TaSdr-B1a* | *Tamyb10-A1b* | *Tamyb10-B1b* | *Tamyb10-D1b* | *Vp-1Bc* | *TaDFR-Bb* | *TaMKK3-Ab* | *GS34-7Ba* | *TaMFT-3Ab* | *TaPHS1-222T* | *TaMFT-A1b* | *TaPHS1-646G* | *TaPHS1-666A* | 11.50% | 31.00% | 13.90% |
| 308 | Yangmai 23 | *TaSdr-A1a* | *TaSdr-B1a* | *Tamyb10-A1a* | *Tamyb10-B1b* | *Tamyb10-D1b* | *Vp-1Bc* | *TaDFR-Ba* | *TaMKK3-Ab* | *GS34-7Bb* | *TaMFT-3Ab* | *TaPHS1-222T* | *TaMFT-A1b* | *TaPHS1-646A* | *TaPHS1-666T* | 9.30% | 38.10% | 8.40% |
| 309 | Zhenmai 168 | *TaSdr-A1a* | *TaSdr-B1a* | *Tamyb10-A1a* | *Tamyb10-B1b* | *Tamyb10-D1b* | *Vp-1Bc* | *TaDFR-Ba* | *TaMKK3-Ab* | *GS34-7Ba* | *TaMFT-3Ab* | *TaPHS1-222T* | *TaMFT-A1b* | *TaPHS1-646G* | *TaPHS1-666A* | 26.70% | 34.40% | 14.60% |
| 310 | Zhenmai 9 | *TaSdr-A1a* | *TaSdr-B1a* | *Tamyb10-A1b* | *Tamyb10-B1a* | *Tamyb10-D1b* | *Vp-1Ba* | *TaDFR-Ba* | *TaMKK3-Ab* | *GS34-7Bb* | *TaMFT-3Aa* | *TaPHS1-222T* | *TaMFT-A1b* | *TaPHS1-646G* | *TaPHS1-666A* | 24.60% | 38.50% | 30.90% |
| 311 | Chuanmai 22 | *TaSdr-A1a* | *TaSdr-B1a* | *Tamyb10-A1a* | *Tamyb10-B1a* | *Tamyb10-D1b* | *Vp-1Bc* | *TaDFR-Ba* | *TaMKK3-Aa* | *GS34-7Ba* | *TaMFT-3Aa* | *TaPHS1-222T* | *TaMFT-A1b* | *TaPHS1-646G* | *TaPHS1-666A* | 53.90% | 74.80% | 52.80% |
| 312 | Chuanmai 24 | *TaSdr-A1a* | *TaSdr-B1b* | *Tamyb10-A1b* | *Tamyb10-B1b* | *Tamyb10-D1a* | *Vp-1Bc* | *TaDFR-Ba* | *TaMKK3-Aa* | *GS34-7Ba* | *TaMFT-3Aa* | *TaPHS1-222T* | *TaMFT-A1b* | *TaPHS1-646G* | *TaPHS1-666A* | 83.10% | 74.50% | 30.30% |
| 313 | Chuanmai 28 | *TaSdr-A1a* | *TaSdr-B1a* | *Tamyb10-A1a* | *Tamyb10-B1a* | *Tamyb10-D1b* | *Vp-1Bc* | *TaDFR-Ba* | *TaMKK3-Aa* | *GS34-7Ba* | *TaMFT-3Aa* | *TaPHS1-222T* | *TaMFT-A1b* | *TaPHS1-646A* | *TaPHS1-666T* | 26.50% | 28.80% | 12.70% |
| 314 | Chuanmai 42 | *TaSdr-A1a* | *TaSdr-B1a* | *Tamyb10-A1b* | *Tamyb10-B1a* | *Tamyb10-D1a* | *Vp-1Bc* | *TaDFR-Ba* | *TaMKK3-Aa* | *GS34-7Ba* | *TaMFT-3Aa* | *TaPHS1-222T* | *TaMFT-A1b* | *TaPHS1-646G* | *TaPHS1-666A* | 30.80% | 42.90% | 28.60% |
| 315 | Chuanmai 46 | *TaSdr-A1a* | *TaSdr-B1a* | *Tamyb10-A1a* | *Tamyb10-B1a* | *Tamyb10-D1a* | *Vp-1Bc* | *TaDFR-Ba* | *TaMKK3-Ab* | *GS34-7Ba* | *TaMFT-3Ab* | *TaPHS1-222T* | *TaMFT-A1a* | *TaPHS1-646A* | *TaPHS1-666T* | 56.70% | 62.30% | 22.60% |
| 316 | Chuanmai 47 | *TaSdr-A1a* | *TaSdr-B1b* | *Tamyb10-A1a* | *Tamyb10-B1a* | *Tamyb10-D1a* | *Vp-1Ba* | *TaDFR-Bb* | *TaMKK3-Aa* | *GS34-7Ba* | *TaMFT-3Aa* | *TaPHS1-222T* | *TaMFT-A1a* | *TaPHS1-646G* | *TaPHS1-666A* | 65.10% | 72.00% | 31.00% |
| 317 | Chuanmai 64 | *TaSdr-A1b* | *TaSdr-B1b* | *Tamyb10-A1a* | *Tamyb10-B1a* | *Tamyb10-D1b* | *Vp-1Bc* | *TaDFR-Ba* | *—* | *GS34-7Ba* | *TaMFT-3Aa* | *TaPHS1-222T* | *TaMFT-A1b* | *TaPHS1-646G* | *TaPHS1-666A* | 31.50% | 36.20% | 10.80% |
| 318 | Chuanmai 107 | *TaSdr-A1a* | *TaSdr-B1a* | *Tamyb10-A1a* | *Tamyb10-B1b* | *Tamyb10-D1b* | *Vp-1Bc* | *TaDFR-Ba* | *TaMKK3-Aa* | *GS34-7Ba* | *TaMFT-3Aa* | *TaPHS1-222T* | *TaMFT-A1b* | *TaPHS1-646G* | *TaPHS1-666A* | 79.00% | 79.00% | 45.20% |
| 319 | Chuannong 16 | *TaSdr-A1a* | *TaSdr-B1b* | *Tamyb10-A1a* | *Tamyb10-B1b* | *Tamyb10-D1b* | *Vp-1Bc* | *TaDFR-Bb* | *TaMKK3-Ab* | *GS34-7Ba* | *TaMFT-3Aa* | *TaPHS1-222T* | *TaMFT-A1b* | *TaPHS1-646G* | *TaPHS1-666A* | 51.60% | 44.00% | 14.30% |
| 320 | Miannong 4 | *TaSdr-A1a* | *TaSdr-B1a* | *Tamyb10-A1a* | *Tamyb10-B1a* | *Tamyb10-D1b* | *Vp-1Ba* | *TaDFR-Ba* | *TaMKK3-Aa* | *GS34-7Ba* | *TaMFT-3Aa* | *TaPHS1-222T* | *TaMFT-A1b* | *TaPHS1-646G* | *TaPHS1-666A* | 76.70% | 75.00% | 46.40% |
| 321 | Mianyang 11 | *TaSdr-A1a* | *TaSdr-B1a* | *Tamyb10-A1a* | *Tamyb10-B1a* | *Tamyb10-D1b* | *Vp-1Ba* | *TaDFR-Ba* | *TaMKK3-Aa* | *GS34-7Ba* | *TaMFT-3Aa* | *TaPHS1-222T* | *TaMFT-A1b* | *TaPHS1-646G* | *TaPHS1-666A* | 49.30% | 51.00% | 25.00% |
| 322 | Mianyang 19 | *TaSdr-A1a* | *TaSdr-B1a* | *Tamyb10-A1b* | *Tamyb10-B1a* | *Tamyb10-D1a* | *Vp-1Bc* | *TaDFR-Ba* | *TaMKK3-Aa* | *GS34-7Ba* | *TaMFT-3Aa* | *TaPHS1-222T* | *TaMFT-A1a* | *TaPHS1-646G* | *TaPHS1-666T* | 60.30% | 55.70% | 18.70% |
| 323 | Mianyang 20 | *TaSdr-A1a* | *TaSdr-B1b* | *Tamyb10-A1a* | *Tamyb10-B1b* | *Tamyb10-D1a* | *Vp-1Bc* | *TaDFR-Ba* | *TaMKK3-Aa* | *GS34-7Ba* | *TaMFT-3Aa* | *TaPHS1-222T* | *TaMFT-A1b* | *TaPHS1-646G* | *TaPHS1-666A* | 75.50% | 79.10% | 34.00% |
| 324 | Mianyang 26 | *TaSdr-A1a* | *TaSdr-B1a* | *Tamyb10-A1a* | *Tamyb10-B1a* | *Tamyb10-D1a* | *Vp-1Bc* | *TaDFR-Ba* | *TaMKK3-Ab* | *GS34-7Ba* | *TaMFT-3Aa* | *TaPHS1-222T* | *TaMFT-A1b* | *TaPHS1-646G* | *TaPHS1-666A* | 66.30% | 64.10% | 22.80% |
| 325 | Neimai 8 | *TaSdr-A1a* | *TaSdr-B1a* | *Tamyb10-A1a* | *Tamyb10-B1a* | *Tamyb10-D1a* | *Vp-1Ba* | *TaDFR-Bb* | *TaMKK3-Ab* | *GS34-7Ba* | *TaMFT-3Aa* | *TaPHS1-222T* | *TaMFT-A1b* | *TaPHS1-646G* | *TaPHS1-666A* | 52.80% | 52.40% | 60.30% |
| 326 | Neimai 9 | *TaSdr-A1a* | *TaSdr-B1b* | *Tamyb10-A1a* | *Tamyb10-B1a* | *Tamyb10-D1a* | *Vp-1Ba* | *TaDFR-Bb* | *TaMKK3-Ab* | *GS34-7Ba* | *TaMFT-3Ab* | *TaPHS1-222T* | *TaMFT-A1b* | *TaPHS1-646G* | *TaPHS1-666A* | 36.90% | 85.30% | 59.10% |

*—* Missing data
